# Synthesis of Core‐Functionalised Naphthalenediimides from Naphthalenetetracarboxylic Dianhydride using a Vibratory Ball Mill: Bromination, Imidization and Heck‐Type Reactions

**DOI:** 10.1002/chem.202403217

**Published:** 2025-01-23

**Authors:** E. M. Dodson, T. E. Lawson, J. Lai‐Morrice, H. Emerit, D. P. Guest, L. A. Panther, R. Gonzalez‐Mendez, S. M. Roe, C. A. I. Goodall, M. C. Bagley, J. Spencer, B. W. Greenland

**Affiliations:** ^1^ Department of Chemistry Arundel Building 305 School of Life Sciences University of Sussex, Falmer BN1 9QJ Brighton UK; ^2^ 113 Botanicals 398 Montrose Avenue SL1 4TJ Slough UK; ^3^ Faculty of Engineering & Science FES Engineering & Science School Operations University of Greenwich Old Royal Naval College Park Row SE10 9LS London UK; ^4^ Sussex Drug Discovery Centre School of Life Sciences University of Sussex, Falmer BN1 9QG Brighton UK

**Keywords:** Green chemistry, mechanochemistry, ball mill, Heck, naphthalenediimide

## Abstract

The synthesis of 9 core‐functionalised naphthalene diimide (c‐NDI) residues is reported via a 3‐step synthesis from naphthalenetetracarboxylic dianhydride using only mechanochemical activation. Selective dibromination and subsequent diimidization were achieved for the first time in a vibratory ball mill, resulting in the key structural intermediate, 2,6‐dibromonaphthalenediimide (DBND), which is the basis for elaboration into a multitude of organic electronic materials. Our new synthesis of DBND is achieved in just 5 hours reaction time over two steps compared to typical solution state times of 24 hours. Subsequent Heck‐type cross coupling reactions, with a range of styrene residues, produced a series of c‐NDIs in good yields. The Heck‐type reactions are rapid (1.5 hours), require no additional heating or solvent and are tolerant of atmospheric moisture and air.

## Graphical abstract

Core‐functionalised naphthalene diimides (c‐NDIs) are an important component in organic electronics. Current methods for their production frequently require long reaction times, large quantities of solvent and inert conditions. Herein, we report the synthesis of a series c‐NDIs by a 3‐step synthesis from naphthalenetetracarboxylic dianhydride (NDI) using only environmentally sustainable mechanochemical methods and without excluding air or moisture.

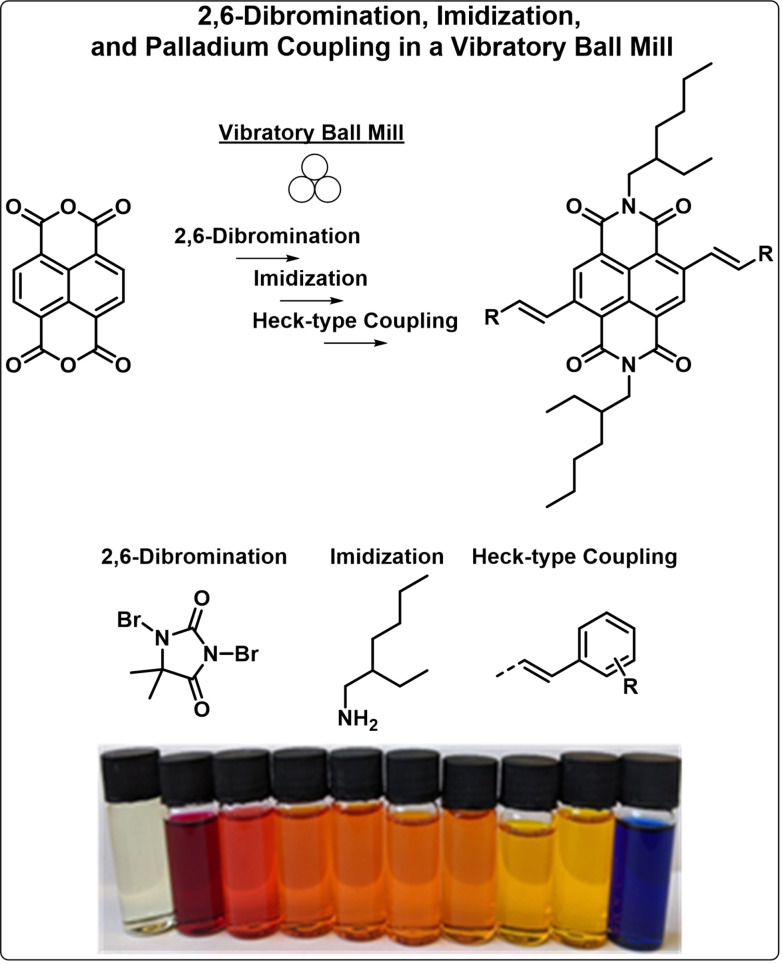

## Introduction

Harnessing the diverse properties of inexpensive molecules and materials to enable novel technologies underpins our modern world. However, until relatively recently, the impact on the environment of producing these chemicals was not an overriding concern when developing new manufacturing processes.^[1]^ Indeed, despite the positive benefits that large scale chemical manufacturing has made on society, the solvent‐heavy methodology typically used in synthesis is wasteful from many standpoints, for example: i) production of the solvent (often from petrochemical feedstocks); ii) transport and storage; iii) the energy required to heat the solvent during the reaction; iv) the energy required to separate the solvent from product; and v) disposal of the solvent. Aside from energy considerations, it has been estimated that the solvent in a reaction can account for over 80 % of the total mass of the waste produced during a single process.^[2]^

Efforts to reduce the quantity of solvent used in a synthesis are, therefore, of considerable interest.^[3]^ To this end, research in the area of mechanochemistry has increased dramatically in recent years. These synthetic processes, typically carried out using vibratory ball mills (VBM), planetary ball mills or extruders, have demonstrated that reactions can be efficiently completed using either minimal quantities of solvent, termed liquid assisted grinding (LAG),[^4^, ^5^, ^6^] or even in the complete absence of solvents. Friščić *et al*. suggested that the ratio of the total volume of solvent (μL) to reagent weight (mL) to be used in a reaction, termed ‘η’, can be a convenient way to compare reactions across multiple conditions.[^7^, ^8^] Thus, they suggest η=0 for solvent free, 0<2 for LAG, 2<12 for slurry and η values above 12 μL/mL being indicative of a solution state reaction.

Successful examples of mechanochemical transformations now cover a wide range of bond forming reactions and functional group interconversions; for example, in the synthesis of aminoesters,[^9^, ^10^, ^11^] hydrazones,^[12]^ peptides,^[13]^ and nitrones.^[14]^ In addition, it has been demonstrated that metal catalysed reactions such as click chemistry,^[15]^ and coupling methods such as Suzuki,[^4^, ^5^, ^16^] Sonogashira,[^17^, ^18^, ^19^] and Buchwald‐Hartwig aminations[^6^, ^20^, ^21^] proceed rapidly, in non‐inert atmospheres and without additional heating under VBM conditions.

Although less well developed than the small molecule reactions detailed previously, recent work has shown the syntheses of macromolecules and materials including polymers,^[22]^ metal organic frameworks (MOFs),[^23^, ^24^] and covalent organic frameworks (COFs)[^25^, ^26^] are also amenable to synthesis by mechanochemistry both through milling and extrusion methodologies. Reducing or removing solvents in the production of bulk materials is a key challenge as a consequence of their greater scales of production when compared to fine chemicals.

**Scheme 1:**
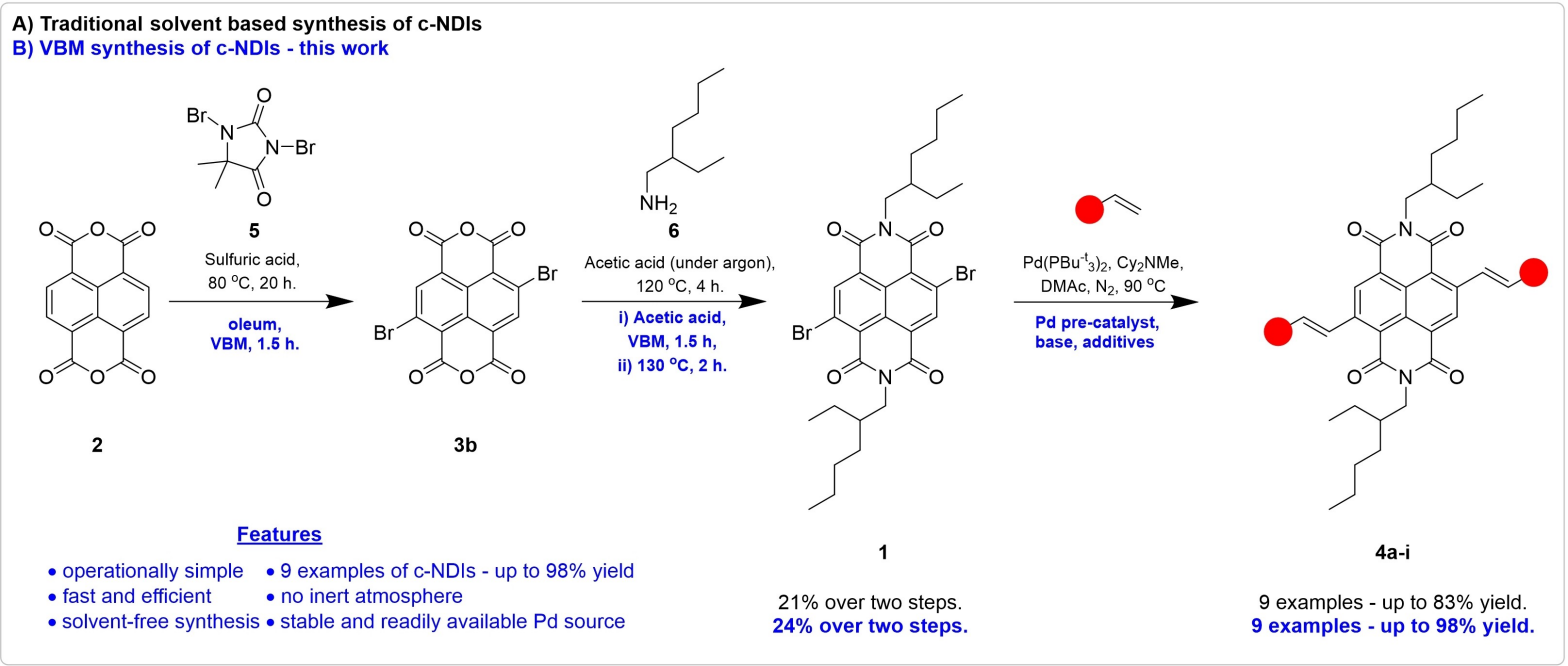
A) Solvent‐based synthesis of c‐NDIs; B) VBM synthesis of c‐NDIs.

Our work in this area has focussed on the mechanochemical synthesis of core‐functionalised naphthalene diimide (c‐NDI) residues.[^27^, ^28^] c‐NDIs are a well established components in organic electronics,^[29]^ solar cell technology,^[30]^ and in artificial photosynthesis.^[31]^ We demonstrated that 2,6‐dibromonaphthalenediimide (**1**) could undergo rapid (<1.5 h) and efficient solvent free Pd cross coupling reactions in a VBM under atmospheric air and moisture to produce a wide range of structurally related products. In this previous work, the key dibromo NDI intermediate, **1**, was accessed through conventional two‐step solution phase synthesis whereby naphthalenedianhydride (NDA), **2** was dibrominated to produce **3 b** prior to carrying out the imidization reaction to furnish **1** (Scheme 1A).

Despite the ubiquity of dibrominated NDIs as starting materials in the literature, this synthesis is often hampered by poor selectivity during the dibromination step, resulting in mixtures containing various poly‐brominated products. This is further complicated as the resulting mixtures can be difficult to purify, necessitating further solvent use. This issue is typically overcome by completing the imidization on the crude poly‐brominated product mixture to produce soluble products allowing isolation of **1** through conventional means (i. e. recrystallisation or column chromatography). In addition, these solution state reactions require long reaction times (20 h and 4 h for each step, respectively), forcing conditions (between 80 and 120 °C) and significant quantities of hazardous solvents (e. g. oleum). Thus, the production of **1** through more rapid mechanochemical means with the concomitant reduction in wasteful solvents is an appealing target.

Herein, we demonstrate the three‐step synthesis of c‐NDIs (**4 a**–**i**) from NDA **2** using only mechanochemical means. The synthesis of **1** was achieved in 24 % over two steps (Scheme 1B), which may be compared to those typically reported for this same synthesis in solution (e. g. 14–28 %).[^27^, ^32^, ^33^, ^34^, ^35^, ^36^] However, in our new synthesis, the reaction proceeded in just 5 h compared to 8–36 h total reaction time using conventional solution state reactions. The core functionalisation was carried out to produce a total of 8 structurally‐related products using a Pd catalysed Heck‐type reaction in up to 98 % isolated yield. Heck‐type couplings have not been demonstrated in the solid state for NDIs previously. Comparable studies by Liu *et al*.^[37]^ in solution required DMF as solvent, at 90 °C, under a nitrogen atmosphere for completion to give yields in the range 45–83 %.

## Results and Discussion

### Optimization of Studies for Selective Bromination of NDA

**Scheme 2:**
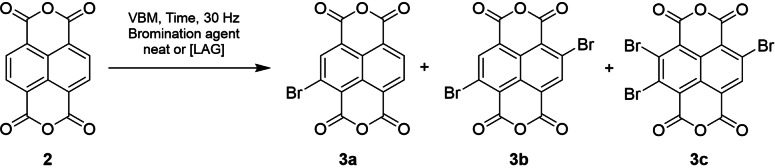
Synthesis of brominated NDAs using a VBM.

Considering the utility of dibrominated NDI molecules in materials chemistry, it is perhaps unsurprising that it has become a well‐reported reaction, with a particular focus on the improving the selectivity to produce the prized 2,6‐dibrominated isomer **3 b**. Typically, brominating agents such as dibromoisocyanuric acid (DBCA),[^32^, ^33^, ^34^, ^35^, ^38^, ^39^, ^40^] tribromoisocyanuric acid (TBCA),^[41]^ or dibromodimethylhydantoin (DBDMH) **5**[^27^, ^36^, ^42^] are employed as the bromine source with a strong Brønsted acid used as the solvent. However, the variable reported yields and the lack of consensus over the most efficient conditions suggest this is a capricious reaction,[^35^, ^36^, ^43^] a conclusion supported during our own studies into the solution phase synthesis of this molecule.^[27]^ Therefore, with these solution state protocols in mind, we set out to find suitable conditions to carry out the selective dibromination of NDA in a VBM (Scheme 2).

As a consequence of the known difficulties in isolating **3 b**, we elected to interrogate the conversion and approximate product mixture by ^1^H NMR spectroscopy. This is possible because the target molecule **3 b** presents a defined singlet at δ=8.80 ppm, which is distinct in terms of chemical shift and/or splitting pattern compared to side products with differing levels of bromination (see figure S1 in the supporting information). Rapid examination of crude product mixtures by ^1^H NMR spectroscopy is naturally facilitated during this VBM synthesis as the crude reaction mix can be prepared directly for analysis without residual solvent obscuring the signals of interest.

Initial studies using *N*‐bromosuccinimide (NBS)^[44]^ as the brominating agent (Table 1 entries 1 and 2) showed essentially no conversion to the target dibrominated species. Studies using TBCA (entry 3) or DBCA at varying loading levels were more encouraging and showed that this reaction was feasible. However, despite screening a range of LAGs and reaction times with DBCA, we were unable to achieve a conversion of more than 49 % to **3 b** (entries 4 to 15).

**Table 1:** Optimisation of bromination reactions and structures of brominating agents.

Entry	Bromination Agent		LAG	ML^[a]^	MC^[b]^	Time	^1^H NMR molar % of			
	Name	Equivalents				(min)	**2**	**3 a**	**3 b**	**3 c**
1	NBS	2	Neat	20	1	90	100	0	0	0
2	NBS	2	H_2_SO_4_	20	1	90	95	5	0	0
3	TBCA	0.5	oleum	20	1	180	76	0	24	0
4	DBCA	1	Neat	20	1	90	100	0	0	0
5	DBCA	2	H_2_SO_4_	20	1	90	96	4	0	0
6	DBCA	2	H_2_SO_4_	20	1	180	90	3	7	0
7	DBCA	3	H_2_SO_4_	20	1	180	83	15	2	0
8	DBCA	5	H_2_SO_4_	20	1	90	88	2	10	0
9	DBCA	1.4	oleum	20	1	180	17	0	49	34
10	DBCA	0.5	oleum	20	1	180	75	2	15	7
11	DBCA	1	oleum	20	1	180	49	0	32	19
12	DBCA	1	oleum	20	1	360	48	0	35	18
13	DBCA	2	oleum	20	1	180	0	0	16	84
14	DBCA	2	oleum	20	1	360	0	0	16	84
15	DBCA	3	oleum	20	1	180	0	0	0	2
16	DBDMH	1.4	H_2_SO_4_	20	1	90	100	0	0	0
17	DBDMH	1.4	oleum	20	1	180	51	0	49	0
18	DBDMH	1.4	oleum	8.5	2	90	12	0	72	16
19	DBDMH	1.6	oleum	8.5	2	90	0	0	81	19
20	DBDMH	1.6	oleum	10	2	90	0	0	79	21
21	DBDMH	1.6	oleum	20	2	90	0	0	89	11
22	DBDMH	1.6	H_2_SO_4_	20	2	90	95	0	5	0
23	DBDMH	1.6	oleum	10	3	90	3	11	67	19
Bromination Agents										
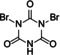		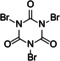		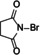			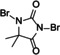			
DBCA		TBCA		NBS			DBDMH **5**			

[a] ML (mg.mL^−1^)=milling load. [b] MC=Mechanochemical conditions. Conditions 1: VBM 30 Hz, stainless steel grinding jar (10 mL), stainless steel milling ball(s) (1–2×10–12 mm diam.); Conditions 2: VBM 30 Hz, stainless steel grinding jar (50 mL), stainless steel milling ball(s) (8–14×12 mm diam.); Conditions 3: VBM 30 Hz, zirconium oxide grinding jar (10 mL), zirconium oxide milling balls (2×15 mm diam.).

Lastly, experiments using DBDMH **5** were carried out (entries 16 to 23) which rapidly showed promise in the selective formation of **3 b** especially using oleum,^[45]^ with the best conditions (entry 21) resulting in highly selective formation of the targeted dibrominated product (89 % of the product mix) in just 1.5 h. These reaction conditions represent a significant decrease in time compared to that typically used to complete this reaction (see for example: 20 h, this paper and 11 hours^[42]^), and a dramatic decrease in solvent (>65 %^[42]^). This reaction proceeds with η=1.11 in this case, placing the reaction within the range considered to be LAG.

### Imidization Reactions in the Solid State

**Scheme 3:**
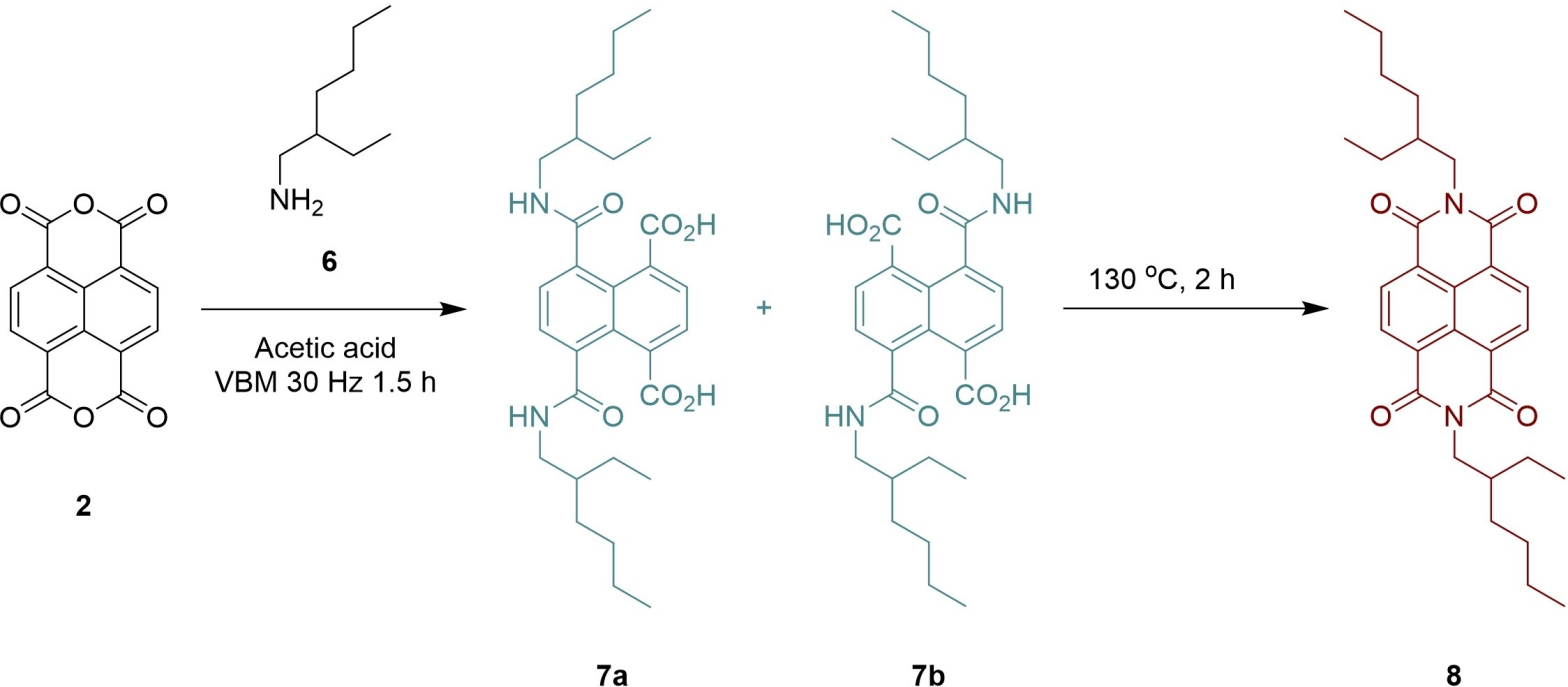
Imidization of NDA (**2**) to NDI (**8**).

**Figure 1:**
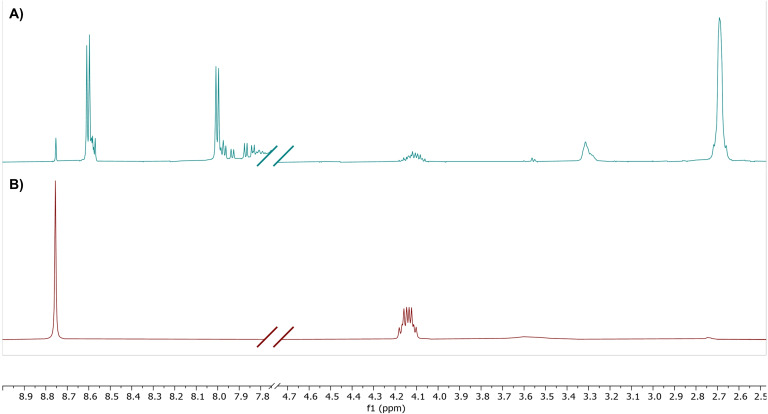
(A) ^1^H NMR spectrum of product mix from the addition of **2** and **6** after 90 min in a VBM; (B) ^1^H NMR spectrum showing production of **8** after heating the reaction mix (Scheme 3) in an oven for 2 h at 130 °C.

Initially, we sought to demonstrate the ability to carry out imidization of NDA (**2**) in the ball mill (Scheme 3). Such reactions have been investigated for related species by James *et al*. during their synthesis of pyrylene dyes.^[46]^ Addition of 2 equivalents of amine **6** to NDA **2** with acetic acid and exposing to VBM activation for 90 min resulted in multiple signals in the aromatic region upon ^1^H NMR spectroscopic analysis (Figure 1A) which appeared to be consistent with the production of ring opened amide containing products (**7 a** and **7 b**). This suggested whilst the attack of the amine on the anhydride occurred readily, these conditions did not provide the energy to enable ring closure and loss of water. This problem was readily overcome by heating the reaction mix in the milling vessel in an oven at 130 °C for 2 h, which resulted in a single signal in the aromatic region of the spectrum at δ=8.76 ppm, which is consistent with that of an authentic sample produced in our laboratory (Figure 1B). It should be noted that racemic amine (**6**) was used in all experiments and, therefore, all reported yields in this paper are for the diastereomeric mixture of products.

**Scheme 4:**
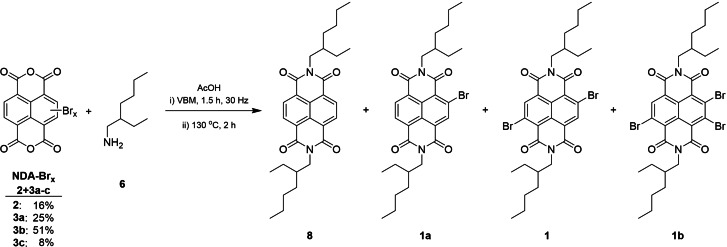
Model study synthesis for the imidization of brominated NDIs.

To verify that the imidization conditions used to produce **8** efficiently were transferable to brominated NDIs, the mechanochemical synthesis was briefly examined and optimised using crude mixtures of brominated NDA **3 a**–**c** which are readily available through solution phase synthesis. The relative proportions of each component in the mix were established by ^1^H NMR spectroscopic analysis of the starting mixture and are reported in Scheme 4.

**Figure 2:**
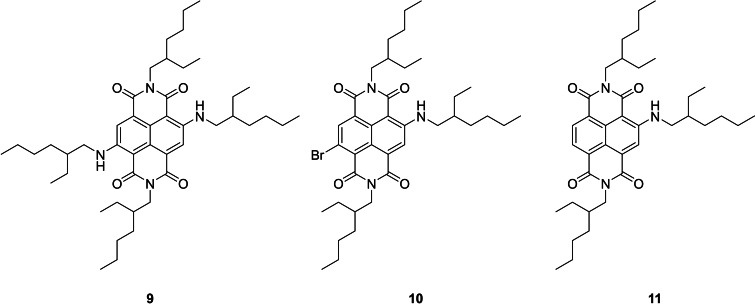
Structures of aminated core substituted NDI products isolated from the conditions used in entry 11, Table 2.

It can be seen, in Table 2, that the reaction is sensitive to the quantity of acetic acid used, with the yield increasing up to the addition of 60 μL (entries 1 to 4 up to 46 %), but then decreasing as the amount of acetic acid is increased further (down to 35 %, entries 5 and 6). Increasing the equivalents of amine with just 20 μL of AcOH improved the yield (entries 7 to 10 up to 41 %). Addition of a further excess of amine resulted in the formation of a vibrant red crude product mix, in contrast to the pale‐yellow products typical of these reactions. It became apparent that this was as a consequence of unintended nucleophilic aromatic substitution (S_N_Ar) of the bromide on the ring, of which the known products **9**, **10** and **11** (Figure 2) were isolated (entry 11).[^47^, ^48^] Optimising the quantities of both AcOH and amine resulted in the best yield of 57 % (entry 12, η=0.35, LAG conditions). In a control experiment we mixed by hand the liquid reagents and solid NDA‐Br_x_ and heated without milling for 3.5 h at 130 °C. This resulted in a dramatic decrease in the isolated yield to just 23 %, showing the necessity for the initial milling step in this reaction.

**Table 2:** Optimisation of conditions for the imidization of brominated NDIs.

Entry^[a]^	Amine (equiv.)^[b]^	AcOH (μL)	Isolated Yield (%)^[c]^
1	2	0	20 %
2	2	20	33 %
3	2	40	33 %
4	2	60	46 %
5	2	80	39 %
6	2	100	35 %
7	1.6	20	13 %
8	1.8	20	21 %
9	2.2	20	36 %
10	2.4	20	41 %
11	5	20	Core Aminated NDIs (see Figure 2)
12	2.4	60	57 %
13^[d]^	2.4	60	23 %

[a] Reactions were carried out on 100 mg of the mixture **2+3 a**–**c**. [b] Calculated from the mean molar mass of the product mix in the starting material. [c] Calculated from the theoretical maximum according the quantity of **3 b** in the starting material. [d] No milling, only 3.5 heating at 130 °C.

With viable reaction conditions for the two‐step synthesis of **1** in a VBM established, we carried out the reactions sequentially, to produce a sample, through an entirely mechanochemical route, in 24 % yield. This is comparable to solution state synthesis of this molecule yet achieved using only LAG quantities of oleum and acetic acid with a total reaction time of just 5 h. Thus, we moved on to producing our targeted Heck coupled NDI products by mechanochemical means.

### Optimization of Studies for Heck Synthesis of c‐NDIs

**Scheme 5:**
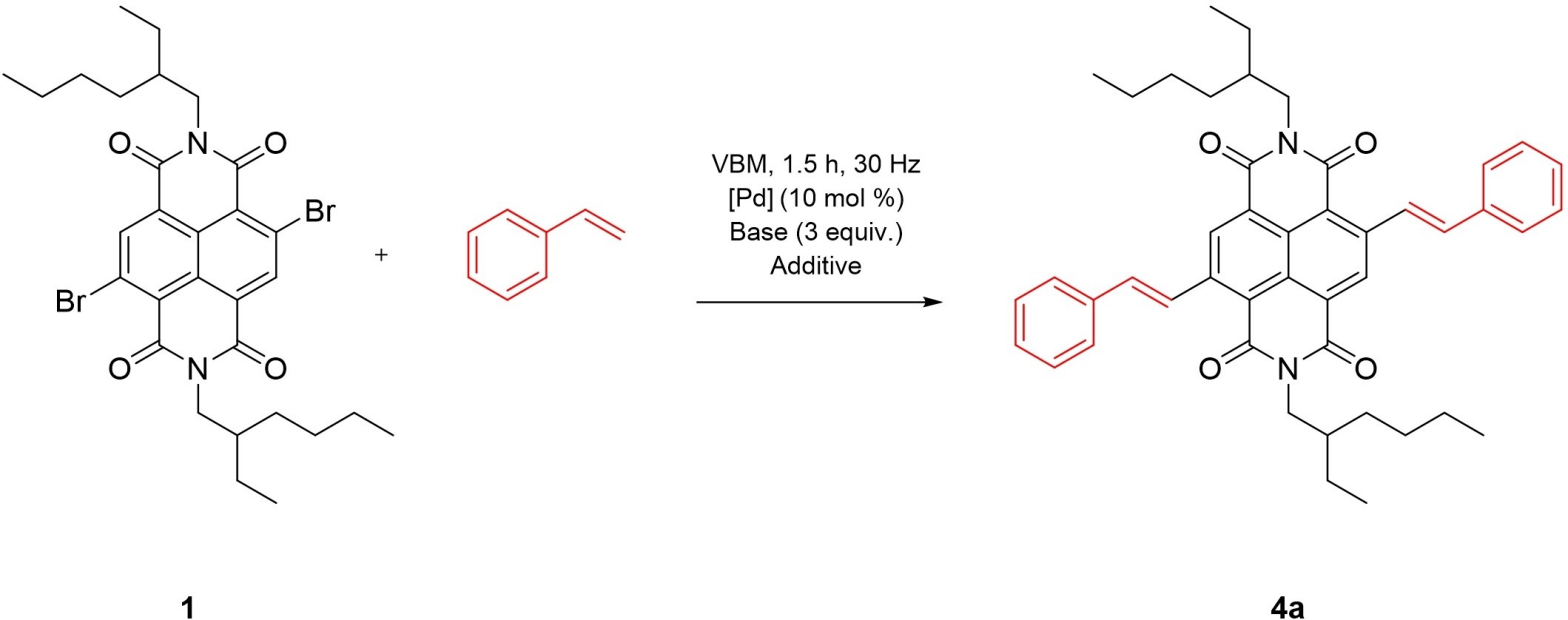
Synthesis of styrene functionalised NDI **4 a** under Heck‐type conditions in a VBM.

We have found previously in our investigations into solid state palladium coupling reactions that relatively low cost palladium sources (e. g. Pd(OAc)_2_) perform comparably to those with more complex, and expensive, ligand systems (e. g. d‐PEPPSI™‐Ipen). Therefore, we sought to begin our optimisation studies with these relatively inexpensive and accessible pre‐catalysts (Scheme 5 and Table 3).

**Table 3:** Optimisation of Heck‐type coupling conditions in a VBM.

Entry	[Pd]	Base [LAG]	Additive	Conversion to **4 a** by ^1^H NMR spectroscopy (%)
1	Pd(OAc)_2_	Cy_2_NMe	n/a	0
2	Pd(OAc)_2_	Triethanolamine	n/a	0
3	Pd(OAc)_2_	K_2_CO_3_	n/a	>10
4	Pd(PPh_3_)_4_	K_2_CO_3_	n/a	0
5	Pd(OAc)_2_	K_2_CO_3_	1,5 cod	0
6	Pd(OAc)_2_	K_2_CO_3_	PEG‐2000‐OH	>10
7	Pd(OAc)_2_	K_2_CO_3_	PEG‐2000‐OH NaCO_2_H	>10
8	Pd(OAc)_2_	K_2_CO_3_	NaCO_2_H Bu_4_NCl NaCl	45 %
9	Pd(OAc)_2_	K_2_CO_3_	Bu_4_NCl NaCl	>98 %

Loading was *ca*. 20 mg.mL^‐1^. Reactions were carried out for 1 h at room temperature and a pre‐catalyst loading of 10 mol %. Conversions were determined by ^1^H NMR spectroscopy in CDCl_3_. When used, the LAG is *ca*. 10 wt % of the total reaction mass.

Initial attempted Heck‐type reactions with liquid amine bases did not prove fruitful (entries 1 and 2). Therefore, we rapidly moved on to screening a high melting point inorganic base (K_2_CO_3_), where the expected product was observed in the ^1^H NMR spectrum of the reaction mixture albeit in low yield (*ca*. 10 %, entry 3, see SI figure S2 for typical crude NMR spectra for these optimisation reactions). Altering the Pd source to Pd(PPh_3_)_4_ was not successful, resulting in no conversion (entry 4).

For similar palladium couplings in the solid state, liquid‐assisted‐grinding (LAG) has been found to offer significant improvements by reducing particle size and allowing more efficient mixing of reagents.^[49]^ The common additive 1,5‐cyclooctadiene (COD) was tested (entry 5) but was found to inhibit the reaction, unlike in related processes.[^4^, ^6^]

We then explored addition of PEG‐2000‐OH and sodium formate which had been reported to assist in such reactions by reducing particle size, and stabilising Pd (0) species respectively,^[50]^ but this was also unsuccessful, giving low conversion (entries 6 and 7).

Tullberg *et al*. reported the Heck‐type synthesis of unnatural amino acids using three distinct additives: sodium chloride which acts as a solid support, with sodium formate and tetrabutylammonium chloride reportedly acting as phase‐transfer catalysts (entry 8).^[51]^ These conditions resulted in a significant increase in conversion to **4 a** of 45 %.

Further studies on this relatively complex reaction system showed that removal of the sodium formate (entry 9) resulted in essentially complete conversion of **1** to **4 a** in 1.5 h, with no solvent (η=0). These condition may be compared to typical solution state conditions in this Heck type reactions η=18,^[37]^ 90 °C,^[37]^ 16 h.^[52]^

Whilst these reactions were clearly efficient in terms of both time and solvent used, they were carried out on relatively small scales *c*. 100 mg. Prior to starting a full substrate scope we were interested to explore the scale of the reaction that could be achieved in 25 mL milling jars. Increasing the quantity of dibrominated starting material, **1**, to 200 and 500 mg sequentially required a stepwise increase in reaction time to 3 h and then 6 h to observe full conversion by ^1^H NMR spectroscopy. Isolated yields were 75 and 58 %, respectively, after column chromatography, resulting in more than 300 mg of product produced in one reaction.

### Substrate Scope for the Synthesis of c‐NDIs by Heck‐Type Coupling

**Scheme 6:**
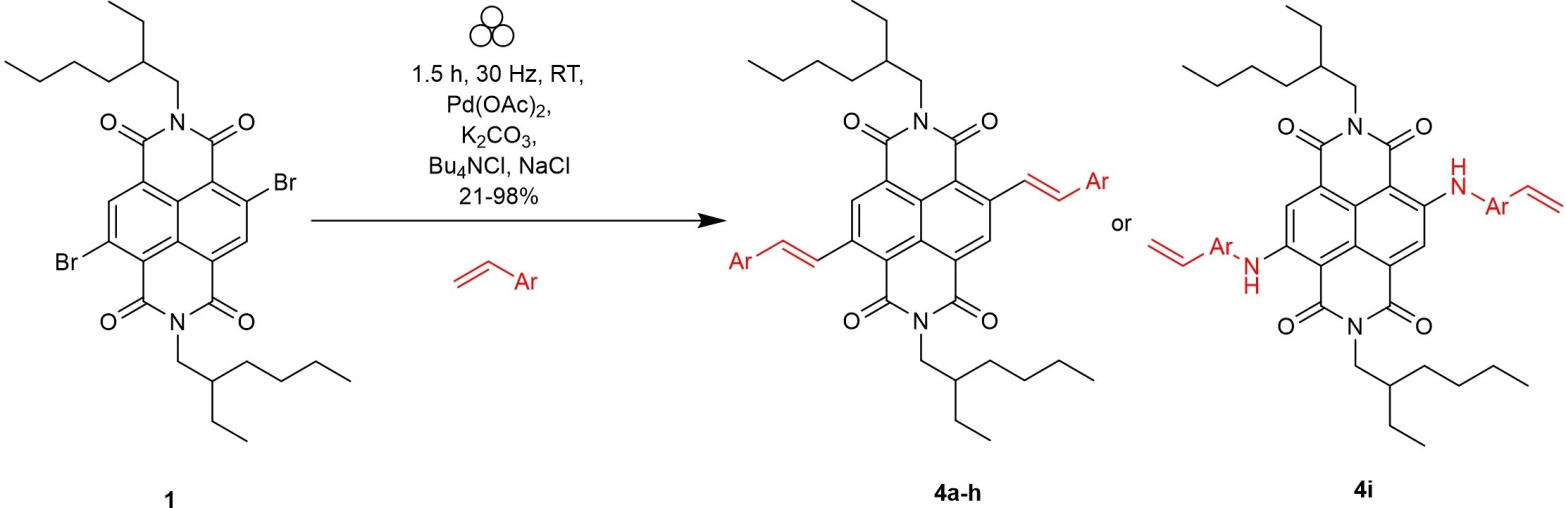
Synthesis of Heck‐type c‐NDIs.

**Figure 3:**
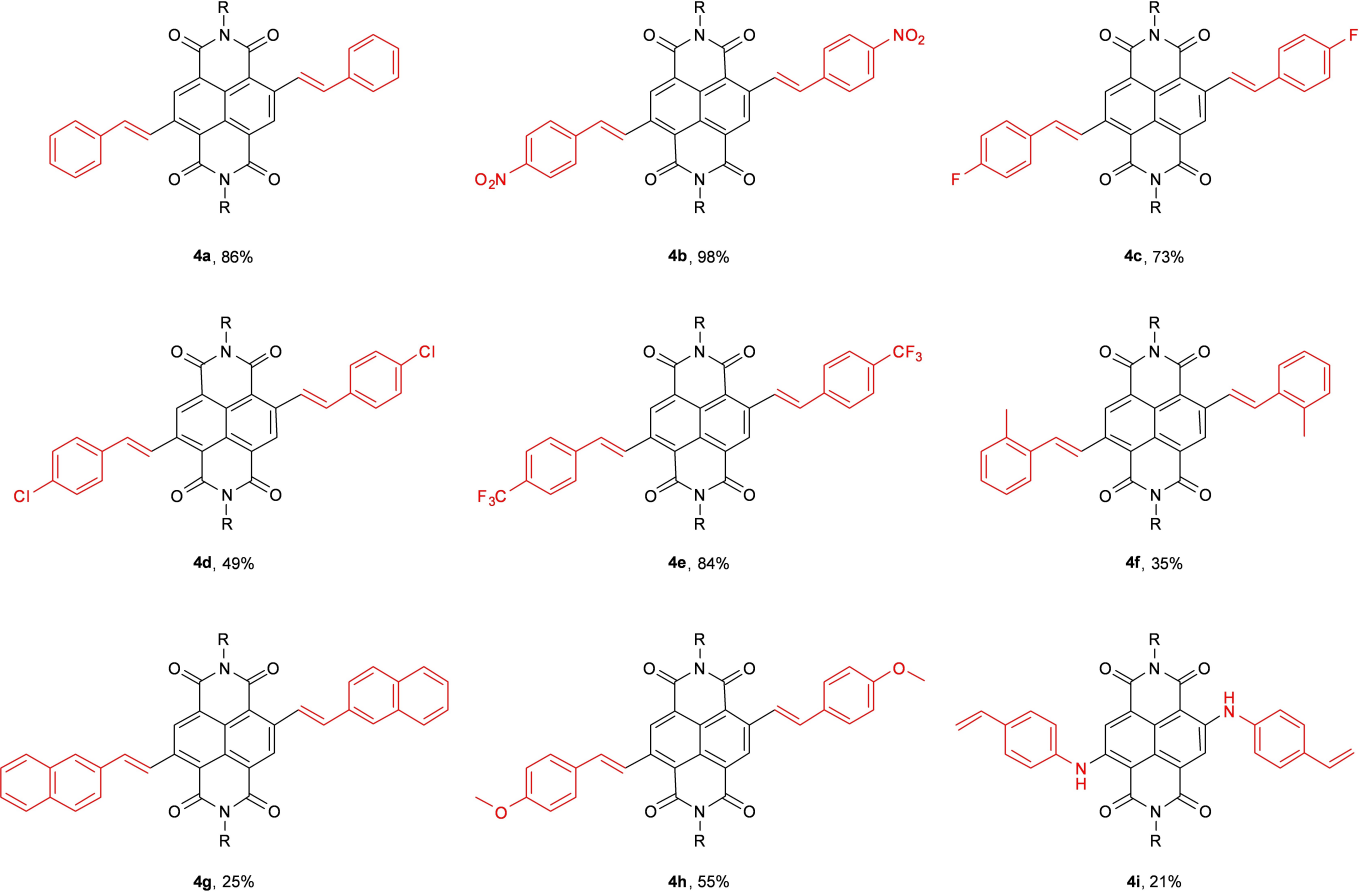
Yields for c‐NDI products synthesised *via* Heck‐type coupling using VBM methods, and structure of aminated product **4 i** formed under the same conditions. Isolated yields. R = 2‐ethylhexyl.

Initially, flash column chromatography was used to remove the excess styrene and additives. This technique is effective but uses large volumes of organic solvent, so as an alternative, a work‐up that avoided flash column chromatography was designed. The samples were washed with excess water to remove all the salts; dried with magnesium sulphate and precipitated from hot chloroform into hexane. The method was performed (Scheme 6) with styrene, 4‐methoxystyrene, 4‐nitrostyrene, 2‐vinylnaphthalene, 2‐methylstyrene, 4‐fluorostyrene, 4‐chlorostyrene, 4‐(trifluoromethyl)styrene and 4‐aminostyrene with the products of these reactions (**4 a**—**i)** shown in Figure 3.

It can be seen that, in general styryl residues with electron withdrawing substituents gave higher yields than those with more electron rich ring systems (e. g. nitrostyrene, **4 b**=98 % vs methoxystyrene, **4 h**=55 %). In addition, increasing steric bulk around the double bond served to reduce the yield (2‐methylstyrene product **4 f**=35 % vs styrene product **4 a** 86 %). It should be noted that attempts to carry out the coupling reaction with 4‐aminostyrene resulted in the formation of diaminated product (**4 i**) showing that in this case the Buchwald‐Hartwig type Pd coupling reaction was more favourable than the Heck‐type C−C bond forming reaction.

### Single Crystal X‐ray Analysis

**Figure 4:**
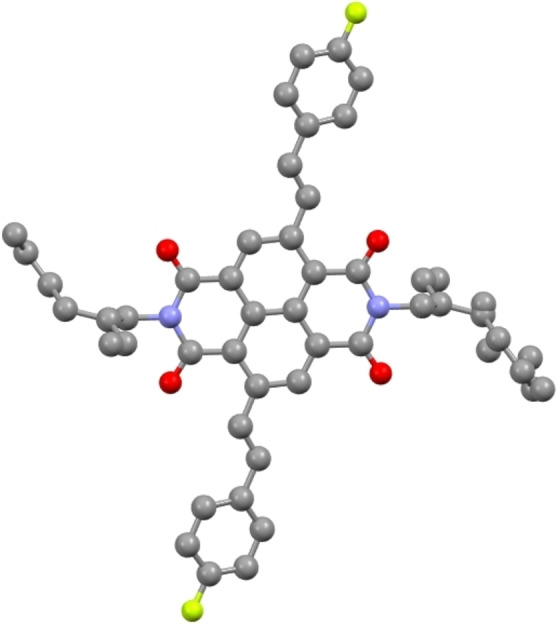
X‐ray crystal structure of c‐NDI **4 c**.

Slow diffusion using a dichloromethane/hexane solvent system yielded a single needle‐shaped crystal of fluoro‐substituted product **4 c** which was subjected to single crystal X‐ray crystallography (Figure 4).

The crystal packing for **4 c** was found to be a triclinic crystal system with P‐1 space group, comprising of three independent molecules of **4 c**. Whilst there is some disorder in the branched alkyl chains on the imides, this result supported the 2,6‐dibromo NDI isomer was indeed synthesised and confirms the trans‐substitution of the styryl group onto the naphthalene core. In the solid state, the phenyl rings are distinct, with one being essentially co‐planar with the naphthalene core (2.3°) whilst the other was significantly out of plane with the naphthalene core (20.0°). The coupling constant between the vinylic protons on the double bonds in **4 c** is *ca*. 16 Hz as measured during ^1^H NMR spectroscopic analysis, which is typical for the *trans* configuration of the product as observed in the solid‐state structure. This value is also observed for each of the Heck‐type coupled products in Figure 3, supporting the proposed *E*‐stereochemistry of each.

### UV/Vis Absorption Properties of c‐NDIs

**Figure 5:**
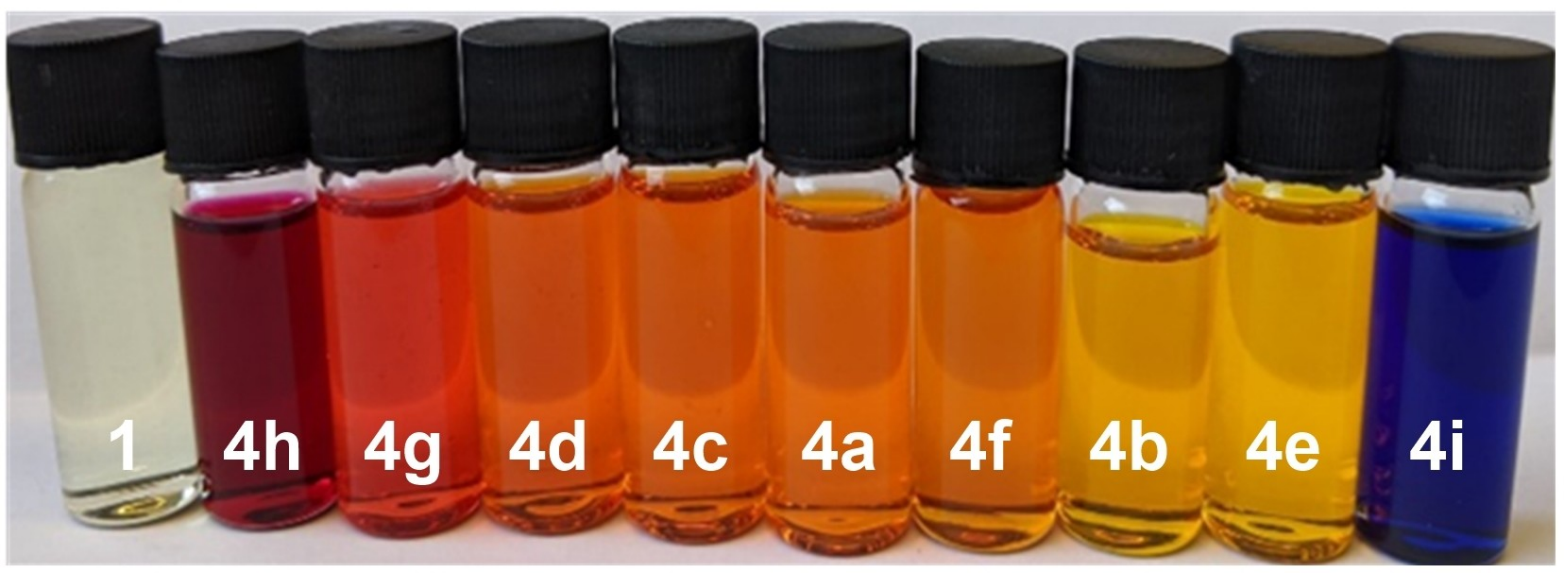
Photograph of 1 mM solutions of the starting material **1** (left) and cross‐coupled products **4 a**–**4 i** in chloroform under white light.

**Figure 6:**
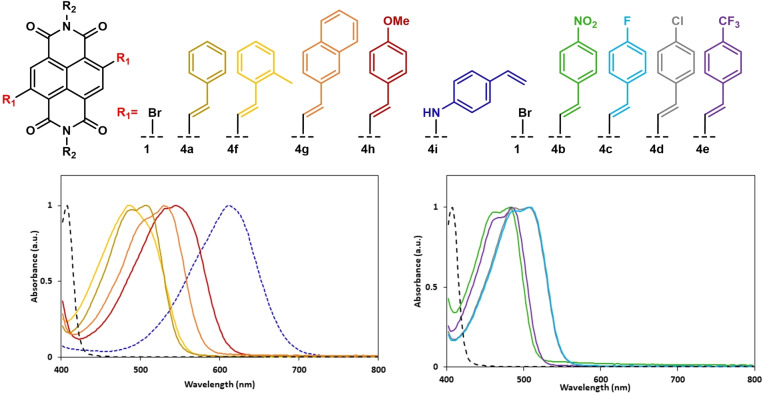
Normalized photophysical data of c‐NDIs as observed *via* UV/Vis absorption spectroscopy (0.2 mM, CHCl_3_) for Heck coupled products **4**. R_2_=2‐ethylhexyl.

A photograph of prepared samples (**1** and **4 g** to **4 i**) at 1 mM concentration can be seen in Figure 5 with the UV/Vis data provided in Table 4 and Figure 6. In agreement with previous studies,[^27^, ^37^, ^53^] the electronic nature of the coupling partner was found to have a dramatic influence on the electronic absorption properties of the c‐NDI products (Table 4).

**Table 4:** Selected UV‐Vis absorbance values for **1 b** and c‐NDIs **4 a**–**4 i** recorded at 0.2 mM in chloroform.

c‐NDI	π‐π* transition λ_abs_/nm	ICT transition λ_abs_/nm	c‐NDI	π‐π* transition λ_abs_/nm	ICT transition λ_abs_/nm
**1 b**	357, 364	404	**4 e**	327	484
**4 a**	336	506	**4 f**	336	485
**4 b**	329	482	**4 g**	345	530
**4 c**	334	509	**4 h**	359	542
**4 d**	338	505	**4 i**	333	610

All compounds, including dibromo starting material (**1** dashed black line), exhibit a high energy absorption with λ_max_ in the range 320–360 nm (Table 4), which has been attributed to the NDI π‐π* transition.[^54^, ^55^] The c‐NDIs also exhibited a lower energy band with λ_max_ between 480–610 nm, which corresponds to the intramolecular charge transfer (ICT) transition.^[40]^ Comparison of the ICT transition value of the starting material **1** to those measured for the c‐NDIs **4 a**–**4 e** shows that the addition of a phenyl ring conjugated through a double bond to the naphthalene core induces a bathochromic shift. Electron donating substituents (e.g 4‐methoxystyrene) shifted peak absorbance to a higher wavelength (e. g. 542 nm), whereas electron withdrawing substituents (e.g 4‐nitrostyrene) shifted λ_max_ to a lower wavelength (482 nm). The λ_max_ for **4 i** where the aromatic group is attached to the naphthalene core through the nitrogen atom is 610 nm resulting in a distinct blue colour (Figure 5). This is within the range observed for related compounds synthesised previously in our group.^[25]^

## Conclusions

We have demonstrated the synthesis of DBND (**1**) over two steps in the solid state using a vibratory ball mill for each step. The synthesis is completed in comparable yields to typical solution state reactions (24 % over two steps), using minimal solvent (η=1.11 and 0.35, LAG conditions) and is completed in a significantly reduced total reaction time (5 h in this work vs c. 24 h in solution). In addition, we have been able to carry out palladium catalysed solvent free Heck‐type reactions at room temperature (η=0). In contrast to typical Heck‐type reactions, these proceed without requiring an inert atmosphere and generate a series of styrene functionalised NDIs with a range of electronic absorbance properties. We have shown that the reaction can be scaled up to produce synthetically useful quantities (over 300 mg in one run) of pure product in 6 h in 25 mL milling jars. This work provides further evidence for the utility of VBM synthesis using minimal solvent for a broad range of reactions which, in this case, target molecules with potential for use in organic electronics.

## Associated Content

The Supporting Information is available free of charge. Further optimization studies, detailed synthetic procedures, compound characterization, spectral data, and crystal information (PDF).

Deposition Number CCDC‐2379216 for **4 c** contains the supplementary crystallographic data for this paper. These data are provided free of charge by the Cambridge Crystallographic Data Centre.

## Author Contributions

B. W. G. conceived and supervised the work assisted by J. S., M. C. B. and D. P. G. Synthesis and photophysical studies of c‐NDIs were carried out by T. E. L., J. L.‐M. and E. M. D. with H. E. carrying out initial bromination optimization studies. E. M. D. grew the single crystal of **4 c** for X‐ray analysis. R. G.‐M. and C. A. I. G. conducted MS analysis of the samples. S. M. R. conducted X‐ray analysis. B. W. G., L. A. P., J. L.‐M. M. C. B. and J. S. drafted the manuscript through discussion with all the authors, who approved the final version of the manuscript.

## Conflict of Interests

The authors declare no conflict of interest.

1

## Supporting information

As a service to our authors and readers, this journal provides supporting information supplied by the authors. Such materials are peer reviewed and may be re‐organized for online delivery, but are not copy‐edited or typeset. Technical support issues arising from supporting information (other than missing files) should be addressed to the authors.

Supporting Information

## Data Availability

The data that support the findings of this study are available in the supplementary material of this article and can be found at the following link: ***INTSERT LINK FOR SI HERE***.
