# Supplementary material for: Synthesis of Core‐Functionalised Naphthalenediimides from Naphthalenetetracarboxylic Dianhydride using a Vibratory Ball Mill: Bromination, Imidization and Heck‐Type Reactions
Source: Chemistry. 2025 Jan 23;31(9):e202403217. doi: 10.1002/chem.202403217 (PMC11814503; doi:10.1002/chem.202403217)
Supplement: Supplementary file 1 — Supporting Information [file CHEM-31-e202403217-s001.pdf]

Chemistry–A European Journal

Supporting Information

Synthesis of Core-Functionalised Naphthalenediimides from Naphthalenetetracarboxylic Dianhydride using a Vibratory Ball Mill: Bromination, Imidization and Heck-Type Reactions

E. M. Dodson, T. E. Lawson, J. Lai-Morrice, H. Emerit, D. P. Guest, L. A. Panther, R. Gonzalez-Mendez, S. M. Roe, C. A. I. Goodall, M. C. Bagley, J. Spencer, and B. W. Greenland*

Synthesis of core-functionalised naphthalenediimides from naphthalenetetracarboxylic dianhydride using a vibratory ball mill: bromination, imidization and Heck-type reactions.

E. M. Dodson[a], T. E. Lawson[a], J. E. Lai-Morrice[a], H. Emerit[a], D. P. Guest[b], L. A. Panther[a], R. Gonzalez-Mendez[a], M. Roe[a], C. A. I. Goodall[c], M. C. Bagley,[a] J. Spencer[a,d] and B. W. Greenland*[a]

Table of Contents

S1.0 Experimental Details p2

S1.1 General Procedures p2

S1.2 Synthetic Procedures p3

S2.0 Characterization Data p13

S3.0 Crystallography Data p37

S4.0 References p57

S1.0 Experimental Details

S1.1 General Procedures

Reagents were purchased from Merck, Fisher Scientific UK Ltd, Tokyo Chemical Industry UK Ltd or Fluorochem Ltd, and used without further purification. The ball milling reactions were carried out in a Retsch MM400 vibratory ball mill (VBM) operating at 30 Hz. Milling load is defined as the sum of the mass of the reactants per free volume in the jar.

Unless stated otherwise, purifications were performed via flash column chromatography on silica gel (RediSep® Rf Silica Gel Disposable Flash Columns, 40–60 micron) on a Teledyne ISCO CombiFlash Lumen apparatus. Analytical thin layer chromatography (TLC) was performed on silica gel 60 F254 (Merck).

UV-Vis sample details: 1 mM stock solution of samples dissolved in $CHCl_3$ (Figure S3) and diluted to 0.2 mM for UV-vis measurements. UV-Vis measurements (300-900nm) were recorded at room temperature on a Perkin Elmer Lambda 25 double beam spectrometer. ^{1}H (600 MHz) and ^{13}C NMR (150 MHz) spectra were recorded on a Varian VNMRS 600 MHz spectrometer, at room temperature, using the residual protic solvent signal in the deuterated solvent for calibration (chloroform-d at 7.26 ppm or DMSO-d_6 at 2.50 ppm). Chemical shifts are reported in ppm. Spin multiplicities are reported as a singlet (s), doublet (d), triplet (t) or multiplet (m), with coupling constants (J) given in Hz, where applicable.

High Resolution Mass Spectrometry (HRMS) data for all samples were obtained by Dr Iain Goodall of the University of Greenwich Mass Spectrometry Service using a Waters (Wilmslow, UK) Synapt G2 Q-ToF HRMS. Chromatographic characterisation was provided by a Waters H-class UPLC pumping module with heated column and auto-sampler, running a reversed-phase gradient.

Single Crystal Growth:

Crystals of compound **4c** were grown from dichloromethane and hexane using the slow diffusion method at room temperature.

S1.2 Synthetic Procedures

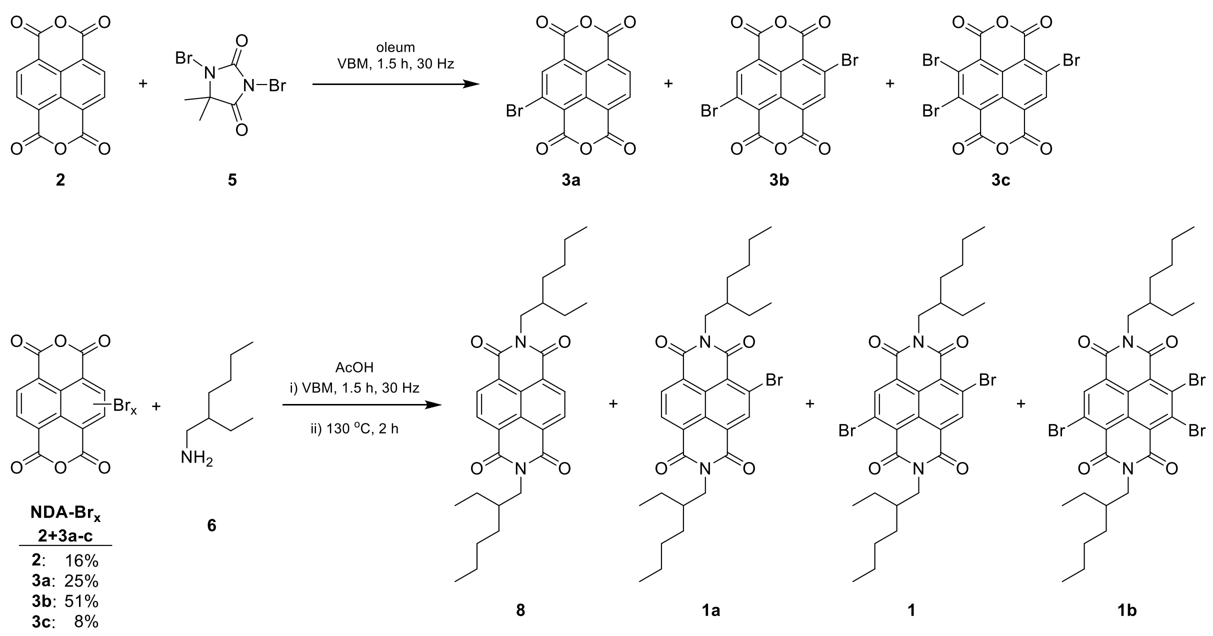

Scheme S1: Synthesis of N,N'-bis(2-ethylhexyl)-2,6-dibromo-1,4,5,8-naphthalenetetracarboxylic acid, **1**

Modified literature procedure by T. Govindaraju and co-workers was followed,[1] details outlined below:

Solution state synthesis of 2,6-dibromo-1,4,5,8-naphthalene tetracarboxylic acid **3b:**

Into a 500 mL round bottomed flask, naphthalene tetracarboxylic acid (NDA) **2** (30.02 g, 0.11 mol) was carefully added to stirring conc. sulphuric acid (18 M, 300 mL) at room temperature (CAUTION, strong acid). 1.25 equivalents of 5,5-dimethyl-1,3-dibromohydantoin **5** (40.02 g, 0.14 mol) were added in four portions over 1 hr. The reaction was stoppered loosely and heated to 80°C for 20 h, where the mixture turned from beige to bright yellow with a red gas produced. The hot mixture was then poured directly onto 3L of crushed ice which melted to give a volume of approximately 1.5 L of water whereupon the resulting yellow precipitate was collected in-vacuo on a low porosity sinter. The solid was washed with MeOH (300 mL) followed by water (300 mL) and dried in an oven at 40 °C under vacuum for 72 h to yield a mixture (47.4 g) of 2,6-dibromo-1,4,5,8-naphthalenetetracarboxylic acid **3b** and NDA **2**. 32:68 ratio of **3b**:**2** was calculated from ^{1}H NMR data by integration of the signals at δ = 8.79 ppm: 8.71 ppm. The crude product was used in the next step of synthesis without purification due to its poor solubility.

Solution state imidization of **3b** to N,N'-bis(2-ethylhexyl)-2,6-dibromo-1,4,5,8-naphthalenetetracarboxylic acid, **1**:

Crude product **3b** (44.2 g) was added to acetic acid (300 mL) and stirred. 2-Ethylhexylamine **6** (47.0 g, 0.41 mol) was added in four portions over 30 min at room temperature. The reaction was then heated to 120°C for 4 hr, where a colour change in solution from yellow

to dark red was observed. Once the reaction was complete, confirmed by TLC (DCM:hexane v:v 70:30 R_f^{SM} = 0.00, R_f^{Prod} = 0.45), it was cooled and poured onto ice (2 L). The resulting bright orange precipitate was collected in vacuo on a low porosity sinter and washed with water (300 mL), MeOH (500 mL) where red washings were observed, and finally hexane (1 L) where orange washings were observed. The crude orange solid (44.32 g, 33:67 ratio of dibromo-NDI **1**: NDI **8** by ^{1}H NMR) was then dissolved in a minimum amount of boiling $CHCl_3$ (ca. 500 mL) and was poured, while hot, into rapidly stirring cold hexane (2 L) to remove non-brominated diimide impurities and the pale orange crude solid was collected in vacuo. The precipitation procedure was repeated to yield yellow solid (25 g, 90:10 ratio of dibromo-NDI **1**: NDI **8** by ^{1}H NMR). **1** was isolated by a recrystallization over two weeks in DCM, where it was transferred to the fridge after one week, to yield analytically pure product as a yellow fluffy needle-like crystalline solid (15.2 g, 21% over two steps). ^{1}H NMR (600 MHz, chloroform-d) δ 8.99 (s, 2H), 4.15 (m, 4H), 1.93 (m, 2H), 1.43 – 1.33 (m, 8H), 1.22-1.32 (m, 8H), 0.93 (m, 6H), 0.88 (m, 6H). ^{13}C NMR (151 MHz, chloroform-d) δ 161.6, 161.4, 139.5, 128.7, 128.1, 125.6, 124.4, 45.5, 38.1, 30.9, 28.9, 24.3, 23.4, 14.5, 10.9. HRMS-ESI: calcd for $C_{30}H_{37}Br_2N_2O_4$ $[M+H]^+$ 647.1042 found 647.1120. Characterization data in agreement with the literature.

<u>Mechanochemical synthesis of 2,6-dibromo-1,4,5,8-naphthalene tetracarboxylic acid **3b:**</u>

Napthalene tetracarboxylic acid dianhydride **2**, (100 mg, 0.37 mmol), 1,3-dibromo-5,5-dimethylhydantoin (171 mg, 0.60 mmol), oleum (300 µL, 5.89 mmol), and two 15 mm zirconium oxide milling balls were added to a 25 mL zirconium oxide milling jar. Milling load = 39.4 mg·mL^{-1}. The jar was closed and subjected to grinding in the vibrational ball mill for 1.5 hours at 30 Hz, then allowed to cool to room temperature. The jar was washed out with water (4 x 10 mL) into a 50 mL falcon tube. This was centrifuged at 4000 rpm for 15 minutes. The supernatant was decanted and methanol (20 mL) added. The sample was vortexes for 10 seconds, then centrifuged at 4000 rpm for 15 minutes. The supernatant was decanted. The residue was dried under reduced pressure to give a crude mixture of **3b** as a yellow solid (95 mg). Estimated amount of bromination products by ^{1}H NMR: NDA-H_4 **2** (3%), NDA-Br **3a** (11%), NDA-Br_2 **3b** (67%), NDA-Br_3 **3c** (19%).

	**δ (ppm)**	**Integration**	**% of total**
NDA-H_4, **2**	8.71	0.60	3%
NDA-Br, **3a**	8.81	1.00	11%
NDA-Br_2, **3b**	8.79	7.00	67%
NDA-Br_3, **3c**	8.78	0.60	19%

Table S1: ^{1}H NMR data for the mixture of crude bromination products. ^{1}H NMR (600 MHz, $CDCl_3$)

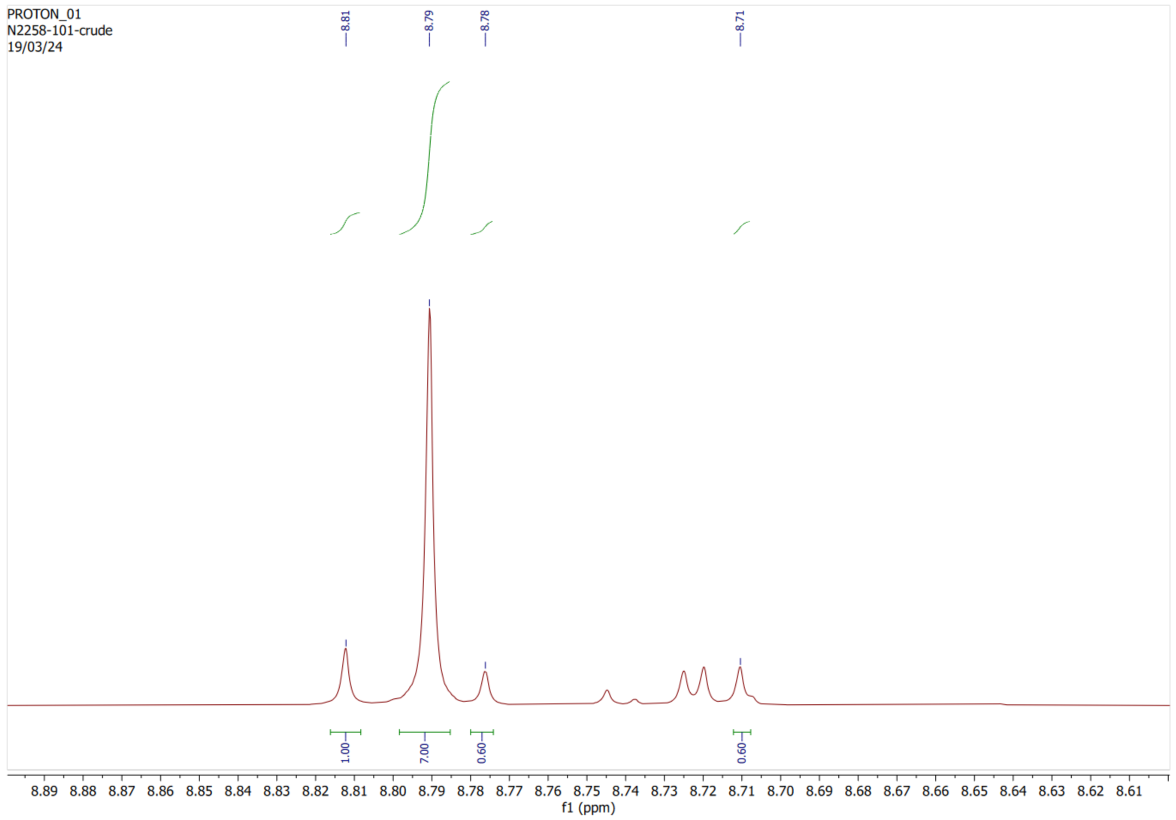

Figure S1: ^{1}H NMR spectra of the crude mixture of bromination products.

<u>Mechanochemical imidization of **3b** to N,N'-bis(2-ethylhexyl)-2,6-dibromo-1,4,5,8-naphthalenetetracarboxylic acid, **1**:</u>

The crude product **3b** (95 mg, 0.22 mmol), 2-ethylhexylamine (88 µL, 0.54 mmol), acetic acid (60 µL, 0.70 mmol), and two 15 mm zirconium oxide milling balls were added to a 25 mL zirconium oxide milling jar. Milling load = 9.6 mg·mL^{-1}. The jar was closed and subjected to grinding in the vibrational ball mill for 1.5 hours at 30 Hz, then heated in an oven at 130 °C for 2 hours. The jar was allowed to cool to room temperature and was washed out with dichloromethane and the washings concentrated to dryness. The crude product was solid loaded onto a 24 g Si column for chromatography. Eluting with a solvent gradient from 50-100% dichloromethane in petroleum ether. The appropriate fractions were collected and concentrated to dryness to give N,N'-bis(2-ethylhexyl)-2,6-dibromo-1,4,5,8-naphthalenetetracarboxylic acid **1** as a yellow orange solid (58 mg, 24% over two steps.). ^{1}H NMR (600 MHz, $CDCl_3$) δ 9.00 (s, 2H), 4.20 – 4.11 (m, 4H), 1.97 – 1.91 (m, 2H), 1.41 – 1.34 (m, 8H), 1.32 – 1.28 (m, 8H), 0.94 (t, J = 7.4 Hz, 6H), 0.88 (t, J = 7.0 Hz, 6H).

General Synthesis 1: Solid-State Heck Coupling.

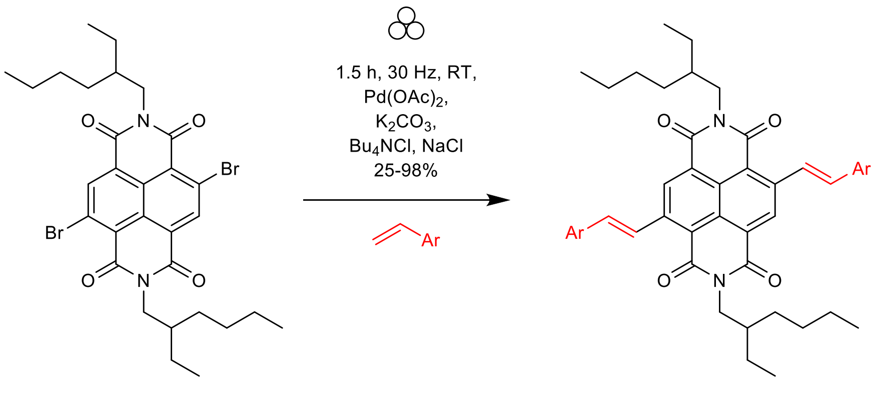

1 **4a-h**

Scheme S2: Synthesis of Heck-type c-NDIs

General protocol 1: N,N'-bis(2-ethylhexyl)-2,6-dibromo-1,4,5,8-naphthalenetetracarboxylic acid **1** (0.1 g, 0.154 mmol), 3 equivalents of the styrene derivative (0.465 mmol), 10 mol% palladium acetate (0.0035 g, 0.0154 mmol), potassium carbonate (0.064 g, 0.465 mmol), tetrabutylammonium chloride (0.052 g, 0.186 mmol) and sodium chloride (0.5 g, 8.56 mmol) were added to a 25 mL zirconium oxide grinding jar with two 15 mm zirconium oxide balls. The jar was then subjected to grinding for 90 minutes at 30 Hz. The crude solid was washed out with dichloromethane.

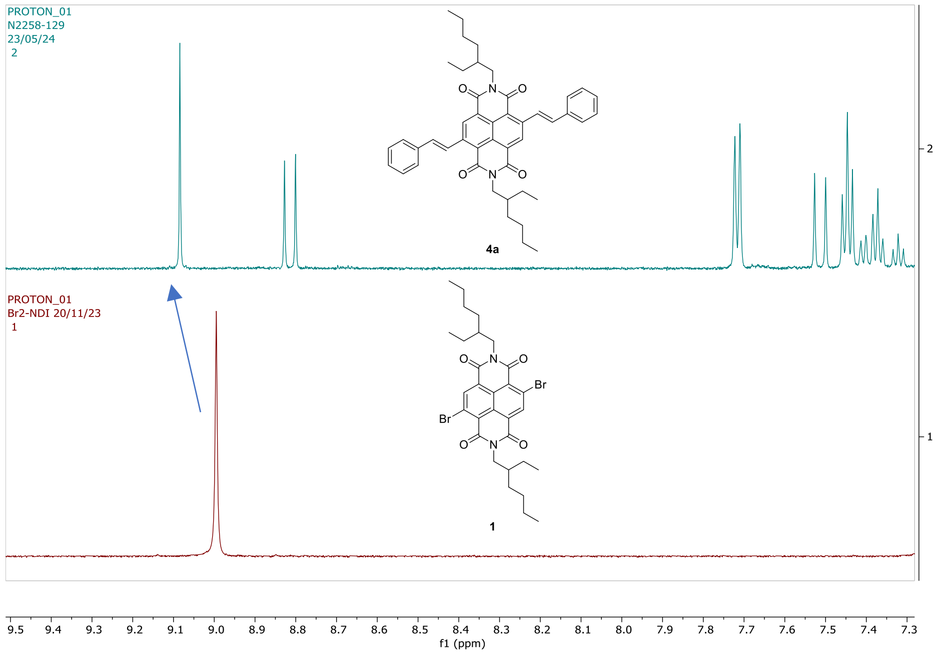

Figure S2: Stacked ^{1}H NMR of crude **4a,** directly from the ball milling reaction and pure **1**. The signal for the protons on the naphthalene core are observed at c. 9.0 ppm in **1** and c. 9.1 in **4a** showing conversion to be to be >98%.

2,7-Bis(2-ethylhexyl)-4,9-di((E)-styryl)benzo[lmn][3,8]- phenanthroline-1,3,6,8-(2H,7H)-tetraone (**4a**):

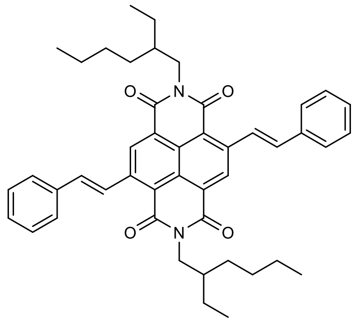

4a

The title compound was synthesised following general protocol 1 using styrene. The crude mixture was purified by flash column chromatography (1:4 petroleum ether:dichloromethane) to give the product as a red solid (0.092 g, 86% yield). ^{1}H NMR (600 MHz, $CDCl_3$) δ = 9.07 (s, 2H), 8.80 (d, 2H, J = 16.3 Hz), 7.71, (d, 4H, J = 7.6 Hz), 7.51 (d, 2H, J = 16.3 Hz), 7.45 (t, 4H), 7.37 (t, 2H), 4.16 (m, 4H), 1.96 (m, 2H), 1.39 (m, 8H), 1.31 (m, 8H), 0.94 (t, 6H), 0.88 (t, 6H). ^{13}C NMR (151 MHz, $CDCl_3$) δ = 164.1, 163.2, 142.5, 137.1, 136.7, 131.7, 129.2, 128.9, 127.7, 127.3, 126.9, 125.4, 120.1, 44.6, 37.9, 30.7, 28.6, 24.0, 23.1, 14.1, 10.7 ppm. HRMS-ESI: calculated for $C_{46}H_{51}N_2O_4$ $[M+H]^+$ 695.3849, found 695.3835.

General protocol 1 was followed to the solid-state Heck reaction at 2x (0.2 g of **1**) and 5x (0.5 g of **1**) scale. All reagents were scaled up accordingly. The reaction of 0.2 g of **1** was milled for a total of 3 hours. The reaction of 0.5 g of **1** was milled for a total of 6.5 hours.

2,7-Bis(2-ethylhexyl)-4,9-bis((E)-4-nitrostyryl)benzo[lmn][3,8]- phenanthroline-1,3,6,8(2H,7H)-tetraone (**4b**):

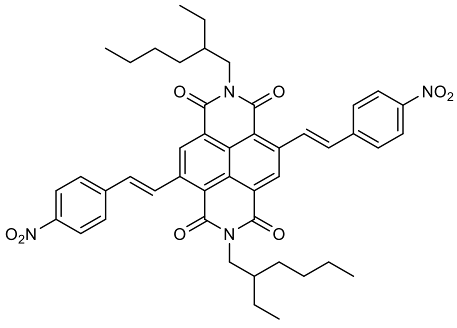

4b

The title compound was synthesised following general protocol 1 using 4-nitrostyrene. The sample was washed with excess water in a separating funnel, dried over magnesium sulphate, filtered to remove the salt; and then precipitated from hot chloroform into cold hexane, yielding an orange solid (0.120 g, >99% yield). ^{1}H NMR (600 MHz, $CDCl_3$) δ = 9.07 (s, 2H), 8.91 (d, 2H, J = 16.3 Hz), 8.32, (d, 4H, J = 8.2 Hz), 7.85 (d, 4H, J = 8.2 Hz), 7.50 (d, 2H, J= 16.3 Hz), 4.19 (m, 4H), 1.97 (m, 2H), 1.41 (m, 8H), 1.29 (m, 8H), 0.96 (t, 6H), 0.88 (t, 6H). ^{13}C NMR (151 MHz, $CDCl_3$) δ = 163.9, 162.8, 147.7, 142.8, 141.8, 134.2, 131.8, 131.4, 128.1,

127.5, 125.8, 124.3, 121.4, 44.8, 37.9, 30.7, 28.6, 24.0, 23.1, 14.1, 10.7 ppm. HRMS-ESI: calculated for $C_{46}H_{48}N_4O_8$ $[M+H]^+$ 784.3472, found 784.3440.

2,7-bis(2-ethylhexyl)-4,9-bis((E)-4-fluorostyryl)benzo[lmn][3,8]phenanthroline-1,3,6,8(2H,7H)-tetraone (**4c**):

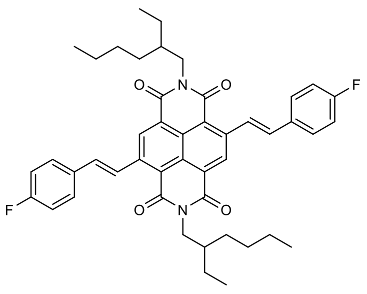

4c

The title compound was synthesised following general protocol 1 using 4-fluorostyrene (55.5 µL, 0.465 mmol). 4 was isolated as a bright orange solid after column chromatography (0.082 g, 73%), mp 232.1 – 233.6 °C. IR (neat) vmax/cm-1 1695 (C=C stretch), 1653 (C=O stretch), 1230 (C-F stretch), 1200 (C-N stretch), 821 (C-H bend). ^{1}H NMR (600 MHz, $CDCl_3$) δ = 8.99 (s, 2H), 8.69 (d, 2H, J = 16.5 Hz), 7.67 (t, 4H), 7.44 (d, 2H, J = 16.5 Hz), 7.13 (t, 4H), 4.14 (m, 4H), 1.94 (m, 2H), 1.43 – 1.29 (m, 16H), 0.94 (t, 6H), 0.89 (t, 6H). ^{13}C NMR (151 MHz, $CDCl_3$) δ = 164.0, 163.0, 142.3, 135.8, 132.8, 131.4, 129.4, 127.2, 126.5, 125.3, 120.4, 116.1, 115.9, 44.6, 37.8, 30.7, 28.6, 24.0, 23.1, 14.1, 10.7. HRMS-ESI: calculated for $C_{46}H_{49}N_2O_4F_2$ $[M+H]^+$ 731.3660, found 731.3624.

2,7-bis(2-ethylhexyl)-4,9-bis((E)-4-chlorostyryl)benzo[lmn][3,8]phenanthroline-1,3,6,8(2H,7H)-tetraone (**4d**):

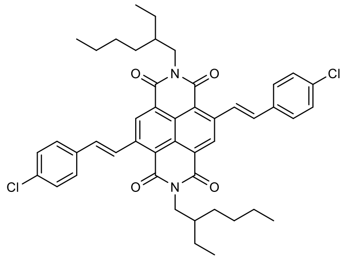

4d

The title compound was synthesised following general protocol 1 using 4-chlorostyrene (55.8 µL, 0.465 mmol). 5 was isolated as a bright red solid after column chromatography (0.057 g, 49%), mp 241.3 – 244.4 °C. IR (neat) vmax/cm-1 1697 (C=C stretch), 1654 (C=O stretch), 1201 (C-N stretch), 813 (C-Cl stretch). ^{1}H NMR (600 MHz, $CDCl_3$) δ = 9.04 (s, 2H), 8.77 (d, 2H, J = 16.5 Hz), 7.63 (d, 4H, J = 8.5 Hz), 7.44 (d, 2H, J = 16.5 Hz), 7.41 (d, 4H, J = 8.5 Hz), 4.17 (m, 4H), 1.96 (m, 2H), 1.42 – 1.30 (m, 16H), 0.95 (t, 6H), 0.89 (t, 6H). ^{13}C NMR (151 MHz, $CDCl_3$) δ = 164.0, 163.0, 142.2, 135.7, 135.1, 134.9, 131.5, 129.1, 128.8, 127.4, 127.2, 125.4, 120.6, 44.6, 37.8, 30.6, 28.5, 24.0, 23.1, 14.1, 10.6. HRMS-ESI: calculated for $C_{46}H_{49}N_2O_4Cl_2$ $[M+H]^+$ 763.3059, found 763.3033.

Synthesis of 2,7-bis(2-ethylhexyl)-4,9-bis((E)-4-(trifluoromethyl)styryl)benzo[lmn][3,8]phenanthroline-1,3,6,8(2H,7H)-tetraone (**4e**):

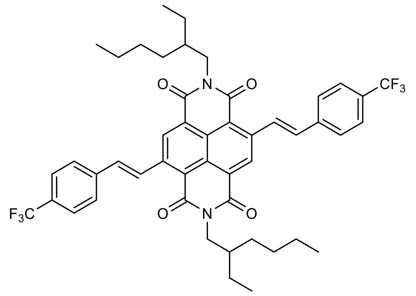

4e

The title compound was synthesised following general protocol 1 using 4-(trifluoromethyl)styrene (68.7 µL, 0.465 mmol). 6 was isolated as a bright orange solid after column chromatography (0.107 g, 84%), mp 235.2 – 238.1 °C. IR (neat) vmax/cm-1 1699 (C=C stretch), 1652 (C=O stretch), 1320 (C-F stretch), 1119 (C-N stretch), 824 (C-H bend). ^{1}H NMR (600 MHz, $CDCl_3$) δ = 9.07 (s, 2H), 8.85 (d, 2H, J = 16.5 Hz), 7.81 (d, 4H, J = 8.0 Hz), 7.70 (d, 4H, J = 8.0 Hz), 7.49 (d, 2H, J = 16.5 Hz), 4.18 (m, 4H), 1.97 (m, 2H), 1.43 – 1.32 (m, 16H), 0.96 (t, 6H), 0.90 (t, 6H). ^{13}C NMR (151 MHz, $CDCl_3$) δ = 164.0, 163.0, 142.1, 140.0, 135.3, 131.8, 129.5, 127.7, 127.4, 125.9, 125.9, 125.6, 121.1, 44.7, 37.9, 30.7, 28.6, 24.0, 23.1, 14.1, 10.7. HRMS-ESI: calculated for $C_{48}H_{49}N_2O_4F_6$ $[M+H]^+$ 831.3597, found 831.3568.

2,7-Bis(2-ethylhexyl)-4,9-bis((E)-2-methylstyryl)benzo[lmn][3,8]- phenanthroline-1,3,6,8(2H,7H)-tetraone (**4f**):

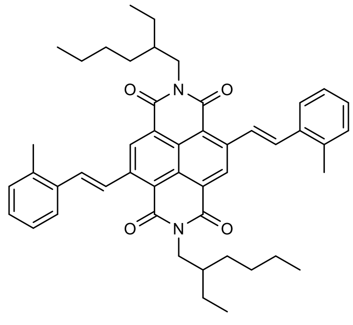

4f

The title compound was synthesised following general protocol 1 using 2-methylstyrene. The crude mixture was purified by flash column chromatography (1:4 petroleum ether:dichloromethane) to give the product as a red solid (0.039 g, 35%), mp 224.0 – 227.5 °C. IR (neat) vmax/cm-1 1692 (C=C stretch), 1648 (C=O stretch), 1222 (C-N stretch), 789 (C-H bend). ^{1}H NMR (600 MHz, $CDCl_3$) δ = 9.07 (s, 2H), 8.66 (d, 2H, J = 16.0 Hz), 7.84 (d, 2H, J = 7.5 Hz), 7.71 (d, 2H, J = 16.0 Hz), 7.30 (m, 6H), 4.17 (m, 4H), 2.54 (s, 6H), 1.98 (m, 2H), 1.40 – 1.30 (m, 16H), 0.95 - 0.88 (m, 12H). ^{13}C NMR (151 MHz, $CDCl_3$) δ = 164.1, 163.2, 142.9, 136.8, 135.7, 134.6, 131.9, 130.7, 128.9, 128.4, 127.3, 126.5, 126.5, 125.5, 120.7, 44.7, 37.8, 30.7, 28.6, 24.0, 23.1, 20.1, 14.1, 10.7. HRMS-ESI: calculated for $C_{48}H_{55}N_2O_4$ [M+H]+ 723.4162 found 723.4149.

2,7-Bis(2-ethylhexyl)-4,9-bis((E)-2-naphthalen-2-yl)vinyl)benzo- [lmn][3,8]phenanthroline-1,3,6,8-(2H,7H)-tetraone (**4g**):

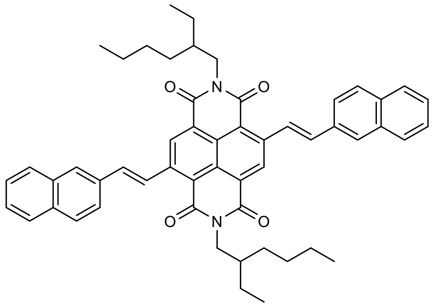

4g

The title compound was synthesised following general protocol 1 using 2-vinylnaphthalene. The sample was washed with excess water in a separating funnel, dried over magnesium sulphate, filtered to remove the salt; and then precipitated from hot chloroform into cold hexane, yielding a purple/red solid (0.031 g, 25% yield). ^{1}H NMR (600 MHz, $CDCl_3$) δ 9.05 (s, 2H), 8.89 (d, *J* = 16.3 Hz, 2H), 7.96 (s, 2H), 7.90 – 7.80 (m, 8H), 7.61 (d, *J* = 16.2 Hz, 2H), 7.51 – 7.46 (m, 4H), 4.18 (qd, *J* = 13.1, 7.4 Hz, 4H), 2.00 (p, *J* = 6.6 Hz, 2H), 1.47 – 1.40 (m, 8H), 1.37 – 1.31 (m, 8H), 0.98 (t, *J* = 7.4 Hz, 6H), 0.91 (t, *J* = 7.0 Hz, 6H). ^{13}C NMR (151 MHz, $CDCl_3$) δ = 164.2, 163.2, 142.5, 137.3, 131.6, 128.8, 128.7, 128.5, 127.8, 127.3, 127.1, 126.7, 126.6, 123.9, 44.6, 37.9, 30.7, 28.6, 24.1, 23.2, 14.2, 10.7 ppm (due to low concentrations not all ^{13}C NMR signals resolved). HRMS-ESI: calculated for $C_{54}H_{55}N_2O_4$ $[M+H]^+$ 795.4162, found 795.4161.

2,7-Bis(2-ethylhexyl)-4,9-bis((E)-4-methoxystyryl)benzo[lmn]- [3,8]phenanthroline-1,3,6,8(2H,7H)-tetraone (**4h**):

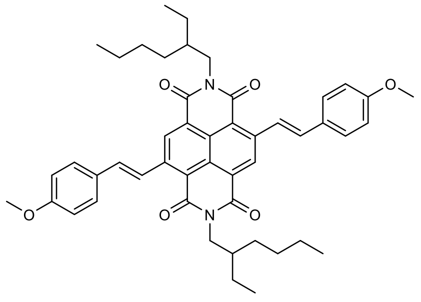

4h

The title compound was synthesised following general protocol 1 using 4-methoxystyrene. The crude mixture was purified by flash column chromatography (1:4 petroleum ether:dichloromethane) to give the product as a purple solid (0.064 g, 55% yield). ^{1}H NMR (600 MHz, $CDCl_3$) δ = 9.09 (s, 2H), 8.75 (d, 2H, J = 15.9 Hz), 7.67, (d, 4H, J = 8.4 Hz), 7.52 (d, 2H, J = 15.9 Hz), 6.98 (d, 4H, J= 8.4 Hz), 4.18 (m, 4H), 3.87 (s, 6H), 1.98 (m, 2H), 1.40 (m, 8H), 1.33 (m, 8H), 0.95 (t, 6H), 0.90 (t, 6H). ^{13}C NMR (151 MHz, $CDCl_3$) δ = 164.2, 163.2, 160.6, 142.4, 136.8, 131.2, 129.5, 129.2, 127.1, 125.1, 124.4, 119.7, 114.3, 55.4, 44.6, 37.8, 30.7, 28.6, 24.0, 23.1, 14.1, 10.7 ppm. HRMS-ESI: calculated for $C_{48}H_{55}N_2O_6$ $[M+H]^+$ 755.4060, found 755.4054.

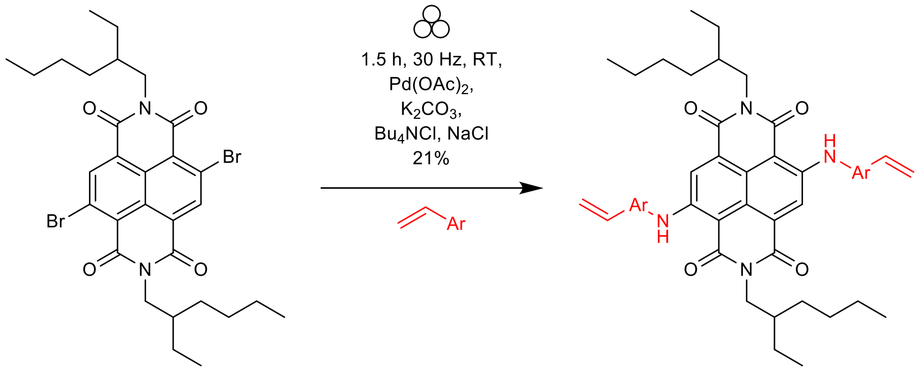

1 **4i**

Scheme S2: Synthesis of **4i**

<u>2,7-bis(2-ethylhexyl)-4,9-bis((4-vinylphenyl)amino)benzo[lmn][3,8]phenanthroline-1,3,6,8(2H,7H)-tetraone (**4i**):</u>

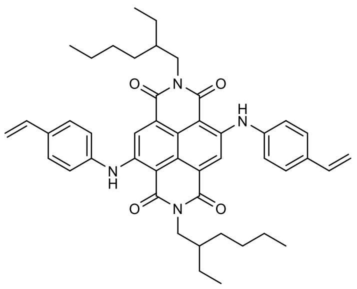

4i

The title compound was isolated from the crude reaction mixture after following general protocol 1 using 4-aminostyrene (54.5 µL, 0.465 mmol). 7 was isolated as a bright blue solid after column chromatography (0.023 g, 21 %), mp 228.4 – 231.2 °C. IR (neat) vmax/cm-1 2900 (N-H stretch), 1684 (C=C stretch), 1632 (C=O stretch), 1587 (N-H bend), 1200 (C-N stretch), 791 (C-H bend). ^{1}H NMR (600 MHz, $CDCl_3$) δ = 11.12 (s, 2H), 8.56 (s, 2H), 7.49 (d, 4H, J = 7.5 Hz), 7.32 (d, 4H, J = 7.5 Hz), 6.74 (dd, 2H, J = 17.5 Hz, 11.0 Hz), 5.77 (d, 2H, J = 17.5 Hz), 5.29 (d, 2H, J = 11.0 Hz), 4.10 (m, 4H), 1.92 (m, 2H), 1.39 – 1.25 (m, 16H), 0.91 (t, 6H), 0.87 (t, 6H). ^{13}C NMR (151 MHz, $CDCl_3$) δ = 166.6, 163.1, 146.9, 138.1, 136.0, 135.1, 127.7, 125.7, 123.9, 122.2, 120.6, 113.8, 103.3, 44.3, 37.8, 30.7, 28.7, 24.0, 23.1, 14.1, 10.7. HRMS-ESI: calculated for $C_{46}H_{53}N_4O_4$ $[M+H]^+$ 725.4067 found 725.4073.

S2.0 Characterization Data

N,N’-bis(2-ethylhexyl)-2,6-dibromo-1,4,5,8-naphthalenetetracarboxylic acid, **1**:

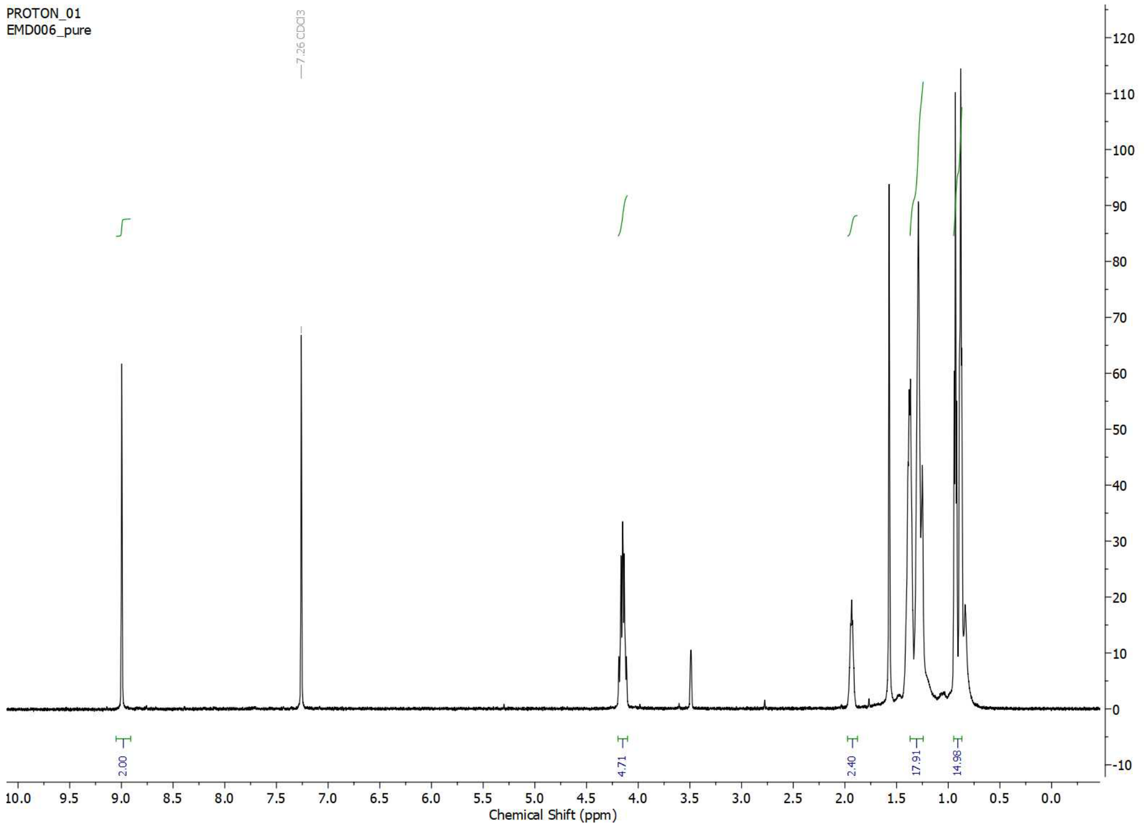

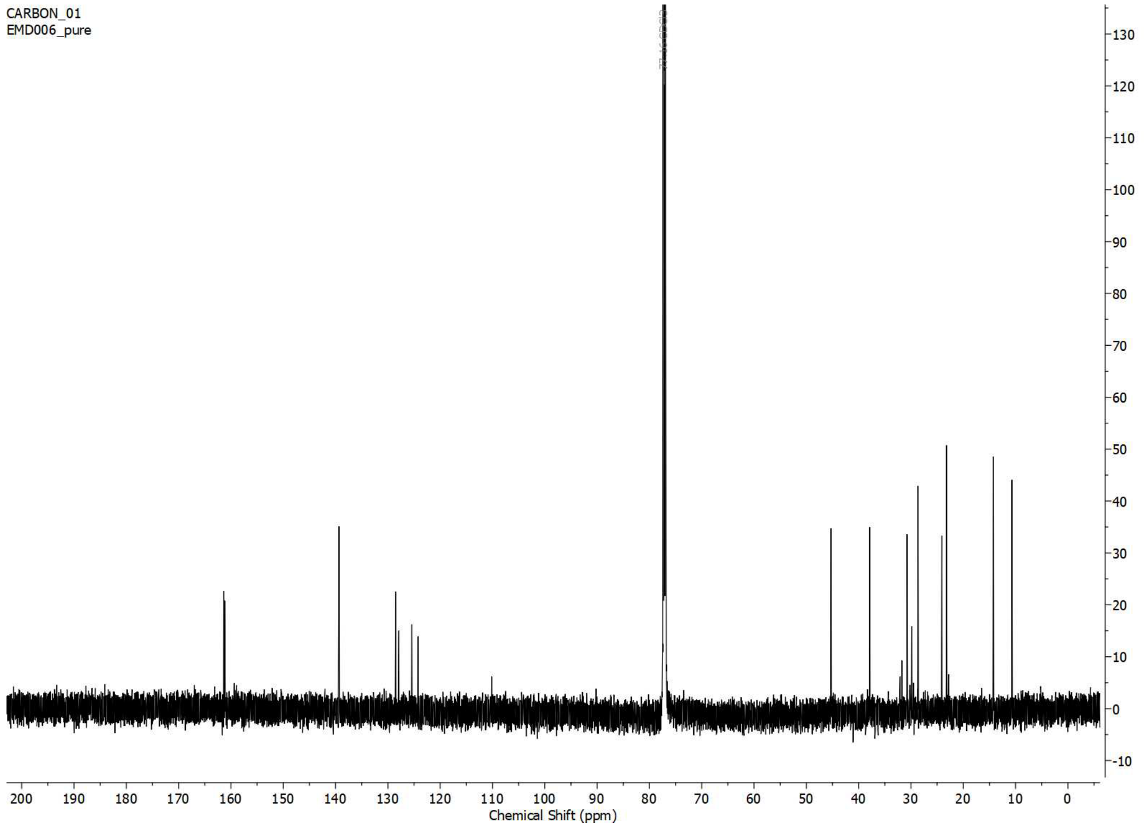

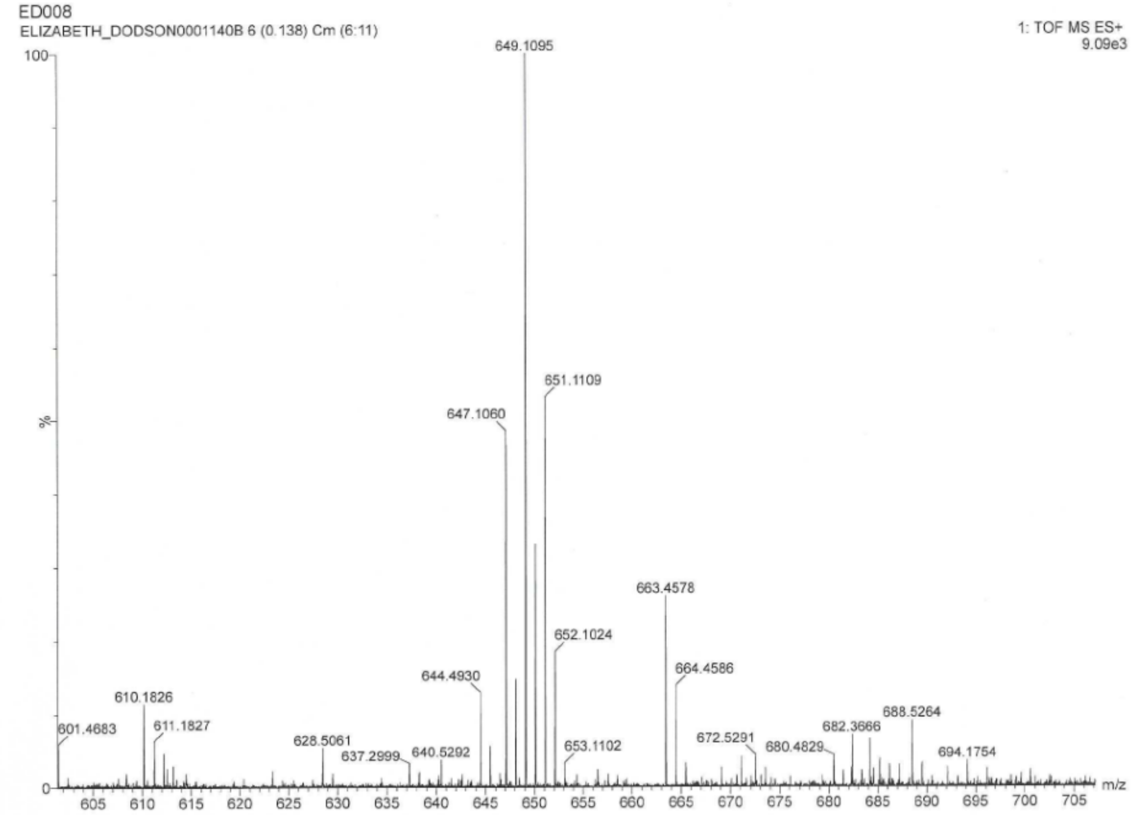

Elemental Composition Report

Page 1

Single Mass Analysis

Tolerance = 100.0 PPM / DBE: min = -50.0, max = 100.0
Element prediction: Off
Number of isotope peaks used for i-FIT = 3

Monoisotopic Mass, Even Electron Ions
2 formula(e) evaluated with 1 results within limits (up to 50 closest results for each mass)
Elements Used:
C: 30-30 H: 0-100 N: 2-2 O: 4-4 P: 0-1 Br: 2-2

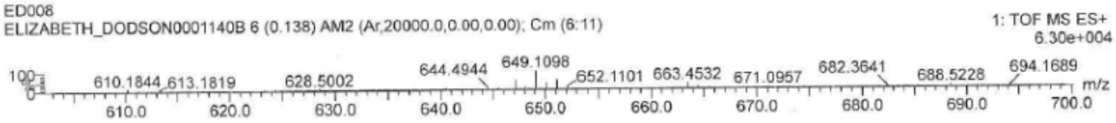

Mass	Calc. Mass	mDa	PPM	DBE	i-FIT	Norm	Conf(%)	Formula
Minimum:				-50.0				
Maximum:		5.0	100.0	100.0				
647.1118	647.1120	-0.2	-0.3	12.5	294.8	n/a	n/a	C30 H37 N2 O4 Br2

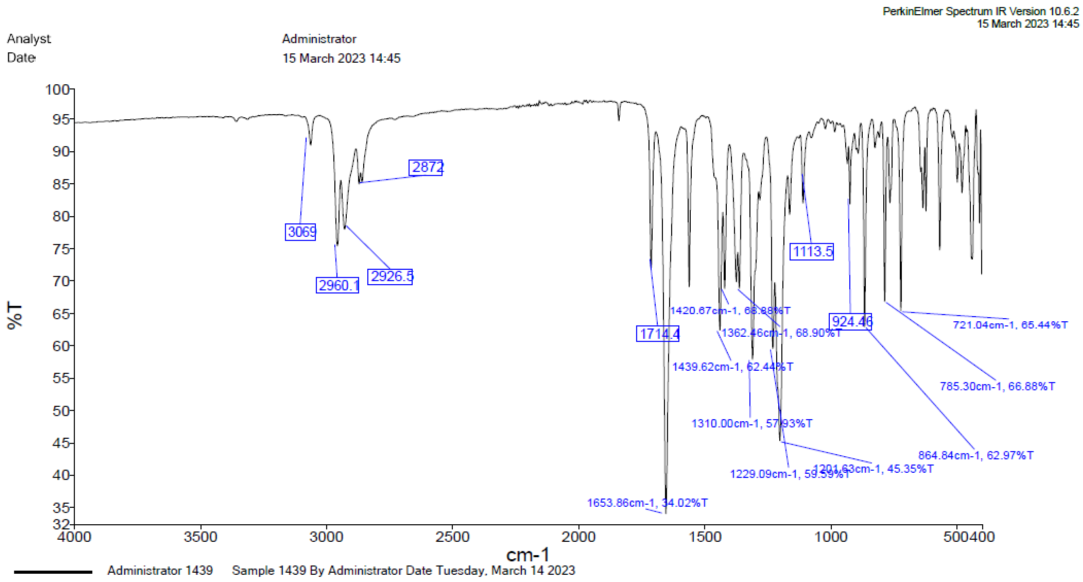

Figure S3: ^{1}H (600 MHz) and ^{13}C (150 MHz) NMR spectra (chloroform-*d* at 298K), mass spectrum, elemental composition, and IR spectrum of **1**.

2,7-Bis(2-ethylhexyl)-4,9-di((E)-styryl)benzo[lmn][3,8]- phenanthroline-1,3,6,8-(2H,7H)-tetraone (**4a**):

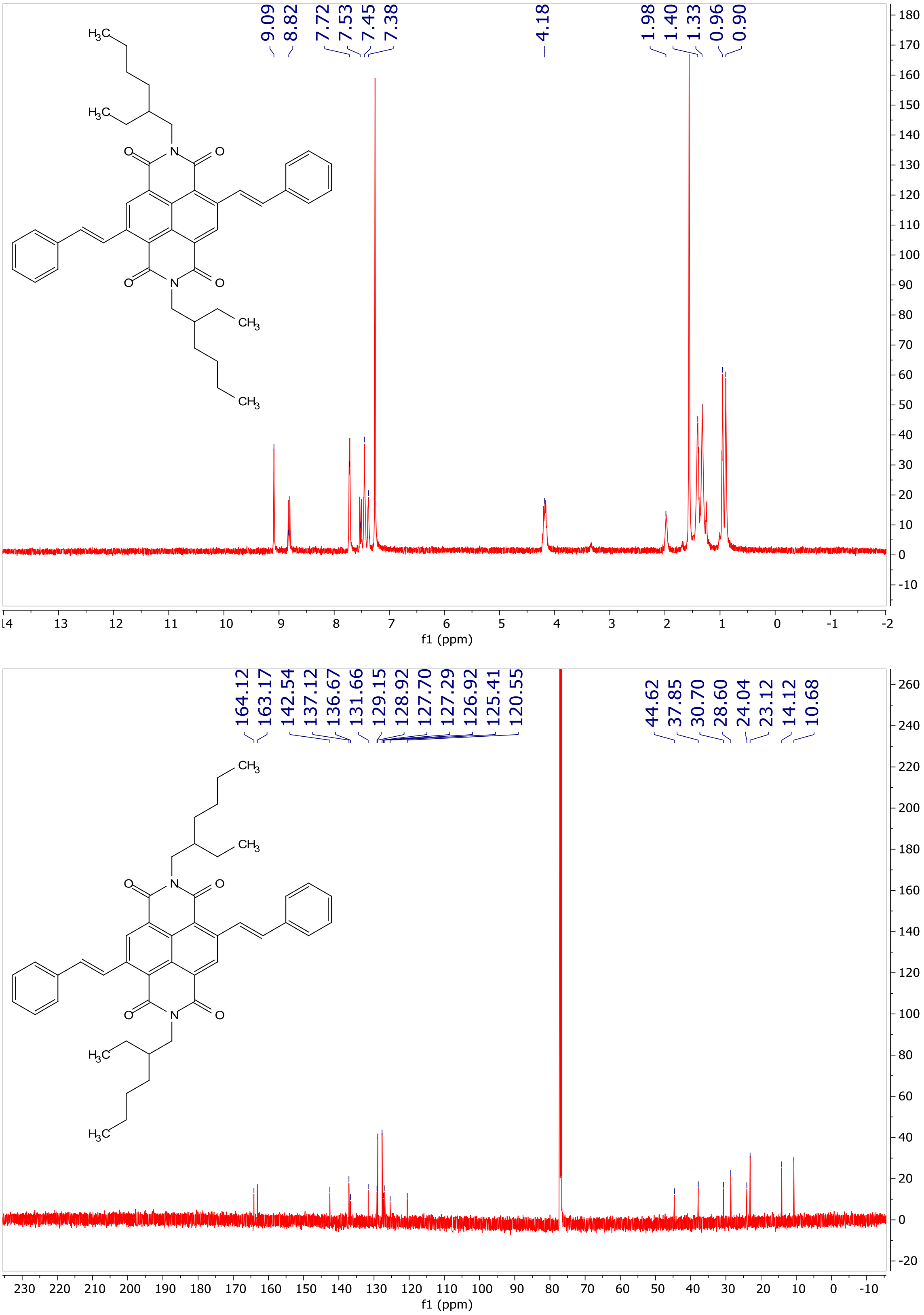

Figure S4: ^{1}H (600 MHz) and ^{13}C (150 MHz) NMR spectra (chloroform-*d* at 298K) of **4a**.

2,7-Bis(2-ethylhexyl)-4,9-bis((E)-4-nitrostyryl)benzo[lmn][3,8]- phenanthroline-1,3,6,8(2H,7H)-tetraone (**4b**):

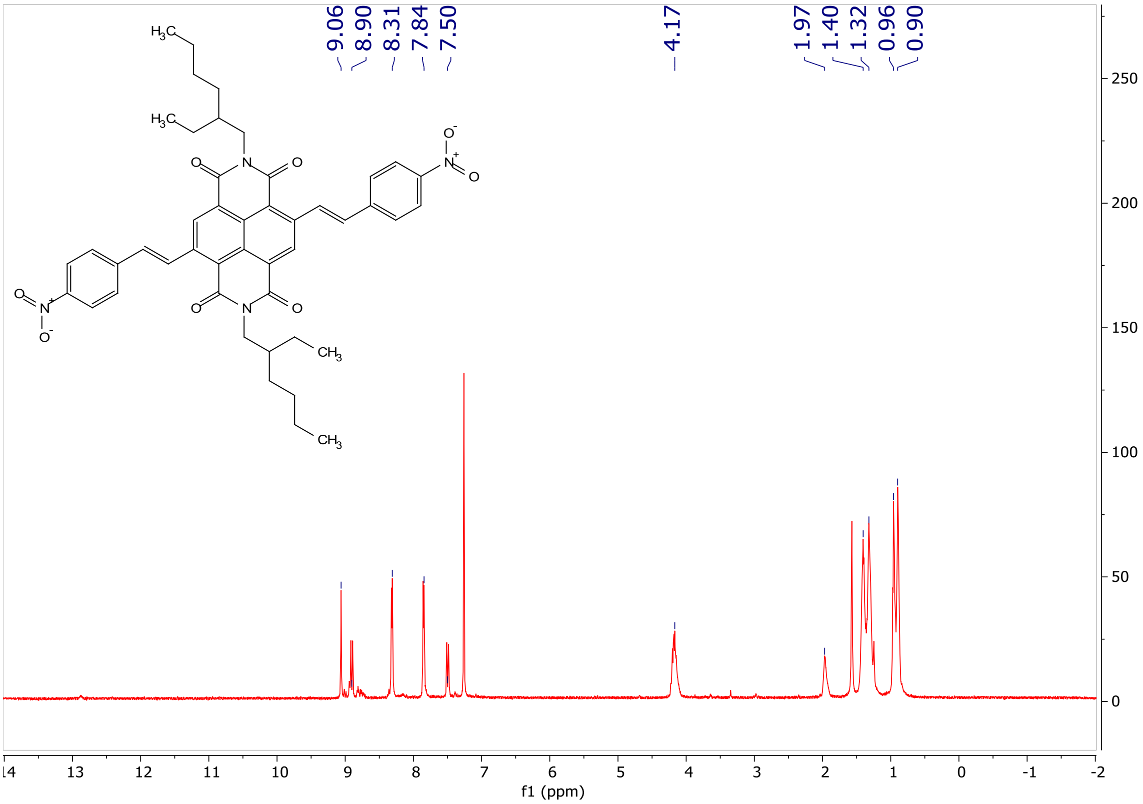

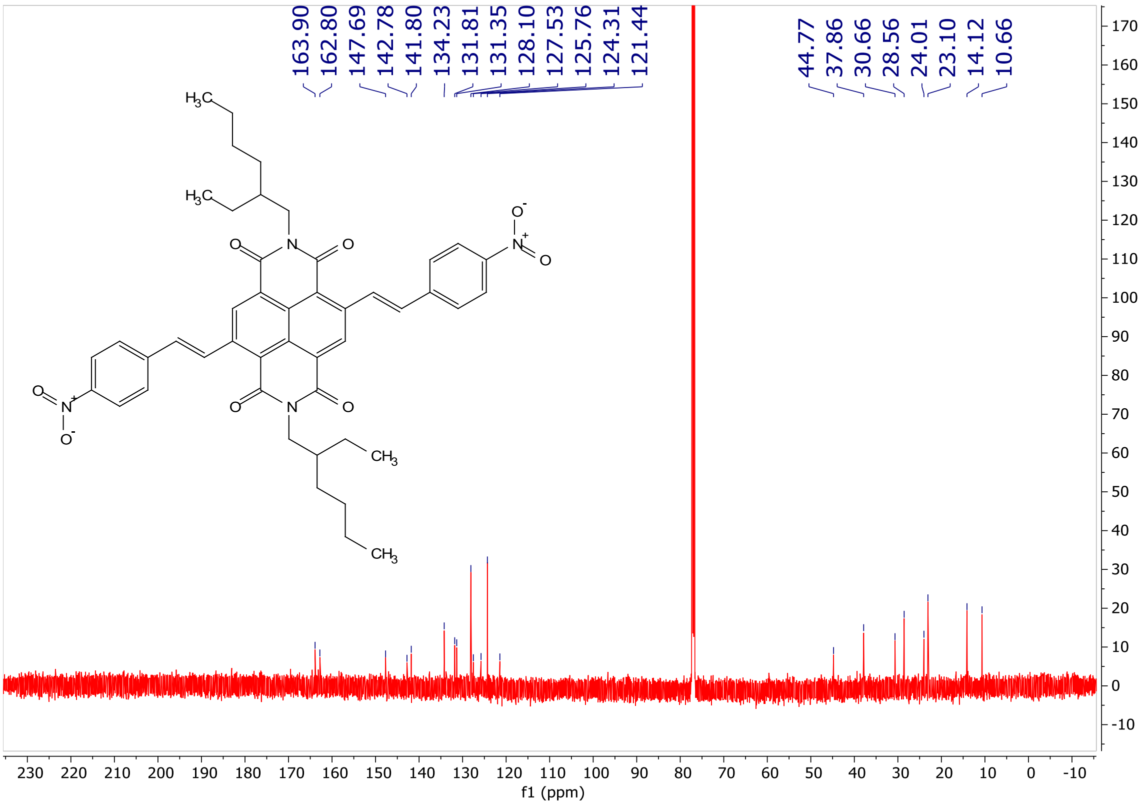

Figure S5: ^{1}H (600 MHz) and ^{13}C (150 MHz) NMR spectra (chloroform-*d* at 298K) of **4b**.

2,7-bis(2-ethylhexyl)-4,9-bis((E)-4-fluorostyryl)benzo[lmn][3,8]phenanthroline-1,3,6,8(2H,7H)-tetraone (**4c**):

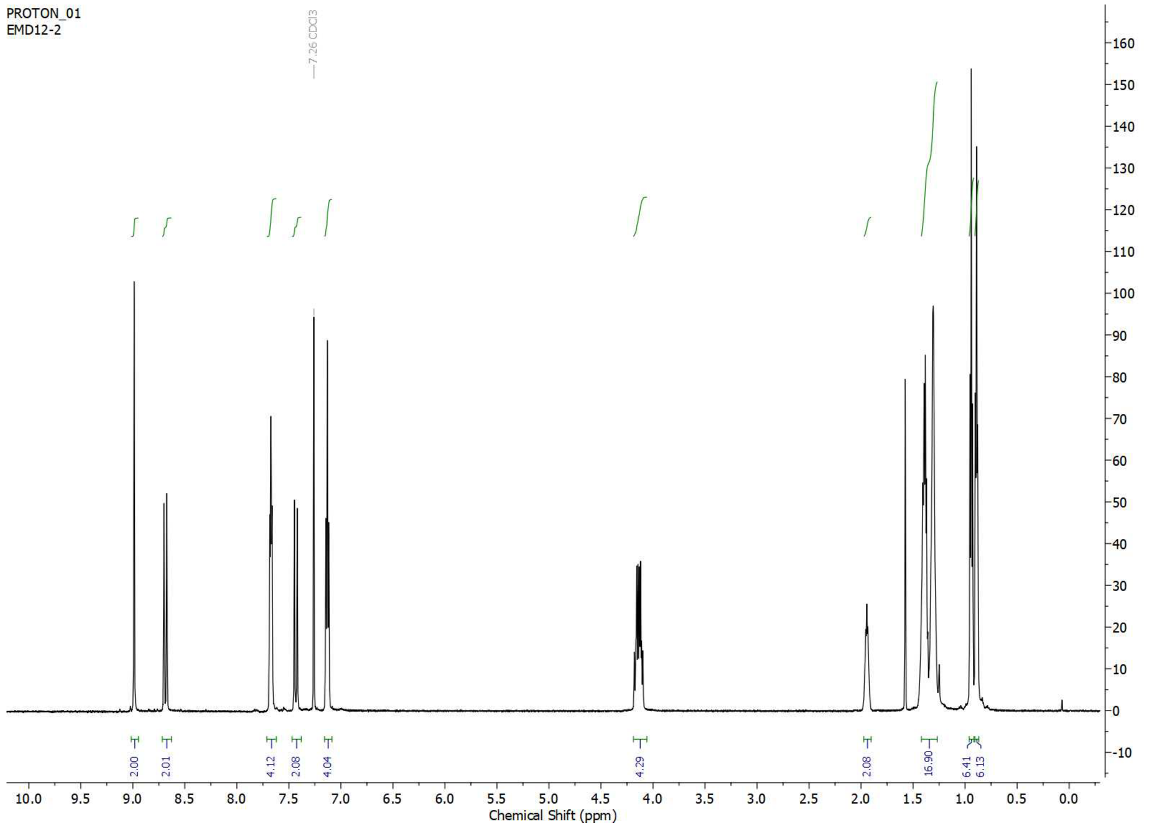

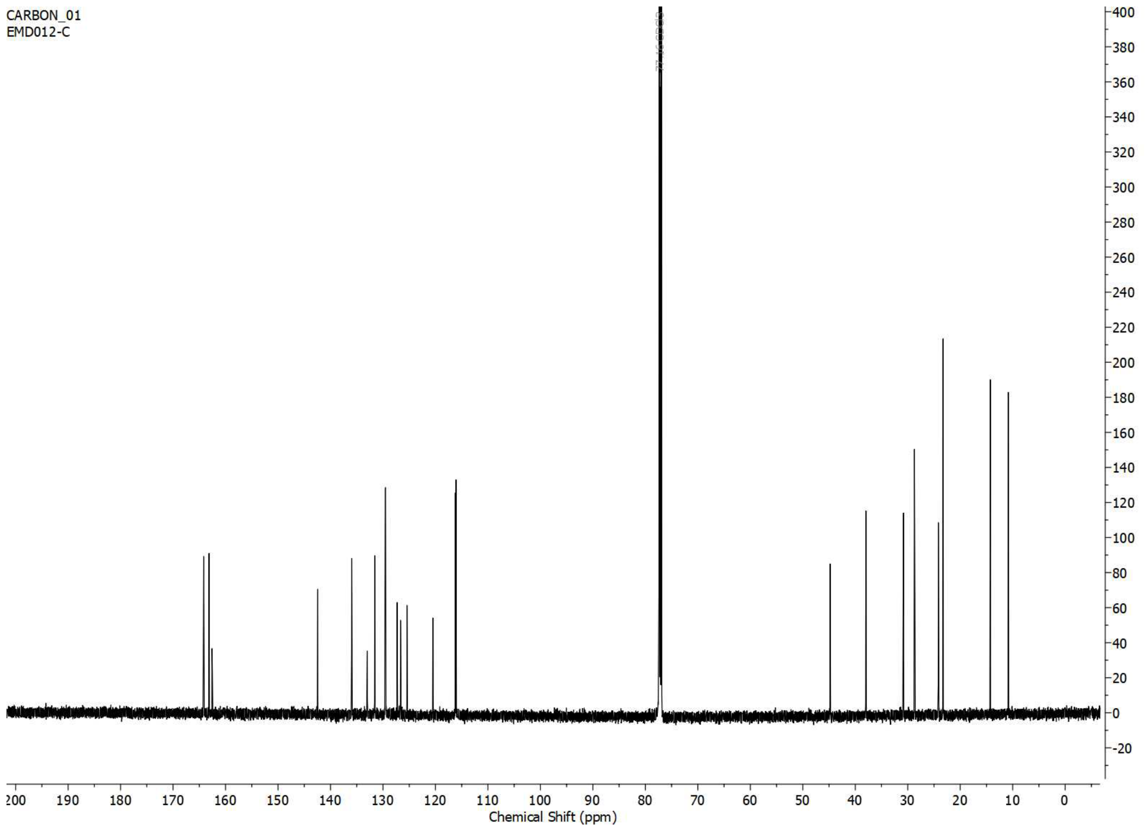

Single Mass Analysis
Tolerance = 250.0 mDa / DBE: min = -1.5, max = 50.0
Element prediction: Off
Number of isotope peaks used for i-FIT = 3
Monoisotopic Mass, Even Electron Ions
28 formula(e) evaluated with 1 results within limits (up to 50 closest results for each mass)
Elements Used:

Mass	Calc. Mass	mDa	PPM	DBE	Formula	i-FIT	i-FIT Norm	Fit Conf %	C	H	N	O	F
731.3624	731.3660	-3.6	-4.9	22.5	C46 H49 N2 O4 F2	671.9	n/a	n/a	46	49	2	4	2

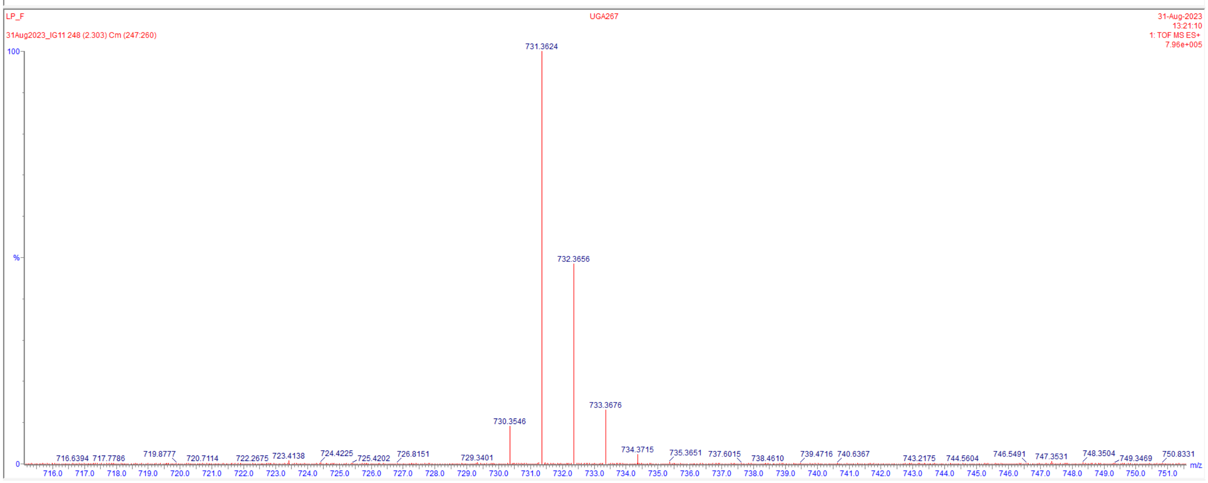

Figure S6: ^{1}H (600 MHz) and ^{13}C (150 MHz) NMR spectra (chloroform-*d* at 298K), mass spectrum, and elemental composition of **4c**.

2,7-bis(2-ethylhexyl)-4,9-bis((E)-4-chlorostyryl)benzo[lmn][3,8]phenanthroline-1,3,6,8(2H,7H)-tetraone (**4d**):

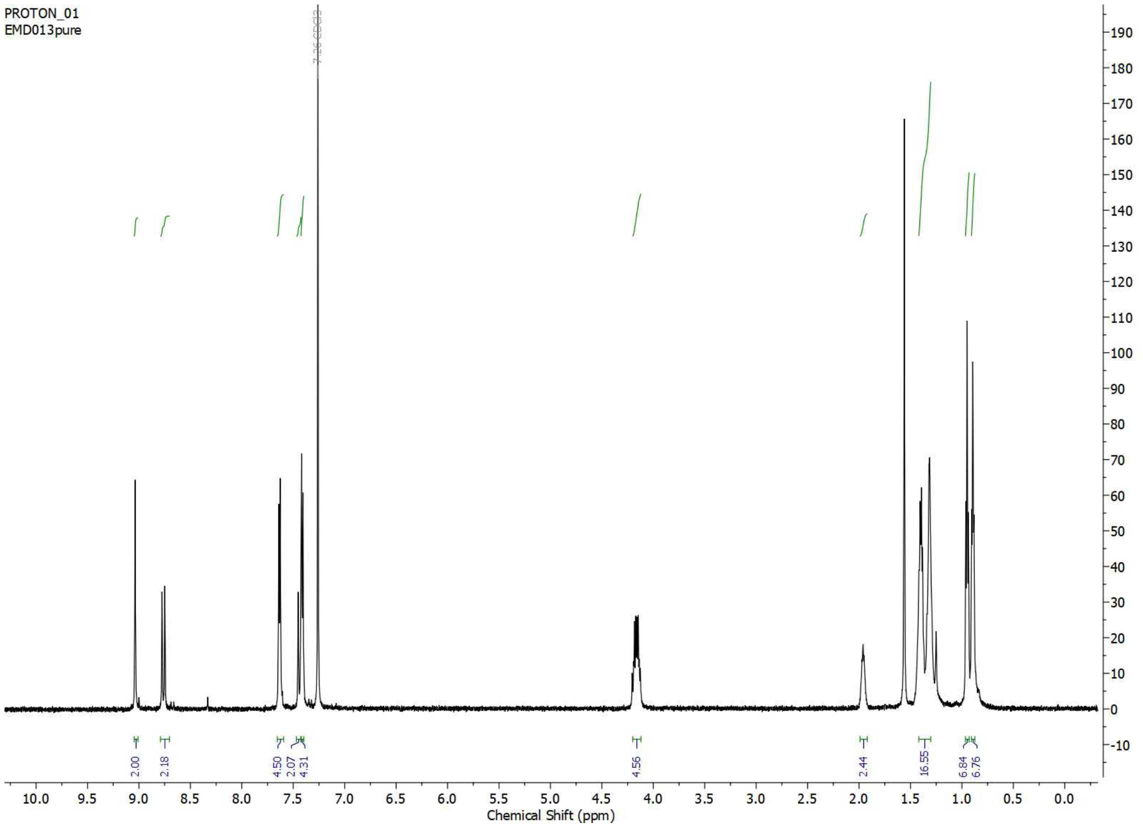

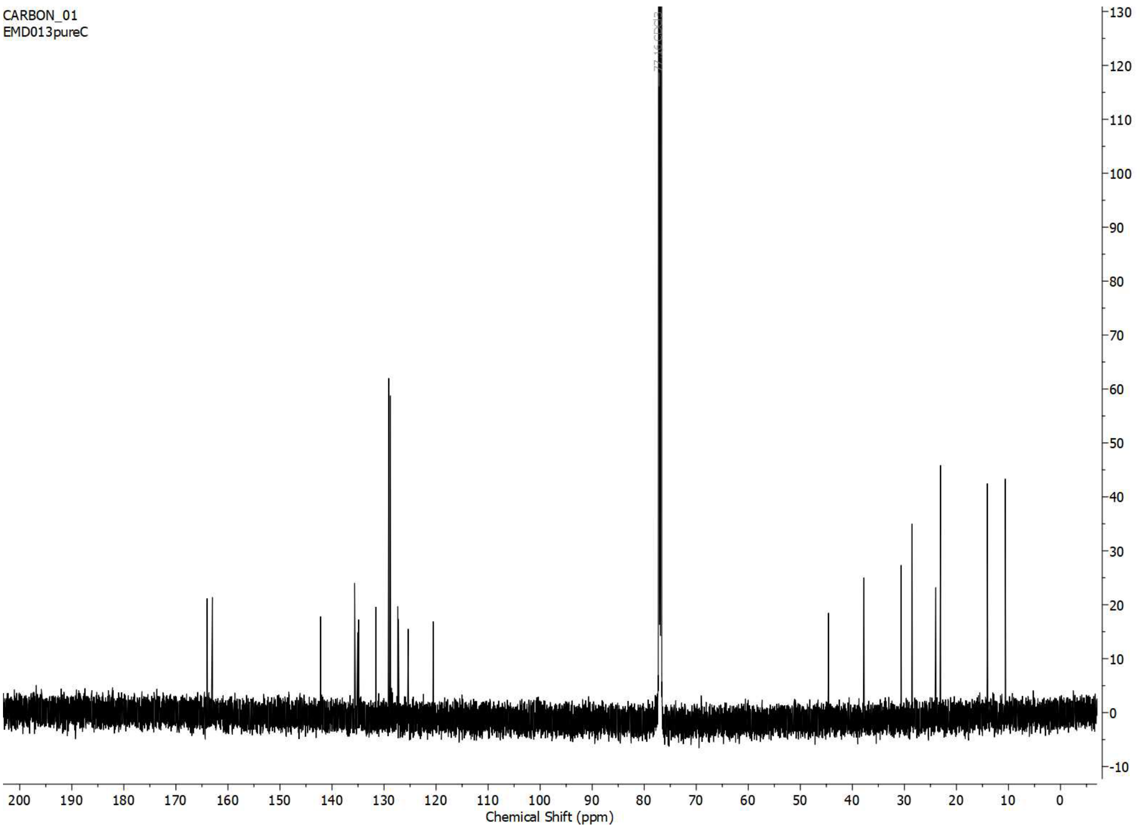

Single Mass Analysis
Tolerance = 250.0 mDa / DBE: min = -1.5, max = 50.0
Element prediction: Off
Number of isotope peaks used for i-FIT = 3
Monoisotopic Mass, Even Electron Ions
34 formula(e) evaluated with 1 results within limits (up to 50 closest results for each mass)
Elements Used:

Mass	Calc. Mass	mDa	PPM	DBE	Formula	i-FIT	i-FIT Norm	Fit Conf %	C	H	N	O	Cl
763.3033	763.3069	-3.6	-4.7	22.5	C46 H49 N2 O4 Cl2	834.9	n/a	n/a	46	49	2	4	2

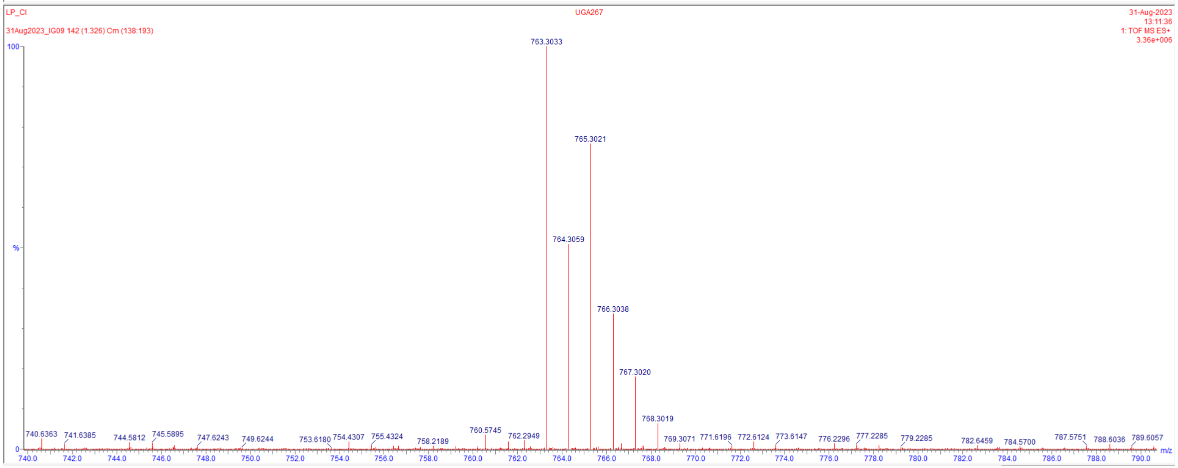

Figure S7: ^{1}H (600 MHz) and ^{13}C (150 MHz) NMR spectra (chloroform-*d* at 298K), mass spectrum, and elemental composition of **4d**.

Synthesis of 2,7-bis(2-ethylhexyl)-4,9-bis((E)-4-(trifluoromethyl)styryl)benzo[lmn][3,8]phenanthroline-1,3,6,8(2H,7H)-tetraone (**4e**):

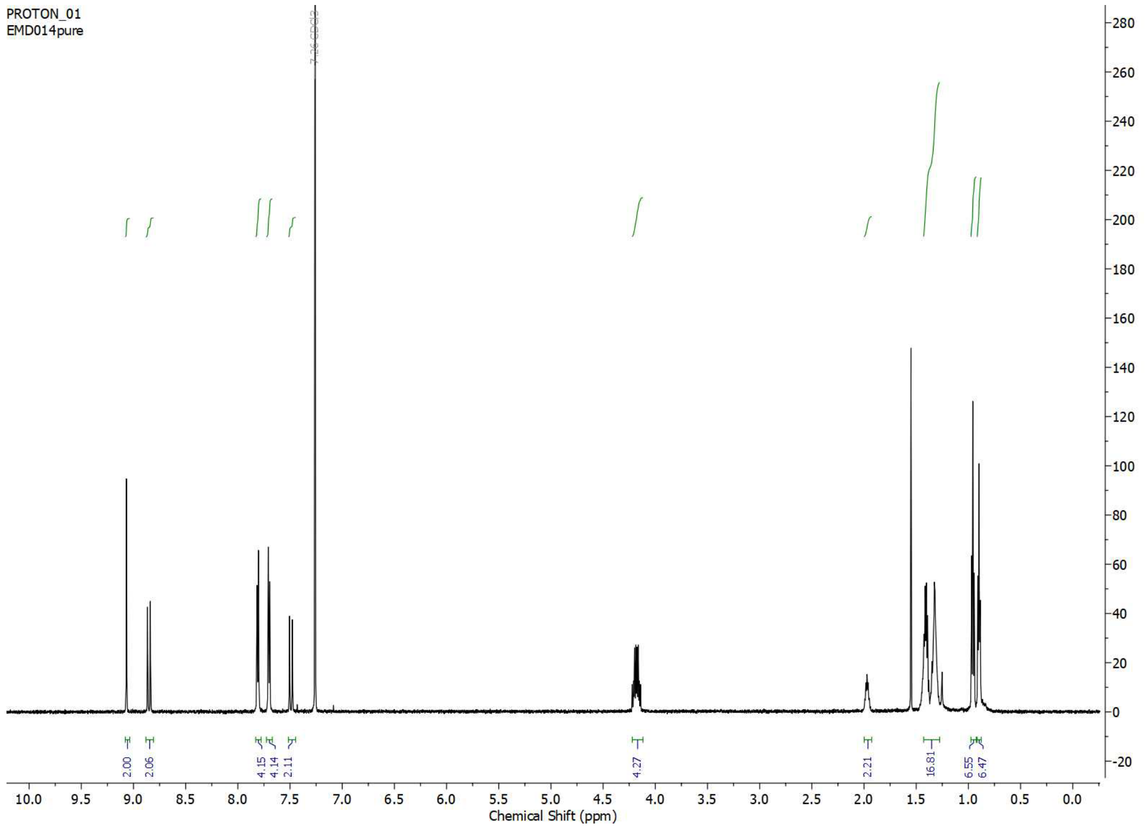

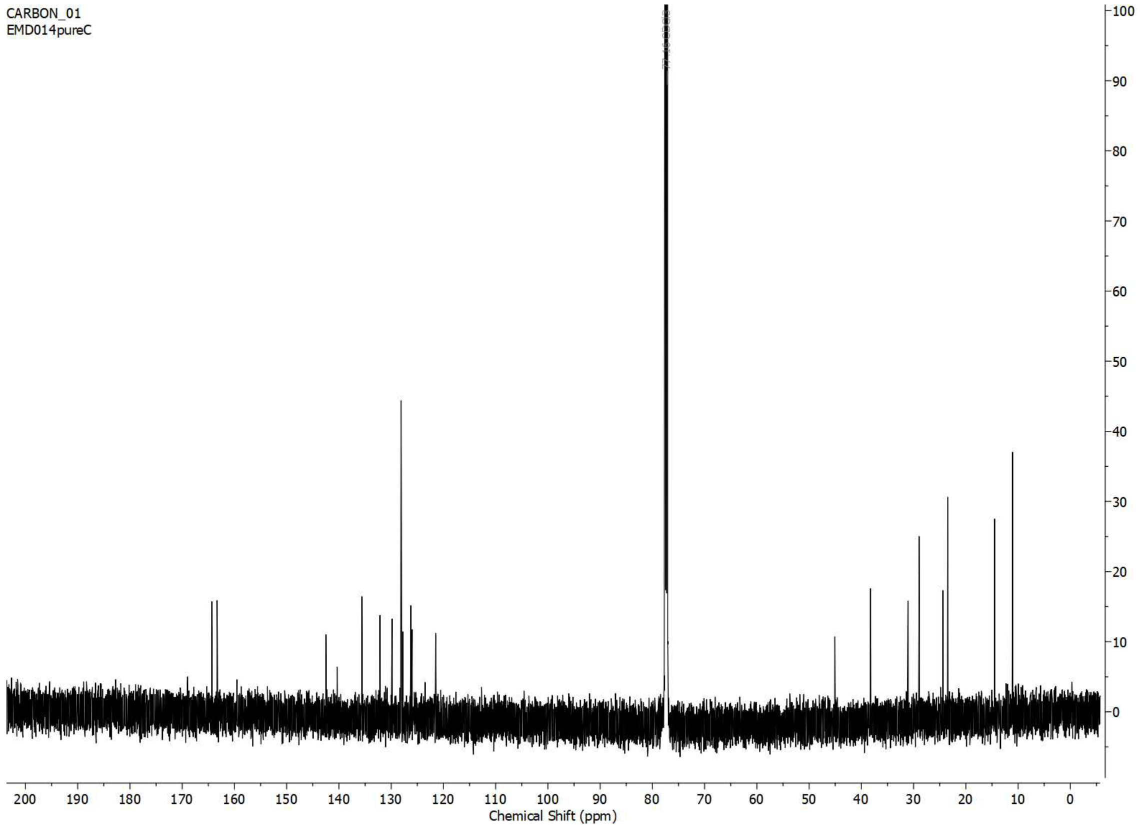

Elemental Composition

File Edit View Process Help

Single Mass Analysis

Tolerance = 3.0 mDa / DBE: min = -1.5, max = 50.0

Element prediction: Off

Number of isotope peaks used for i-FIT = 3

Monoisotopic Mass, Even Electron Ions

361 formula(e) evaluated with 1 results within limits (up to 50 closest results for each mass)

Elements Used:

Mass	Calc. Mass	mDa	PPM	DBE	Formula	i-FIT	i-FIT Norm	Fit Conf %	C	H	N	O	F
831.3568	831.3597	-2.9	-3.5	22.5	C48 H49 N2 O4 F6	741.7	n/a	n/a	48	49	2	4	6

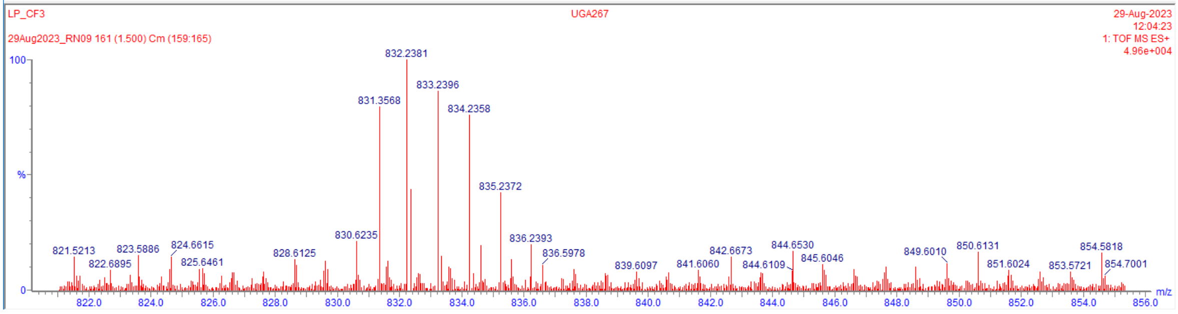

Elemental Composition

File Edit View Process Help

Single Mass Analysis

Tolerance = 5.0 mDa / DBE: min = -1.5, max = 50.0

Element prediction: Off

Number of isotope peaks used for i-FIT = 3

Monoisotopic Mass, Even Electron Ions

103 formula(e) evaluated with 1 results within limits (up to 50 closest results for each mass)

Elements Used:

Mass	Calc. Mass	mDa	PPM	DBE	Formula	i-FIT	i-FIT Norm	Fit Conf %	C	H	N	O
723.4150	723.4162	-1.2	-1.7	22.5	C48 H55 N2 O4	781.8	n/a	n/a	48	55	2	4

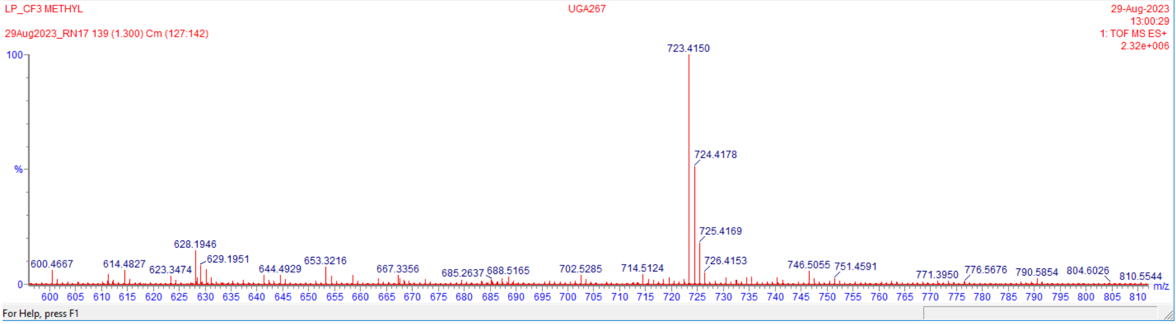

For Help, press F1

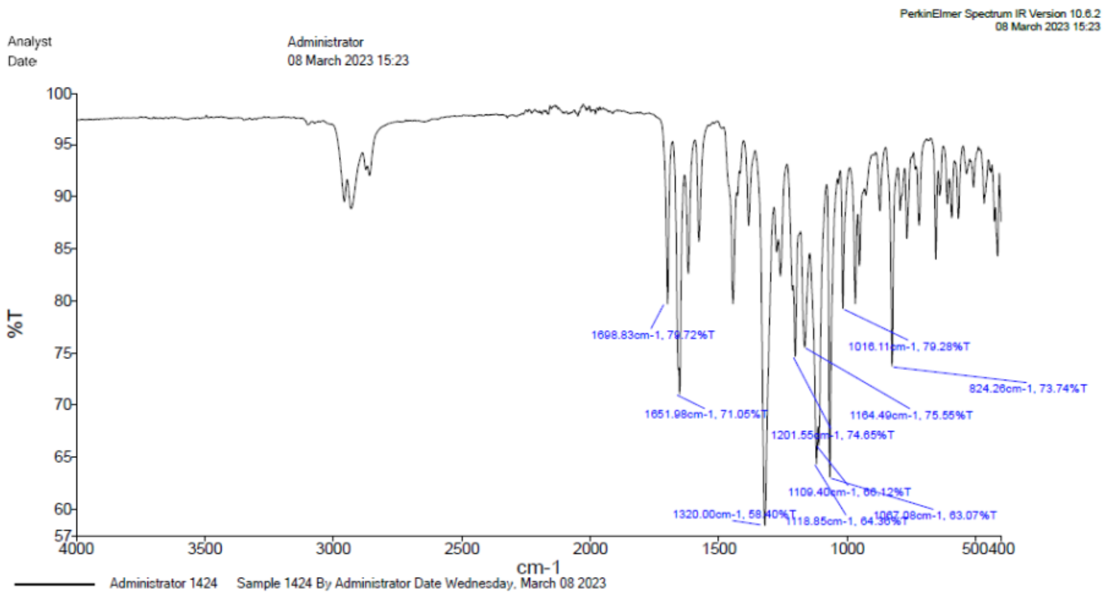

Figure S8: ^{1}H (600 MHz) and ^{13}C (150 MHz) NMR spectra (chloroform-*d* at 298K), mass spectrum, elemental composition, and IR spectrum of **4e**.

2,7-Bis(2-ethylhexyl)-4,9-bis((E)-2-methylstyryl)benzo[lmn][3,8]- phenanthroline-1,3,6,8(2H,7H)-tetraone (**4f**):

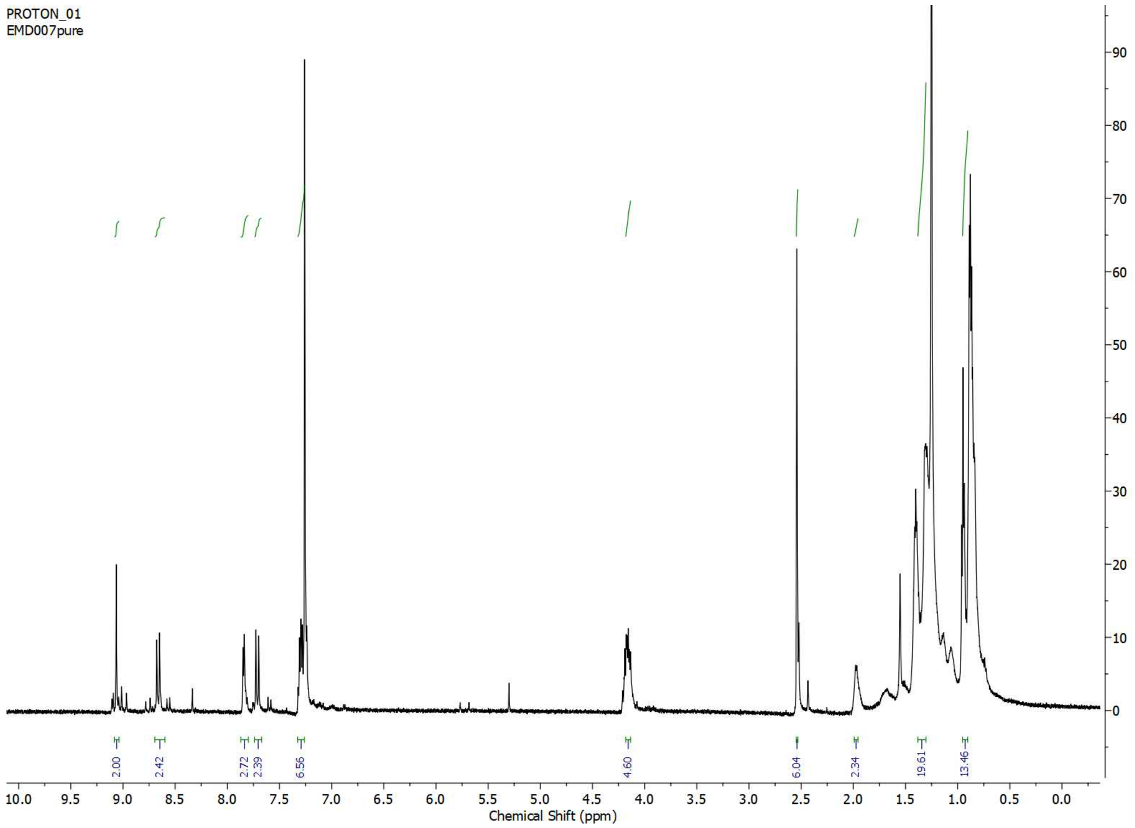

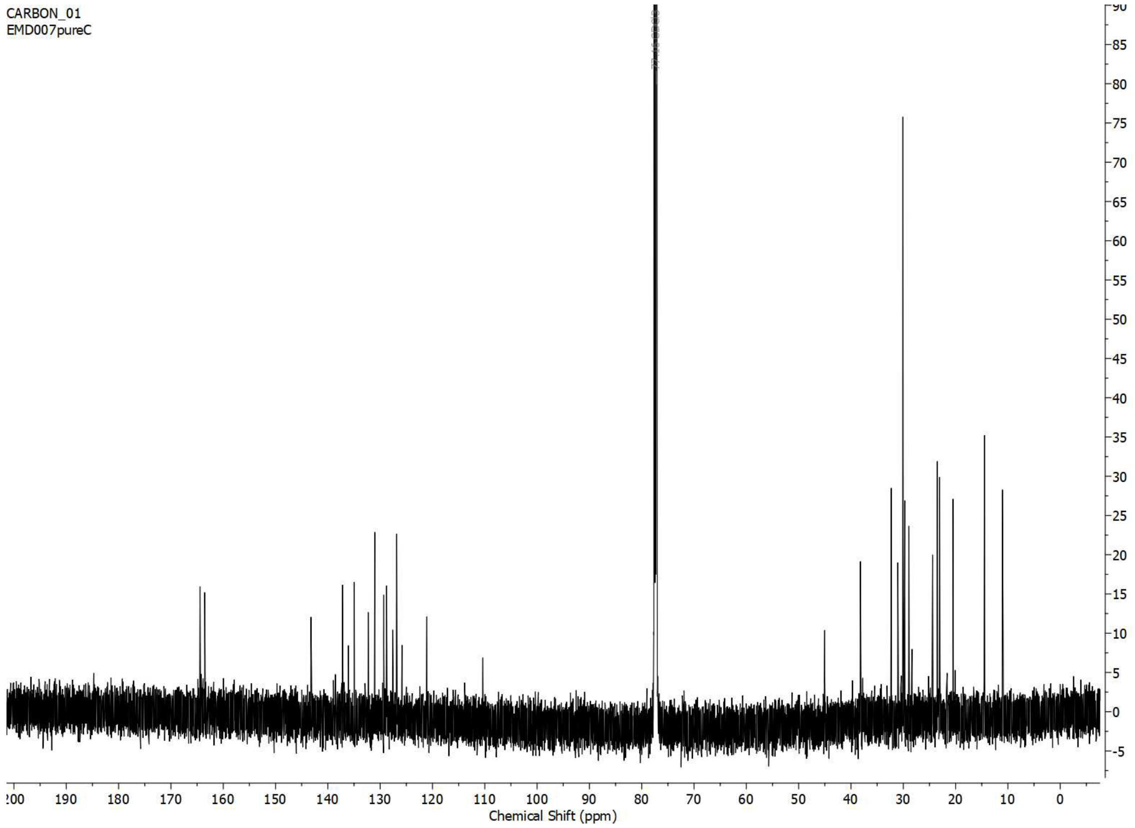

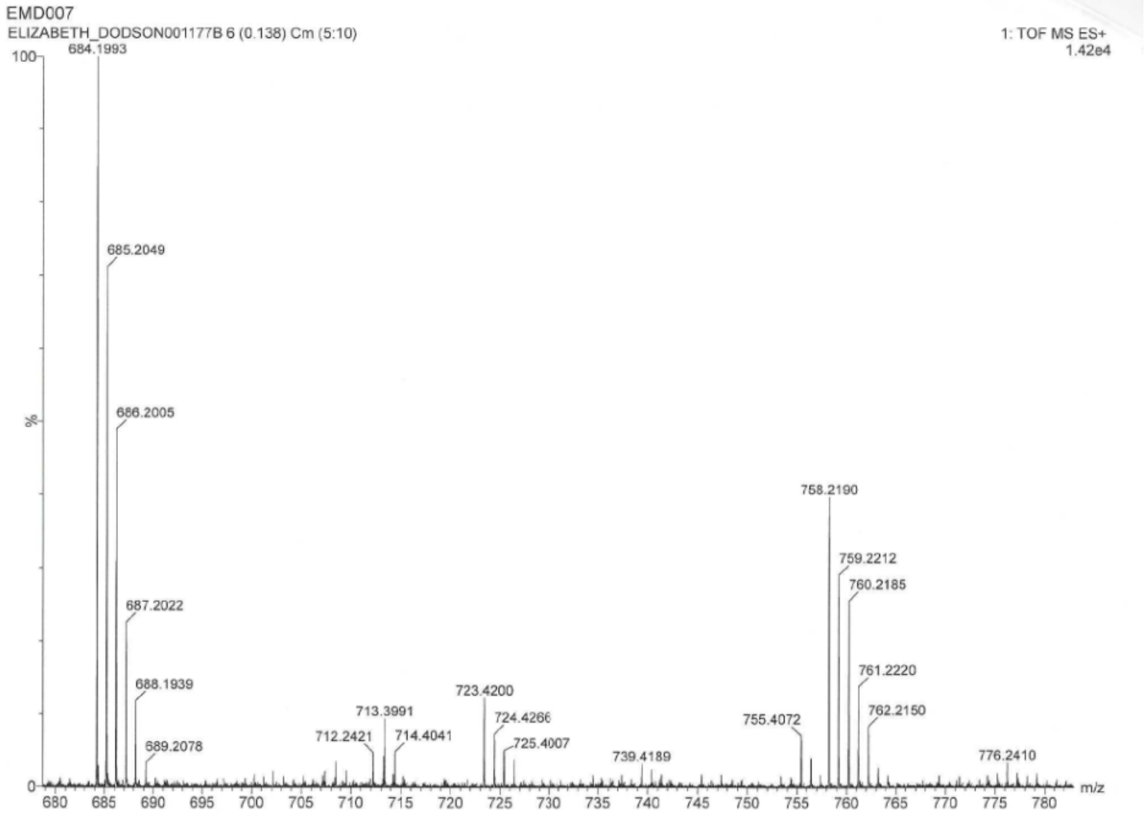

[illegible]ital Composition Report

Page 1

[illegible]gle Mass Analysis

[illegible]erance = 100.0 PPM / DBE: min = -50.0, max = 100.0
[illegible]ement prediction: Off
[illegible]umber of isotope peaks used for i-FIT = 3

Monoisotopic Mass, Odd and Even Electron Ions
2 formula(e) evaluated with 1 results within limits (up to 50 closest results for each mass)
Elements Used:
C: 48-48 H: 0-100 N: 2-2 O: 4-4 P: 0-1
EMD007
ELIZABETH_DODSON001177B 6 (0.138) AM2 (Ar,20000.0,0.00,0.00); Cm (5:10)

1: TOF MS ES+
1.50e+004

Minimum:				-50.0				
Maximum:		5.0	100.0	100.0				

Mass	Calc. Mass	mDa	PPM	DBE	i-FIT	Norm	Conf(%)	Formula
723.4149	723.4162	-1.3	-1.8	22.5	325.8	n/a	n/a	C48 H55 N2 O4

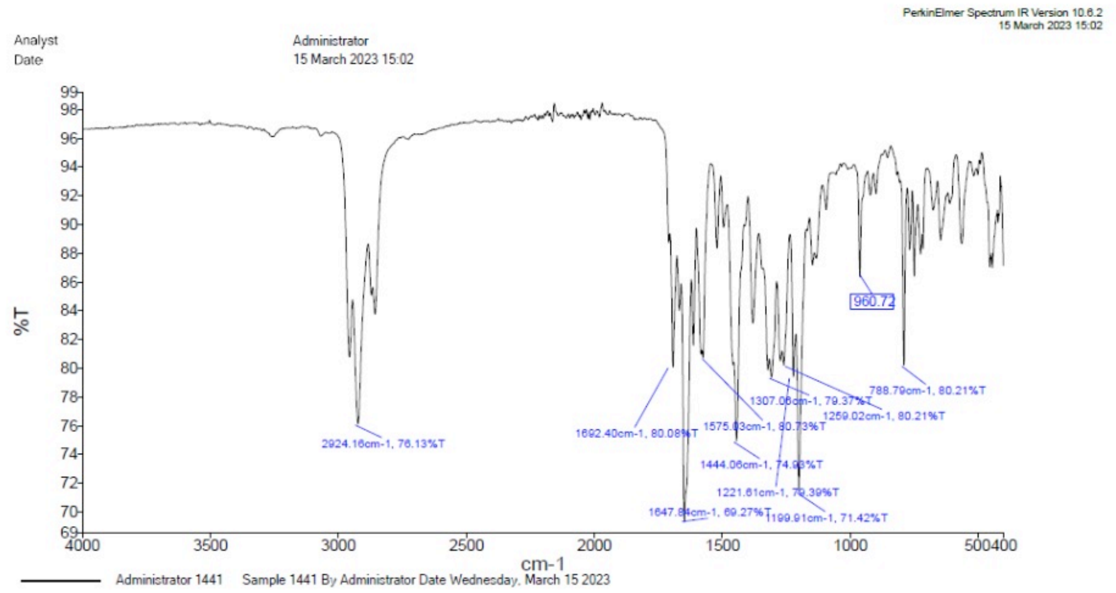

Figure S9: ^{1}H (600 MHz) and ^{13}C (150 MHz) NMR spectra (chloroform-*d* at 298K), mass spectrum, elemental composition, and IR spectrum of **4f**.

2,7-Bis(2-ethylhexyl)-4,9-bis((E)-2-naphthalen-2-yl)vinyl)benzo- [lmn][3,8]phenanthroline-1,3,6,8-(2H,7H)-tetraone (**4g**):

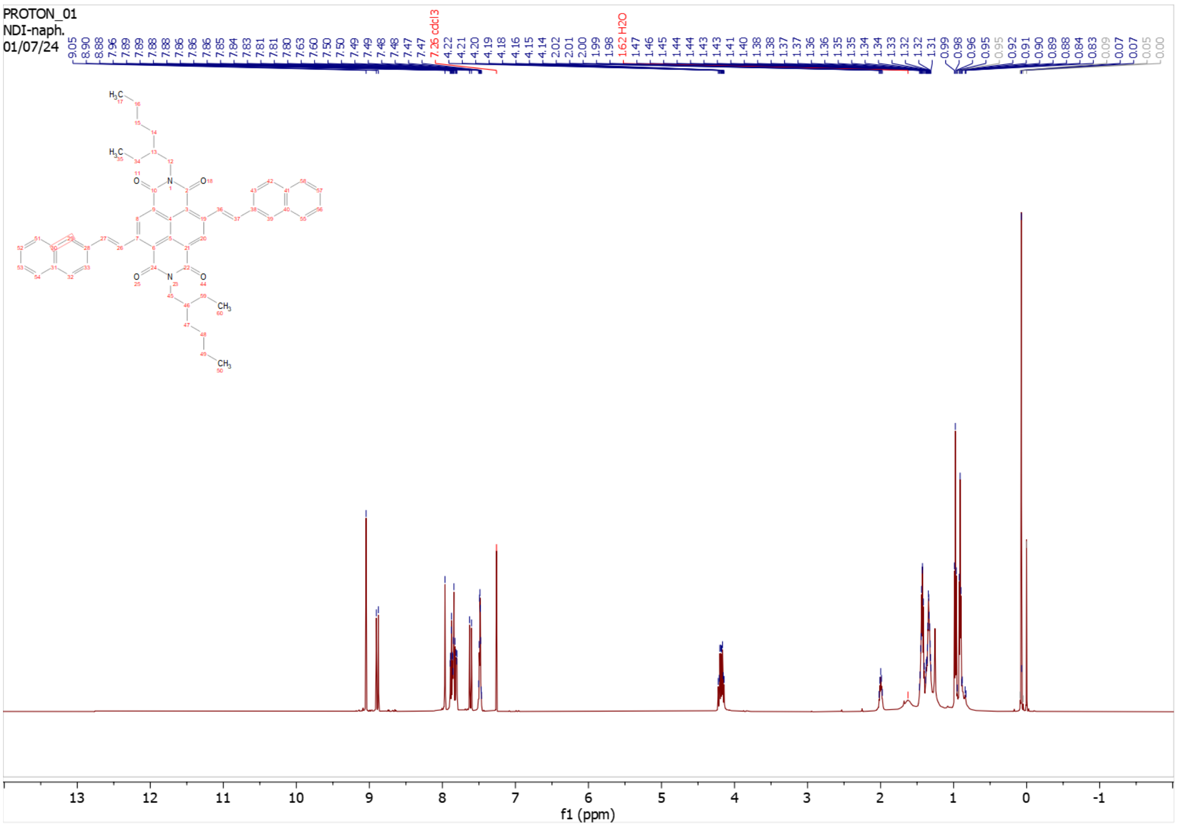

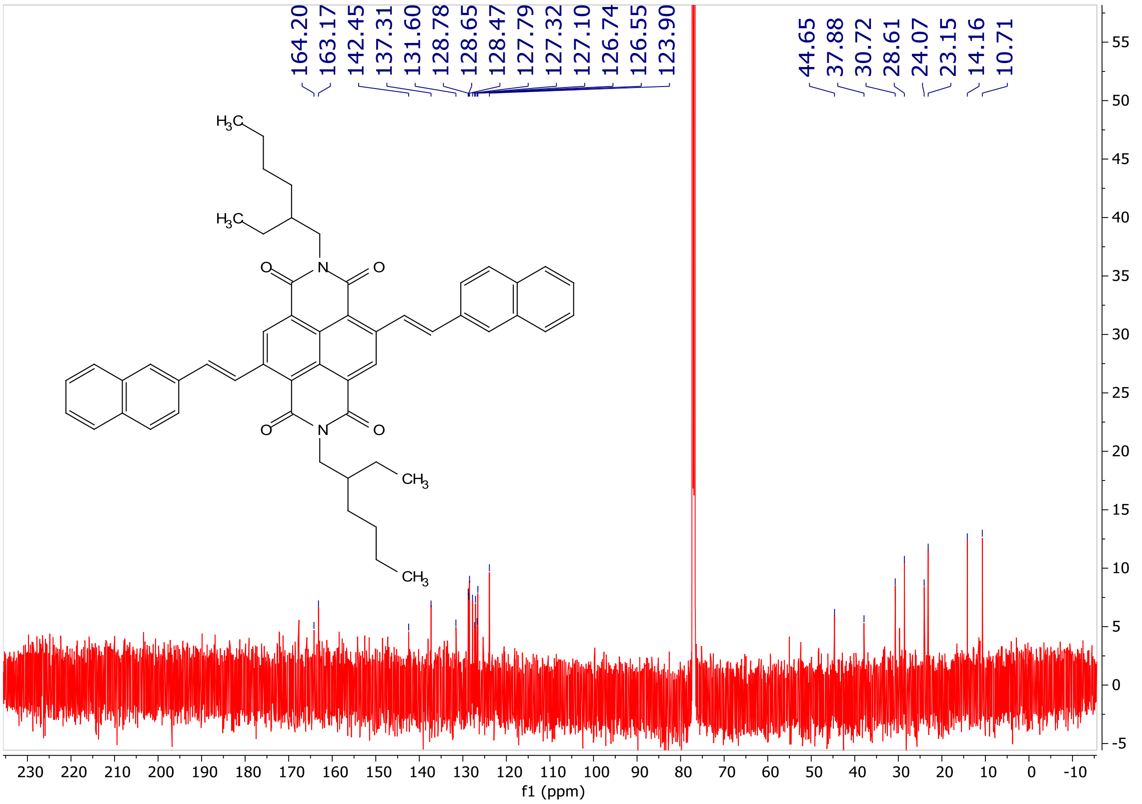

Figure S10: ^{1}H (600 MHz) and ^{13}C (150 MHz) NMR spectra (chloroform-*d* at 298K) of **4g**.

2,7-Bis(2-ethylhexyl)-4,9-bis((E)-4-methoxystyryl)benzo[lmn]- [3,8]phenanthroline-1,3,6,8(2H,7H)-tetraone (**4h**):

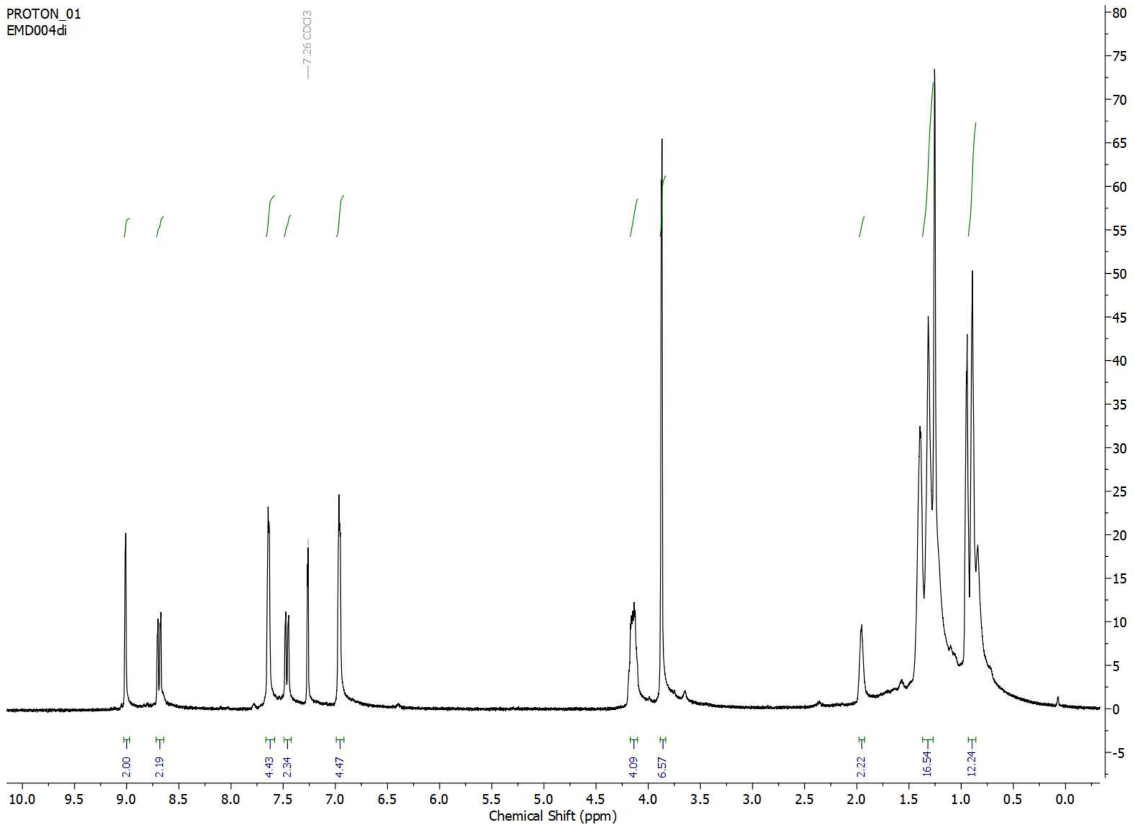

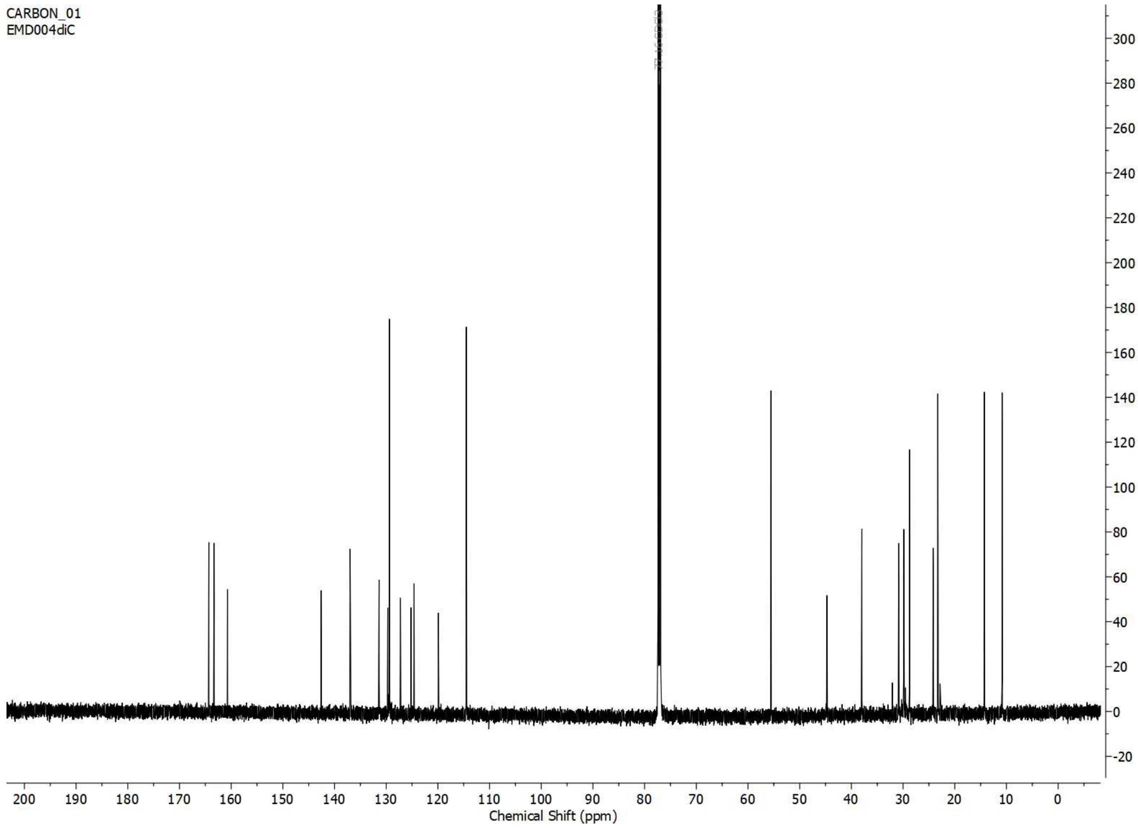

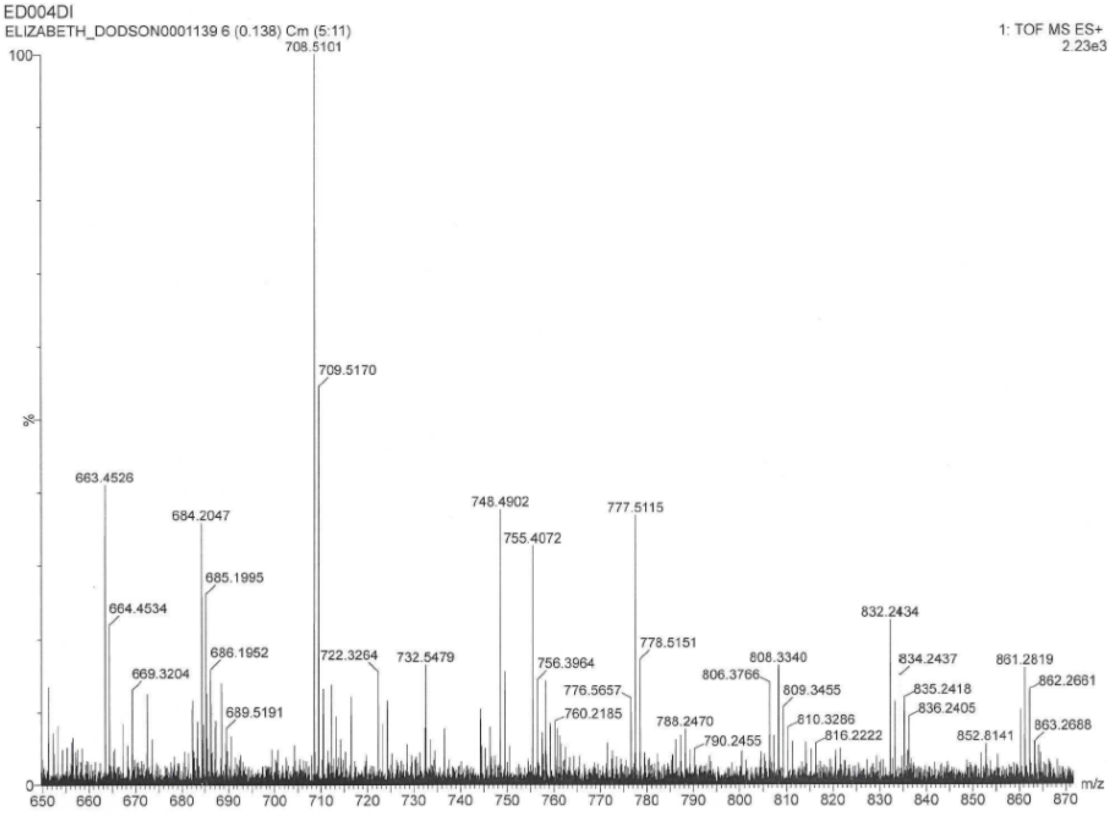

iental Composition Report **Page 1**

ingle Mass Analysis
Tolerance = 100.0 PPM / DBE: min = -50.0, max = 100.0
Element prediction: Off
Number of isotope peaks used for i-FIT = 3

Monoisotopic Mass, Even Electron Ions
2 formula(e) evaluated with 1 results within limits (up to 50 closest results for each mass)
Elements Used:
C: 48-48 H: 0-100 N: 2-2 O: 6-6 P: 0-1
ED004DI
ELIZABETH_DODSON0001139 6 (0.138) AM2 (Ar,20000.0,0.00,0.00); Cm (5:11) 1: TOF MS ES+ 1.63e+004

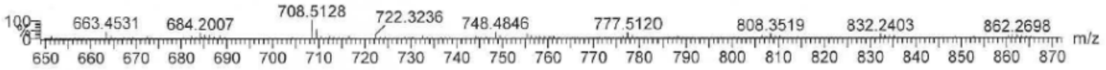

Minimum:				-50.0				
Maximum:		5.0	100.0	100.0				
Mass	Calc. Mass	mDa	PPM	DBE	i-FIT	Norm	Conf(%)	Formula
755.4073	755.4060	1.3	1.7	22.5	293.6	n/a	n/a	C48 H55 N2 O6

Figure S11: ^{1}H (600 MHz) and ^{13}C (150 MHz) NMR spectra (chloroform-*d* at 298K), mass spectrum, and elemental composition of **4h**.

2,7-bis(2-ethylhexyl)-4,9-bis((4-vinylphenyl)amino)benzo[lmn][3,8]phenanthroline-1,3,6,8(2H,7H)-tetraone (**4i**):

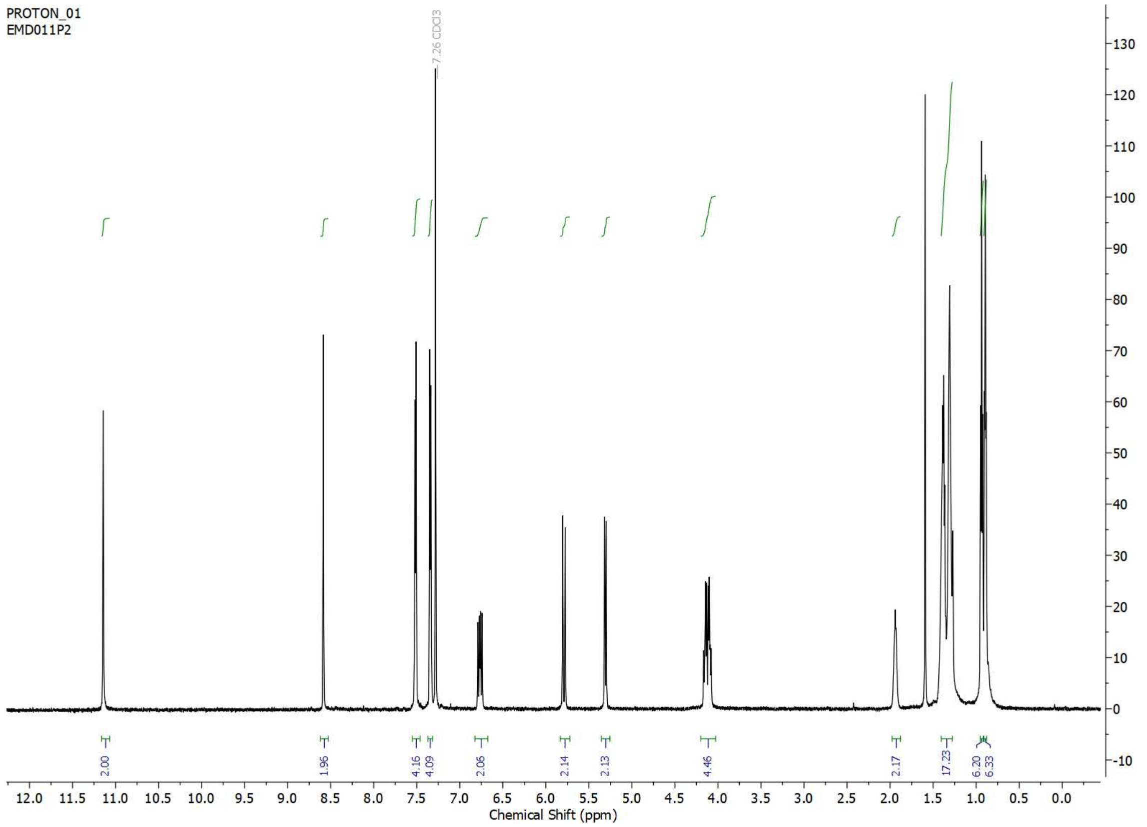

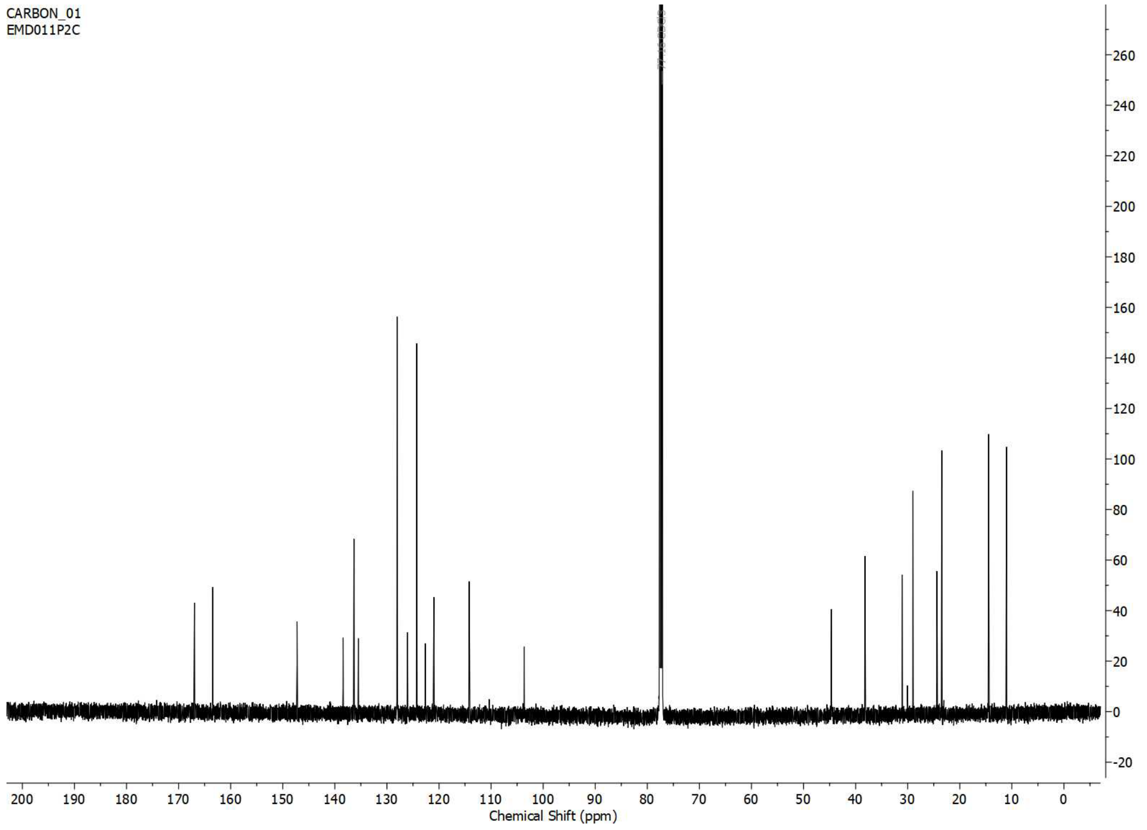

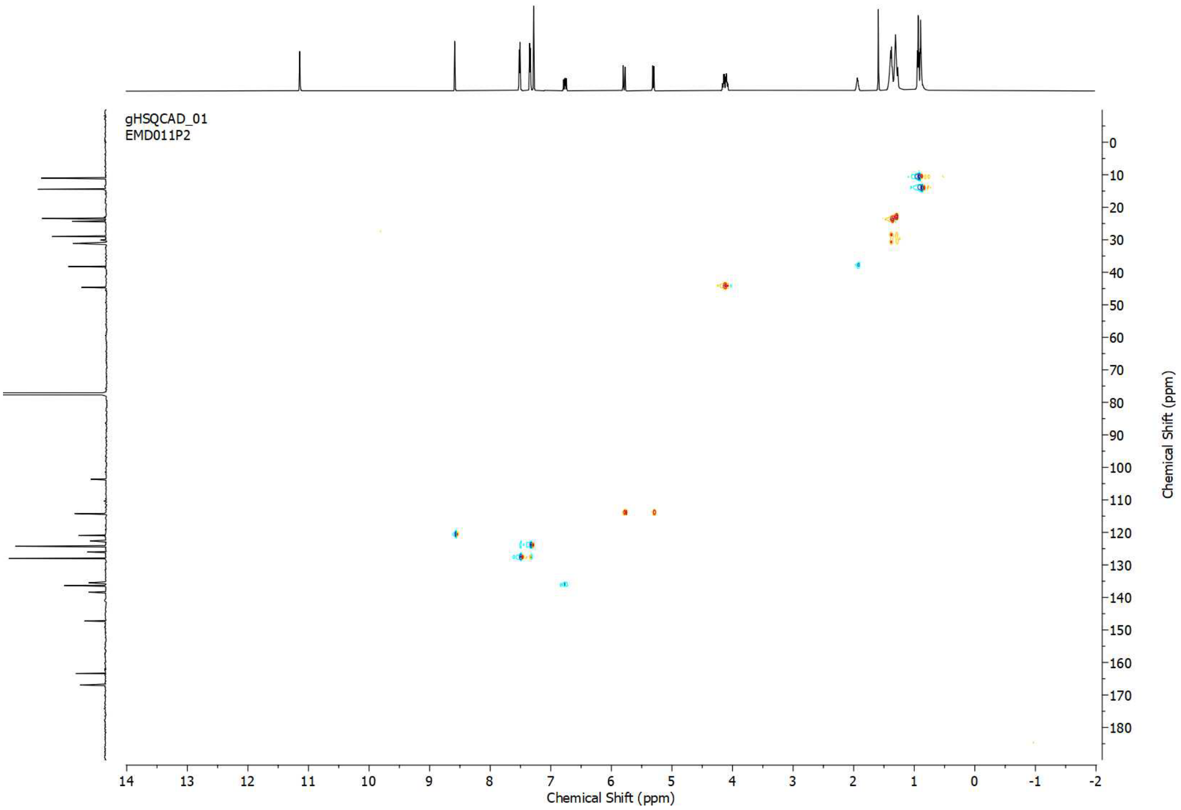
gHSQCAD_01
EMD011P2
Chemical Shift (ppm)
Chemical Shift (ppm)

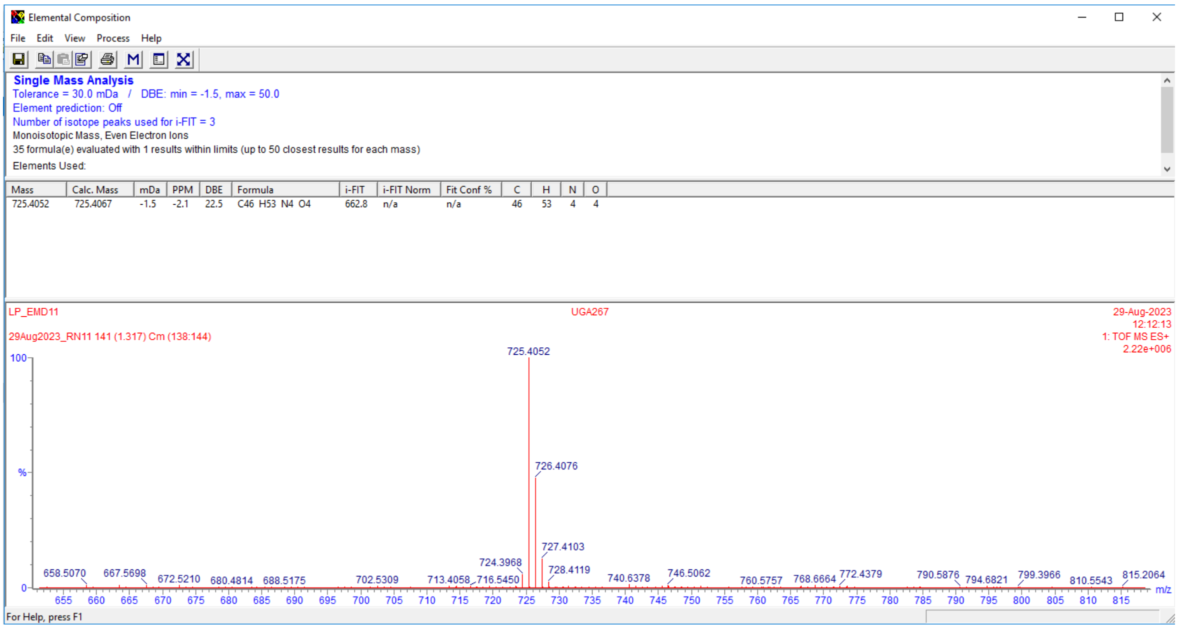
Elemental Composition
File Edit View Process Help
Single Mass Analysis
Tolerance = 30.0 mDa / DBE: min = -1.5, max = 50.0
Element prediction: Off
Number of isotope peaks used for i-FIT = 3
Monoisotopic Mass, Even Electron Ions
35 formula(e) evaluated with 1 results within limits (up to 50 closest results for each mass)
Elements Used:
Mass | Calc. Mass | mDa | PPM | DBE | Formula | i-FIT | i-FIT Norm | Fit Conf % | C | H | N | O
725.4052 | 725.4067 | -1.5 | -2.1 | 22.5 | C46 H53 N4 O4 | 662.8 | n/a | n/a | 46 | 53 | 4 | 4
LP_EMD11
UGA267
29-Aug-2023
12:12:13
29Aug2023_RN11 141 (1.317) Cm (138:144)
1: TOF MS ES+
2.22e+006
725.4052
726.4076
727.4103
724.3968
728.4119
m/z
For Help, press F1

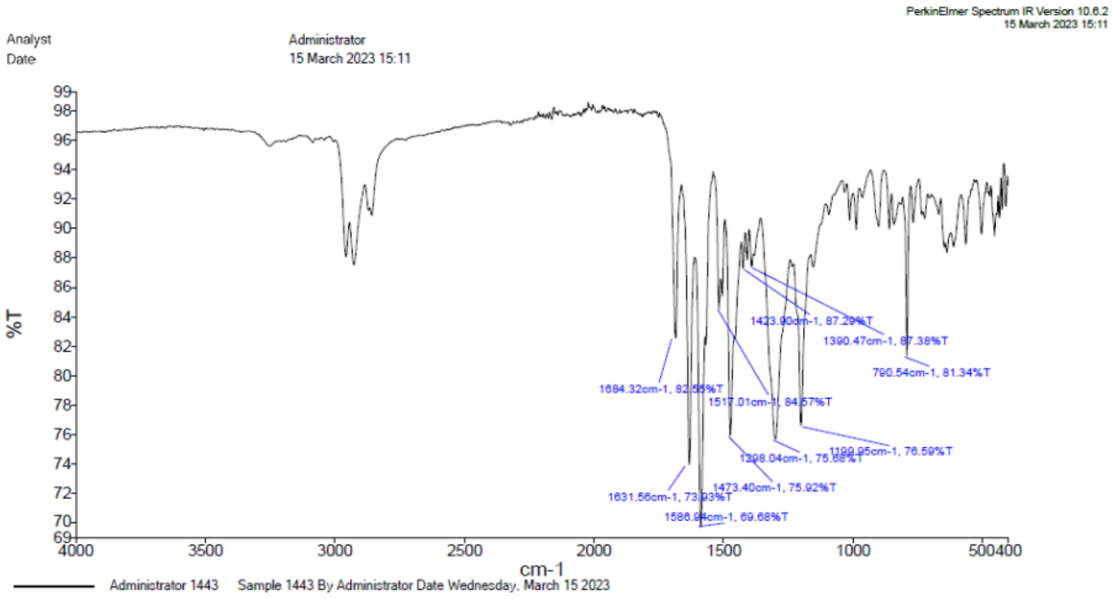

Figure S12: ^{1}H (600 MHz), ^{13}C (150 MHz) and gHSQCAD NMR spectra (chloroform-*d* at 298K), mass spectrum, elemental composition, and IR spectrum of **4i**.

2,7-bis(2-ethylhexyl)-4,9-bis((2-ethylhexyl)amino)benzo[lmn][3,8]phenanthroline-1,3,6,8(2H,7H)-tetraone, **9**

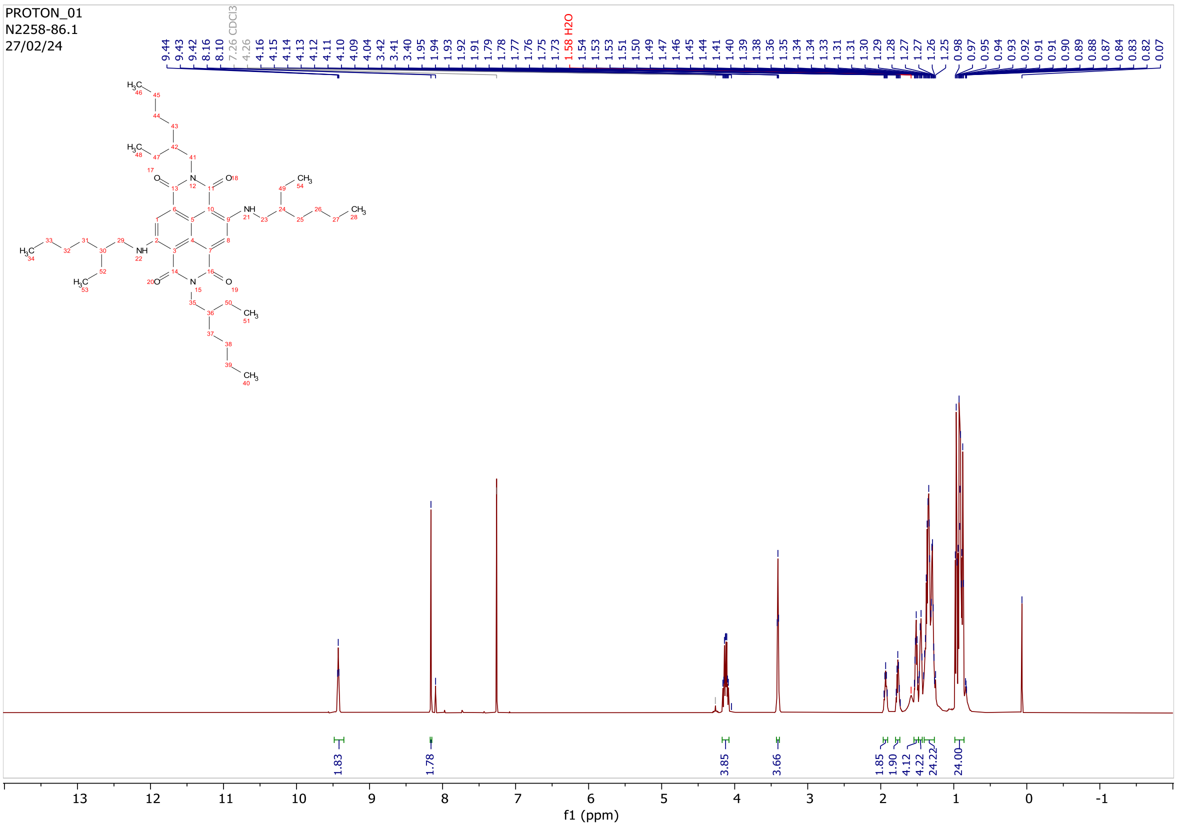

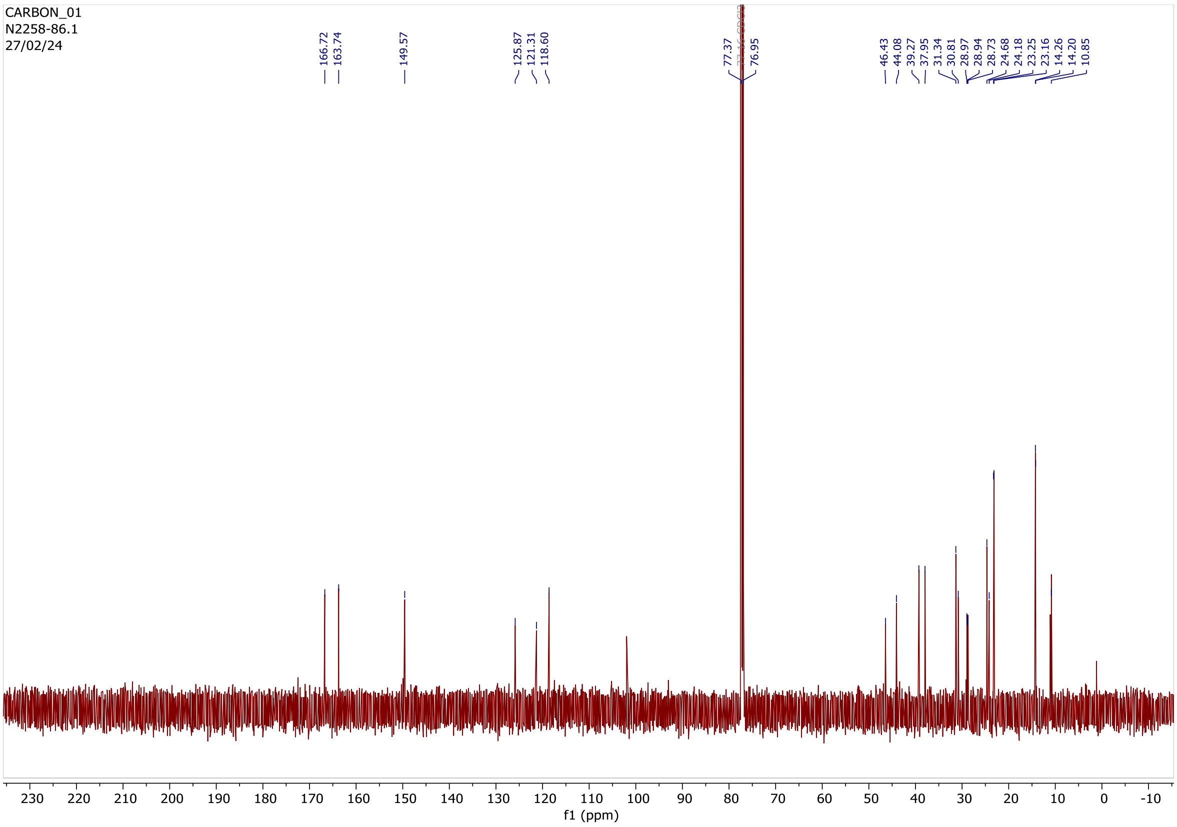

Figure S13: ^{1}H (600 MHz) and ^{13}C (150 MHz) NMR spectra (chloroform-*d* at 298K) of **9.**

4-bromo-2,7-bis(2-ethylhexyl)-9-((2-ethylhexyl)amino)benzo[lmn][3,8]phenanthroline-1,3,6,8(2H,7H)-tetraone, **10**

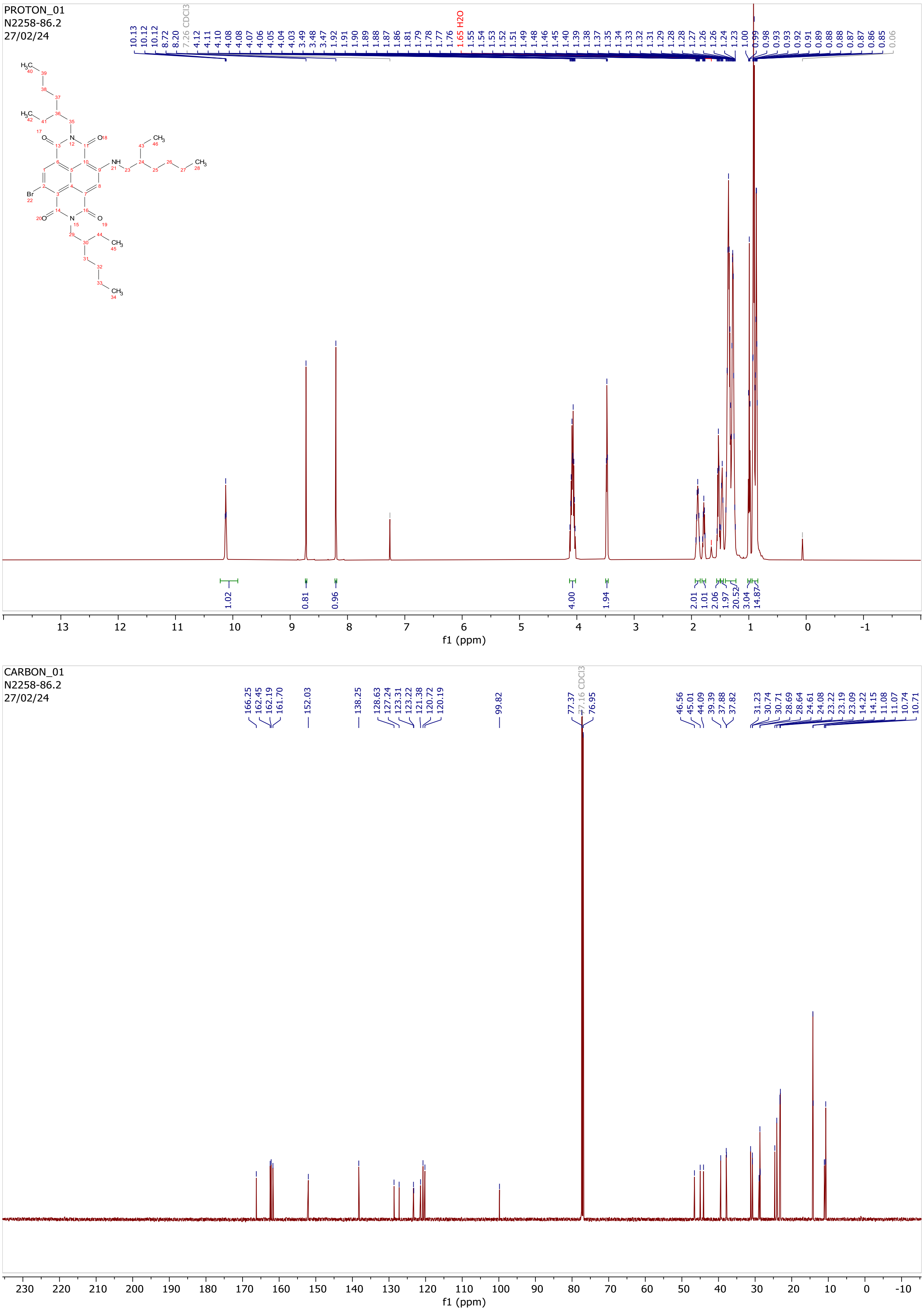

Figure S14: ^{1}H (600 MHz) and ^{13}C (150 MHz) NMR spectra (chloroform-*d* at 298K) of **10.**

2,7-bis(2-ethylhexyl)-4-((2-ethylhexyl)amino)benzo[lmn][3,8]phenanthroline-1,3,6,8(2H,7H)-tetraone, **11**

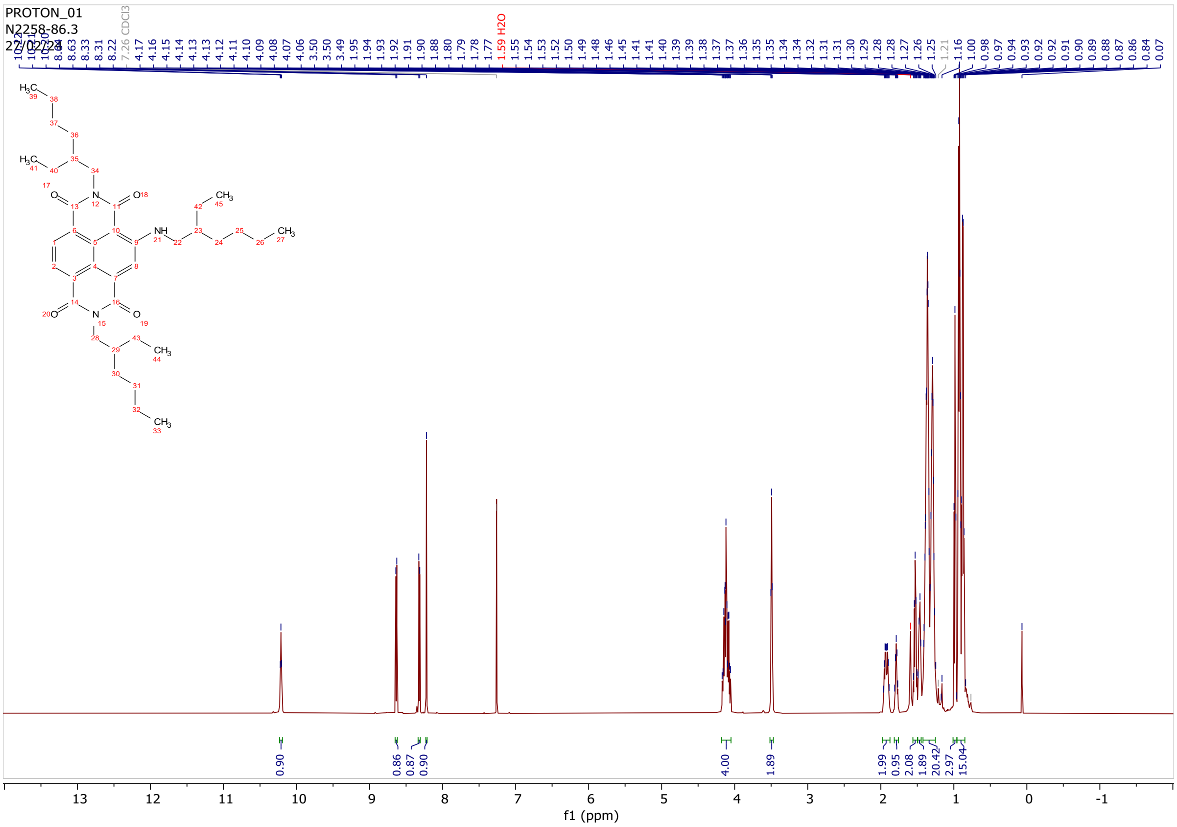

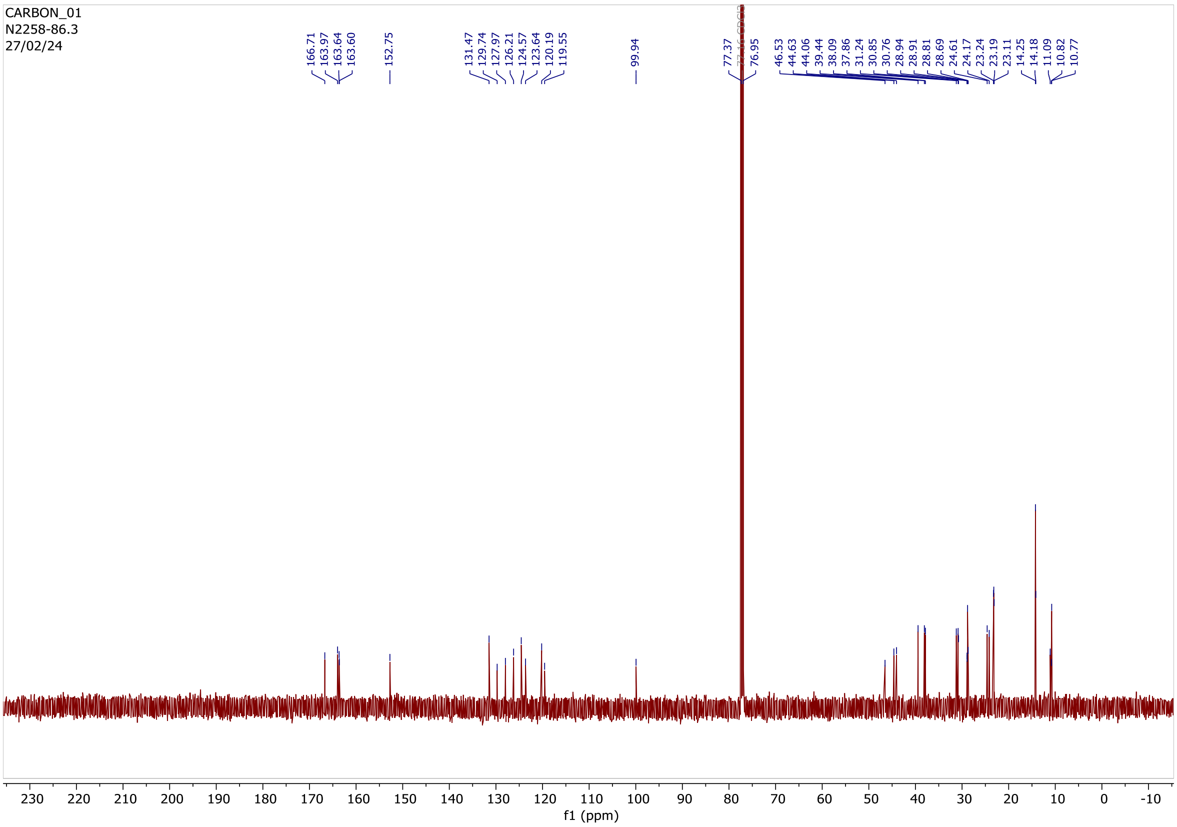

Figure S15: ^{1}H (600 MHz) and ^{13}C (150 MHz) NMR spectra (chloroform-*d* at 298K) of **11.**

S3.0 X-Ray Data

EMD012

b"\n \n

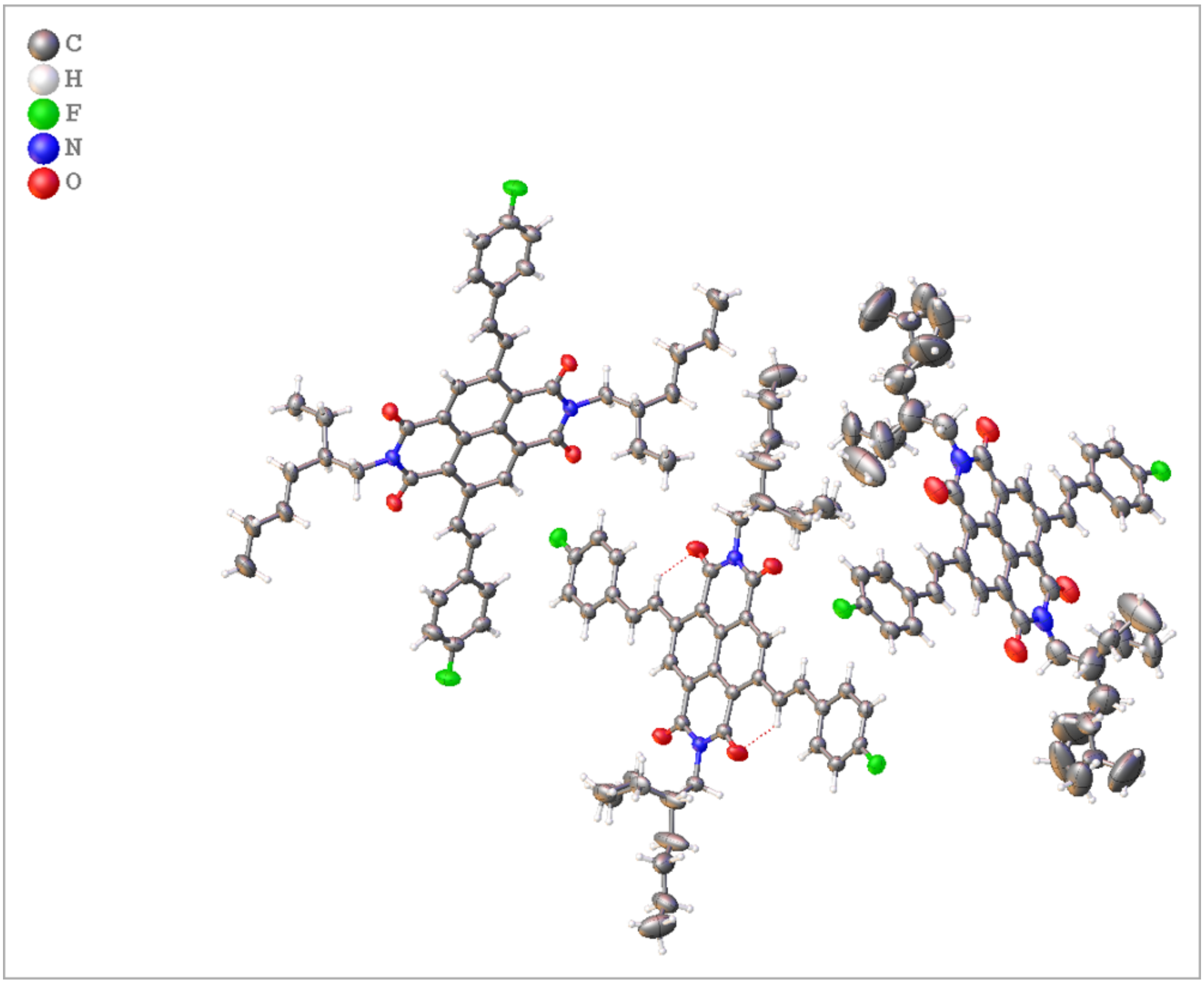

\n "

Table S2 Crystal data and structure refinement for EMD012.

Identification code	EMD012
Empirical formula	$C_{46}H_{48}F_2N_2O_4$
Formula weight	730.86
Temperature/K	100.00(10)
Crystal system	triclinic
Space group	P-1
a/Å	5.08854(19)
b/Å	23.6441(5)

c/Å	24.5654(6)
α/°	110.121(2)
β/°	91.185(3)
γ/°	92.079(2)
Volume/$Å^3$	2771.62(14)
Z	3
ρ_{calc}g/cm^3	1.314
μ/mm^1	0.728
F(000)	1164.0
Crystal size/mm^3	1.684 × 0.05 × 0.004
Radiation	Cu Kα (λ = 1.54184)
2Θ range for data collection/°	3.834 to 134.65
Index ranges	-5 ≤ h ≤ 5, -28 ≤ k ≤ 28, -29 ≤ l ≤ 29
Reflections collected	74733
Independent reflections	9768 [R_{int} = 0.0966, R_{sigma} = 0.0508]
Data/restraints/parameters	9768/706/805
Goodness-of-fit on F^2	1.049
Final R indexes [I>=2σ (I)]	R_1 = 0.0711, wR_2 = 0.1914
Final R indexes [all data]	R_1 = 0.1087, wR_2 = 0.2181
Largest diff. peak/hole / e $Å^{-3}$	0.58/-0.35

Table S3 Fractional Atomic Coordinates (×10⁴) and Equivalent Isotropic Displacement Parameters (Å²×10³) for EMD012. U_{eq} is defined as 1/3 of the trace of the orthogonalised U_{IJ} tensor.

Atom	*x*	*y*	*z*	U(eq)
F17	-3265(4)	4986.3(8)	3207.3(7)	52.4(5)
F44	18724(4)	6683.0(8)	-476.2(8)	60.2(5)
O72	715(4)	6020.2(9)	4230.7(9)	44.4(5)
F74	18986(4)	7824.1(9)	7989.1(8)	68.2(6)
O73	7981(4)	6789.3(9)	5383.8(9)	49.7(5)
O19	15171(4)	6499.0(9)	743.7(9)	52.6(6)
N6	11765(5)	6276.1(10)	1230.7(10)	38.4(5)
O18	8435(5)	6059.6(10)	1721.6(10)	61.3(6)
N63	4352(5)	6405.2(10)	4820.1(10)	39.5(6)
C8	10297(5)	5255.7(11)	253.9(11)	34.9(6)
C1	8844(6)	5362.1(12)	762.1(12)	37.1(6)
C4	12410(6)	5652.7(12)	226.9(12)	36.7(6)
C59	6447(6)	6354.6(13)	5169.1(12)	40.0(7)
C2	6804(6)	4951.9(12)	781.6(12)	37.6(6)
C56	4921(6)	5286.9(12)	4968.1(11)	34.8(6)
C61	1255(6)	4874.6(12)	4289.8(12)	37.4(6)
C57	6719(6)	5774.4(12)	5274.6(11)	35.9(6)
C60	2925(6)	5352.3(12)	4593.9(11)	36.4(6)
O46	11066(6)	9566.4(12)	965.4(15)	88.4(9)
O45	5871(6)	11135.7(12)	1727.5(14)	86.4(9)
C58	8631(5)	5702.3(12)	5660.2(11)	35.6(6)
C7	9590(6)	5912.3(12)	1270.6(12)	40.7(7)
C11	1576(6)	4757.3(12)	1828.0(12)	39.7(7)
C9	5246(6)	5021.4(13)	1291.5(12)	40.8(7)

Table S3 Fractional Atomic Coordinates (×10[4]) and Equivalent Isotropic Displacement Parameters (Å[2]×10[3]) for EMD012. U_{eq} is defined as 1/3 of the trace of the orthogonalised U_{IJ} tensor.

Atom	*x*	*y*	*z*	U(eq)
C55	10405(6)	6189.7(12)	6038.7(12)	38.9(6)
C3	13748(6)	5550.8(12)	-271.5(12)	39.4(7)
C5	13244(6)	6174.4(12)	743.1(12)	40.9(7)
C10	3123(6)	4685.9(13)	1318.0(12)	41.5(7)
C62	2528(6)	5940.5(12)	4525.0(12)	39.4(7)
C14	-1622(6)	4897.2(14)	2754.4(12)	43.4(7)
C54	12575(6)	6103.2(13)	6310.2(12)	38.8(7)
C12	-468(6)	4336.6(13)	1801.2(13)	42.2(7)
C48	14237(6)	6564.4(13)	6740.0(12)	39.0(6)
C52	17907(6)	6811.0(14)	7439.5(12)	46.5(7)
C15	396(6)	5319.3(14)	2811.7(13)	46.4(7)
C13	-2067(6)	4402.2(13)	2264.2(13)	44.1(7)
C16	1985(6)	5246.4(14)	2346.1(13)	44.9(7)
C53	16314(6)	6393.3(13)	7021.7(12)	41.8(7)
C41	16818(7)	7067.5(13)	-504.5(15)	51.0(8)
N33	8366(7)	10331.6(14)	1342.5(17)	74.1(9)
C20	12612(6)	6776.9(13)	1765.6(13)	44.6(7)
C64	3936(7)	7010.4(13)	4789.4(14)	46.9(7)
C49	13821(7)	7179.7(14)	6900.7(14)	51.7(8)
C51	17411(6)	7407.5(14)	7576.2(13)	50.5(8)
C35	5692(8)	10055.5(15)	265.3(17)	67.0(11)
C50	15383(7)	7600.2(15)	7324.1(15)	59.1(9)
C38	13068(7)	7866.3(14)	-541.3(16)	54.5(8)
C40	16082(7)	7097.5(15)	-1025.4(16)	58.7(9)

Table S3 Fractional Atomic Coordinates (×10[4]) and Equivalent Isotropic Displacement Parameters (Å[2]×10[3]) for EMD012. U_{eq} is defined as 1/3 of the trace of the orthogonalised U_{IJ} tensor.

Atom	*x*	*y*	*z*	U(eq)
C37	11136(7)	8303.5(14)	-573.2(18)	60.0(9)
C28	5051(8)	10566.9(15)	739(2)	67.7(10)
C65	5590(8)	7161.4(14)	4328.8(15)	58.3(9)
C39	14187(7)	7501.6(15)	-1044.4(16)	58.0(9)
C34	6405(8)	10707.3(17)	1305(2)	73.3(11)
C31	7659(8)	9665.6(15)	326.2(19)	66.2(10)
C29	6803(8)	9052.6(16)	-676(2)	69.6(11)
C42	15763(8)	7410.3(16)	1.1(16)	65.3(10)
C43	13901(9)	7809.6(16)	-23.1(17)	71.6(11)
C71	7098(8)	6820.4(17)	3294.7(16)	64.6(10)
C66	4836(9)	7798.7(14)	4357.5(16)	65.5(10)
C36	10053(8)	8698.1(15)	-118.6(19)	67.9(11)
C70	5313(8)	6699.2(15)	3732.7(15)	58.9(9)
C30	8187(8)	9148.0(15)	-151(2)	68.3(10)
C67	5782(10)	8313.6(15)	4883.9(18)	73.4(11)
C68	4748(10)	8922.5(15)	4925.6(18)	79.9(13)
C32	9149(8)	9832.4(16)	882(2)	74.6(11)
C23	10376(9)	8216(2)	2836.3(18)	81.9(12)
C21	10967(10)	7317.1(17)	1916(2)	88.5(14)
C69	5903(13)	9439.4(18)	5439(2)	105.8(18)
C47	9825(10)	10495(2)	1903(2)	91.0(13)
C24	11391(11)	8638.0(19)	3406(2)	95.2(15)
C22	12102(11)	7786(2)	2495(2)	122(2)
C26	10920(20)	7545(3)	1401(4)	85(3)

Table S3 Fractional Atomic Coordinates (×10^4) and Equivalent Isotropic Displacement Parameters (Å^2×10^3) for EMD012. U_{eq} is defined as 1/3 of the trace of the orthogonalised U_{IJ} tensor.

Atom	*x*	*y*	*z*	U(eq)
C27	9494(14)	8117(2)	1426(3)	78(2)
C25	9642(12)	9091(3)	3770(2)	135(2)
C6	8523(16)	10282(4)	2336(4)	144(2)
C19	9910(19)	10517(4)	2909(3)	163(3)
C18	5850(20)	9494(4)	2613(5)	131(4)
C17	7740(20)	9655(5)	2182(6)	149(4)
C26A	9650(20)	7585(6)	1539(6)	55(3)
C27A	12040(30)	7959(6)	1411(6)	81(4)
C17A	6250(30)	9897(8)	2188(7)	90(5)
C18A	6060(80)	9185(9)	1970(30)	250(20)
C45	9120(30)	11808(5)	3969(5)	158(5)
C44	10810(30)	11728(4)	3544(6)	194(6)
C33	10730(50)	11206(6)	3123(6)	273(9)
C33A	8390(30)	11046(7)	3217(6)	97(5)
C44A	9470(40)	11356(8)	3749(6)	100(6)
C45A	8840(100)	11140(20)	4152(12)	310(20)

Table S4 Anisotropic Displacement Parameters (Å^2×10^3) for EMD012. The Anisotropic displacement factor exponent takes the form: $-2\pi^2[h^2a^{*2}U_{11}+2hka^*b^*U_{12}+\ldots]$.

Atom	U_{11}	U_{22}	U_{33}	U_{23}	U_{13}	U_{12}
F17	54.3(11)	59.5(11)	47.7(10)	22.3(8)	14.7(8)	10.4(9)
F44	67.1(13)	39.6(10)	70.8(12)	13.7(9)	9.6(10)	13.9(9)
O72	46.9(13)	39.3(11)	48.5(12)	16.5(9)	0.8(10)	6.8(9)
F74	63.5(13)	59.9(12)	61.6(12)	-2.9(10)	-12.5(10)	-0.8(10)
O73	57.7(14)	34.3(11)	56.2(13)	15.2(10)	-4.1(10)	-2.3(10)
O19	55.2(14)	45.0(12)	51.1(13)	8.9(10)	8.3(10)	-8.3(10)
N6	41.9(14)	33.8(12)	38.3(13)	10.7(10)	4.1(10)	2.9(10)
O18	72.2(16)	50.7(13)	48.0(13)	0.4(10)	21.0(11)	-9.5(11)
N63	43.3(14)	29.5(12)	45.5(14)	12.1(10)	3.9(11)	5.7(10)
C8	39.9(16)	30.5(14)	35.5(14)	12.6(11)	1.9(12)	6.6(11)
C1	41.5(17)	33.6(14)	35.9(14)	10.8(12)	2.0(12)	7.2(12)
C4	41.1(17)	32.2(14)	39.0(15)	14.9(12)	0.7(12)	5.5(12)
C59	45.0(17)	33.1(15)	40.3(16)	9.6(12)	7.1(13)	7.3(13)
C2	40.5(17)	34.8(15)	39.9(15)	15.3(12)	3.9(12)	6.1(12)
C56	39.0(16)	31.1(13)	31.7(14)	6.5(11)	10.6(11)	7.0(11)
C61	41.0(17)	35.4(15)	34.6(15)	9.7(12)	8.2(12)	7.2(12)
C57	38.2(16)	31.6(14)	35.1(14)	7.0(11)	9.2(12)	6.1(11)
C60	40.0(16)	32.6(14)	34.8(14)	8.4(12)	8.9(12)	7.1(12)
O46	74(2)	60.1(16)	130(3)	28.1(17)	22.3(17)	21.8(15)
O45	93(2)	51.9(15)	111(2)	19.6(15)	23.6(18)	22.5(14)
C58	39.1(16)	32.2(14)	32.8(14)	7.1(11)	10.0(12)	5.1(12)
C7	46.0(18)	33.8(15)	39.9(16)	9.2(12)	4.2(13)	6.0(12)
C11	40.0(17)	38.2(15)	43.7(16)	16.9(13)	5.8(12)	10.1(12)
C9	45.9(18)	37.9(15)	37.9(15)	11.9(12)	3.1(13)	5.8(13)
C55	42.3(17)	33.0(14)	40.2(15)	10.6(12)	8.1(12)	4.3(12)

Table S4 Anisotropic Displacement Parameters (Å^2×10^3) for EMD012. The Anisotropic displacement factor exponent takes the form: $-2\pi^2[h^2a^{*2}U_{11}+2hka^*b^*U_{12}+\ldots]$.

Atom	U_{11}	U_{22}	U_{33}	U_{23}	U_{13}	U_{12}
C3	43.1(17)	36.1(15)	41.4(16)	15.8(13)	4.3(13)	5.3(12)
C5	45.2(18)	34.6(15)	43.0(16)	13.1(12)	3.2(13)	3.8(13)
C10	47.1(18)	35.8(15)	40.1(16)	10.4(13)	6.9(13)	6.7(13)
C62	42.5(17)	35.1(15)	39.5(15)	10.6(12)	9.6(13)	6.4(12)
C14	45.9(18)	52.3(18)	38.6(16)	22.7(14)	11.2(13)	12.2(14)
C54	44.0(17)	34.6(15)	36.0(15)	9.2(12)	9.8(12)	5.0(12)
C12	47.1(18)	36.1(15)	42.8(16)	11.7(13)	6.7(13)	8.8(13)
C48	37.8(16)	40.6(15)	35.6(15)	8.8(12)	7.5(12)	2.6(12)
C52	44.7(18)	56.1(18)	35.9(16)	11.8(14)	4.3(13)	4.2(14)
C15	54(2)	44.5(17)	37.9(16)	10.4(13)	6.0(14)	7.0(14)
C13	45.7(18)	43.3(16)	48.8(17)	22.2(14)	6.1(14)	6.7(13)
C16	47.0(18)	43.1(16)	45.3(17)	15.4(13)	8.4(14)	5.1(13)
C53	42.8(17)	42.5(16)	37.8(15)	10.2(13)	11.2(13)	4.9(13)
C41	57(2)	30.1(15)	66(2)	15.5(14)	11.7(16)	7.2(14)
N33	70(2)	50.0(17)	105(3)	26.4(17)	25.3(19)	15.9(15)
C20	47.5(19)	37.6(16)	43.1(16)	6.9(13)	0.5(13)	3.2(13)
C64	53(2)	32.0(15)	56.9(19)	16.7(14)	1.9(15)	7.5(13)
C49	53(2)	42.2(17)	54.6(19)	10.1(14)	-4.3(15)	4.6(14)
C51	45.6(19)	49.6(18)	44.0(17)	1.0(14)	1.3(14)	-0.2(14)
C35	63(3)	38.8(17)	107(3)	33(2)	38(2)	11.9(17)
C50	59(2)	41.8(18)	64(2)	3.0(16)	-5.3(17)	5.0(15)
C38	58(2)	35.1(16)	70(2)	16.6(15)	16.6(17)	6.5(14)
C40	66(2)	45.4(18)	58(2)	8.7(16)	11.5(17)	15.3(16)
C37	55(2)	41.8(18)	86(3)	24.1(17)	19.0(18)	9.8(15)
C28	62(2)	39.1(18)	108(3)	30.6(19)	32(2)	11.6(16)

Table S4 Anisotropic Displacement Parameters (Å^2×10^3) for EMD012. The Anisotropic displacement factor exponent takes the form: $-2\pi^2[h^2a^{*2}U_{11}+2hka^*b^*U_{12}+\ldots]$.

Atom	U_{11}	U_{22}	U_{33}	U_{23}	U_{13}	U_{12}
C65	81(3)	38.3(17)	59(2)	21.8(15)	-2.0(18)	0.7(16)
C39	64(2)	48.0(19)	61(2)	15.9(16)	10.2(17)	12.7(16)
C34	69(3)	45(2)	111(3)	31(2)	33(2)	15.8(18)
C31	63(2)	42.6(19)	102(3)	33.9(19)	32(2)	11.0(16)
C29	69(3)	39.9(19)	107(3)	31(2)	36(2)	12.4(17)
C42	86(3)	52(2)	59(2)	18.5(17)	17.4(19)	20.8(19)
C43	96(3)	52(2)	68(2)	18.5(18)	34(2)	30(2)
C71	71(3)	67(2)	65(2)	33.4(19)	10.3(18)	7.9(19)
C66	97(3)	39.6(18)	65(2)	23.8(16)	7(2)	4.9(18)
C36	76(3)	41.4(18)	94(3)	30.4(19)	35(2)	19.9(17)
C70	67(2)	50.8(19)	64(2)	24.6(16)	9.6(17)	10.4(17)
C30	64(2)	42.5(19)	107(3)	32.7(19)	37(2)	14.9(17)
C67	103(3)	38.6(18)	79(3)	21.2(17)	9(2)	-1.3(19)
C68	125(4)	38.2(19)	79(3)	22.3(18)	17(3)	5(2)
C32	64(3)	46(2)	116(3)	28(2)	32(2)	13.7(18)
C23	92(3)	83(3)	70(3)	28(2)	-2(2)	-24(2)
C21	102(3)	46(2)	95(3)	-4.3(19)	-39(3)	18(2)
C69	170(5)	42(2)	98(3)	16(2)	6(3)	-4(3)
C47	80(3)	74(3)	116(4)	25(3)	27(3)	24(2)
C24	110(4)	57(2)	102(3)	9(2)	-25(3)	1(2)
C22	120(4)	67(3)	129(4)	-28(3)	-70(3)	37(3)
C26	106(8)	38(4)	100(6)	8(4)	-19(5)	30(5)
C27	95(6)	48(3)	77(4)	4(3)	-7(4)	22(3)
C25	105(4)	188(7)	78(3)	1(4)	17(3)	5(4)
C6	141(6)	160(6)	151(5)	80(5)	30(4)	-5(5)

Table S4 Anisotropic Displacement Parameters (Å^2×10^3) for EMD012. The Anisotropic displacement factor exponent takes the form: $-2\pi^2[h^2a^{*2}U_{11}+2hka^*b^*U_{12}+\ldots]$.

Atom	U_{11}	U_{22}	U_{33}	U_{23}	U_{13}	U_{12}
C19	209(8)	146(5)	129(5)	37(4)	30(5)	39(5)
C18	164(9)	100(6)	162(9)	84(7)	42(7)	40(6)
C17	136(10)	122(7)	220(11)	99(7)	42(8)	10(6)
C26A	49(8)	48(7)	65(7)	11(5)	5(5)	29(5)
C27A	101(10)	56(8)	82(8)	12(6)	38(7)	33(7)
C17A	103(11)	106(11)	74(10)	43(9)	37(8)	44(7)
C18A	190(30)	118(13)	400(50)	40(20)	-100(30)	44(15)
C45	227(13)	127(9)	142(9)	64(7)	80(8)	88(9)
C44	263(14)	93(6)	244(12)	67(6)	171(11)	71(7)
C33	420(20)	185(9)	160(11)	-18(9)	118(13)	-4(15)
C33A	97(11)	83(9)	105(10)	29(7)	-46(8)	-11(7)
C44A	123(14)	96(11)	66(8)	16(7)	4(8)	-69(10)
C45A	410(40)	400(40)	170(17)	180(20)	20(20)	-50(30)

Table S5 Bond Lengths for EMD012.

Atom	Atom	Length/Å	Atom	Atom	Length/Å
F17	C14	1.368(3)	N33	C34	1.383(5)
F44	C41	1.370(4)	N33	C32	1.402(5)
O72	C62	1.220(3)	N33	C47	1.472(6)
F74	C51	1.365(4)	C20	C21	1.495(5)
O73	C59	1.223(4)	C64	C65	1.553(5)
O19	C5	1.223(4)	C49	C50	1.378(5)
N6	C7	1.403(4)	C51	C50	1.363(5)
N6	C5	1.382(4)	C35	C35[3]	1.406(9)
N6	C20	1.478(4)	C35	C28	1.414(5)
O18	C7	1.213(3)	C35	C31	1.424(5)
N63	C59	1.389(4)	C38	C37	1.472(5)
N63	C62	1.394(4)	C38	C39	1.388(5)
N63	C64	1.481(3)	C38	C43	1.385(5)
C8	C8[1]	1.425(5)	C40	C39	1.393(5)
C8	C1	1.416(4)	C37	C36	1.329(5)
C8	C4	1.419(4)	C28	C34	1.462(6)
C1	C2	1.408(4)	C28	C29[3]	1.370(5)
C1	C7	1.491(4)	C65	C66	1.547(4)

Table S5 Bond Lengths for EMD012.

Atom	Atom	Length/Å	Atom	Atom	Length/Å
C4	C3	1.365(4)	C65	C70	1.496(5)
C4	C5	1.474(4)	C31	C30	1.412(5)
C59	C57	1.492(4)	C31	C32	1.469(6)
C2	C9	1.460(4)	C29	C30	1.399(6)
C2	C3[1]	1.414(4)	C42	C43	1.377(5)
C56	C56[2]	1.423(5)	C71	C70	1.516(5)
C56	C57	1.425(4)	C66	C67	1.495(5)
C56	C60	1.404(4)	C36	C30	1.473(5)
C61	C60	1.371(4)	C67	C68	1.522(5)
C61	C58[2]	1.414(4)	C68	C69	1.512(6)
C57	C58	1.399(4)	C23	C24	1.480(6)
C60	C62	1.478(4)	C23	C22	1.420(7)
O46	C32	1.231(5)	C21	C22	1.557(6)
O45	C34	1.219(5)	C21	C26	1.536(9)
C58	C55	1.470(4)	C21	C26A	1.456(12)
C11	C10	1.457(4)	C47	C6	1.482(8)
C11	C12	1.397(4)	C24	C25	1.476(7)
C11	C16	1.400(4)	C26	C27	1.542(8)

Table S5 Bond Lengths for EMD012.

Atom	Atom	Length/Å	Atom	Atom	Length/Å
C9	C10	1.333(4)	C6	C19	1.477(10)
C55	C54	1.336(4)	C6	C17	1.436(11)
C14	C15	1.376(4)	C6	C17A	1.405(16)
C14	C13	1.368(4)	C19	C33	1.568(15)
C54	C48	1.457(4)	C19	C33A	1.479(15)
C12	C13	1.381(4)	C18	C17	1.577(12)
C48	C53	1.397(4)	C26A	C27A	1.579(15)
C48	C49	1.396(4)	C17A	C18A	1.581(19)
C52	C53	1.377(4)	C45	C44	1.333(13)
C52	C51	1.367(5)	C44	C33	1.308(13)
C15	C16	1.380(4)	C33A	C44A	1.353(15)
C41	C40	1.352(5)	C44A	C45A	1.30(2)
C41	C42	1.362(5)			

[1]2-X,1-Y,-Z; [2]1-X,1-Y,1-Z; [3]1-X,2-Y,-Z

Table S6 Bond Angles for EMD012.

Atom	Atom	Atom	Angle/˚	Atom	Atom	Atom	Angle/˚
C7	N6	C20	116.6(2)	C32	N33	C47	118.2(4)
C5	N6	C7	124.7(2)	N6	C20	C21	115.3(3)
C5	N6	C20	118.6(2)	N63	C64	C65	114.1(2)
C59	N63	C62	125.1(2)	C50	C49	C48	121.2(3)
C59	N63	C64	117.1(2)	F74	C51	C52	118.4(3)
C62	N63	C64	117.6(2)	C50	C51	F74	118.9(3)
C1	C8	$C8^{1}$	121.4(3)	C50	C51	C52	122.6(3)
C1	C8	C4	121.6(2)	$C35^{3}$	C35	C28	117.6(4)
C4	C8	$C8^{1}$	117.0(3)	$C35^{3}$	C35	C31	121.4(4)
C8	C1	C7	117.7(3)	C28	C35	C31	121.0(4)
C2	C1	C8	120.3(3)	C51	C50	C49	118.9(3)
C2	C1	C7	122.0(3)	C39	C38	C37	119.8(3)
C8	C4	C5	120.4(2)	C43	C38	C37	122.8(3)
C3	C4	C8	120.8(3)	C43	C38	C39	117.4(3)
C3	C4	C5	118.8(3)	C41	C40	C39	118.5(3)
O73	C59	N63	118.8(3)	C36	C37	C38	125.0(4)
O73	C59	C57	122.5(3)	C35	C28	C34	120.5(4)
N63	C59	C57	118.7(3)	$C29^{3}$	C28	C35	120.8(4)
C1	C2	C9	123.8(3)	$C29^{3}$	C28	C34	118.7(4)
C1	C2	$C3^{1}$	117.0(3)	C66	C65	C64	106.0(3)
$C3^{1}$	C2	C9	119.1(3)	C70	C65	C64	114.4(3)
$C56^{2}$	C56	C57	121.0(3)	C70	C65	C66	113.7(3)
C60	C56	$C56^{2}$	117.4(3)	C38	C39	C40	121.1(3)
C60	C56	C57	121.6(2)	O45	C34	N33	120.5(5)
C60	C61	$C58^{2}$	123.1(3)	O45	C34	C28	122.6(4)

Table S6 Bond Angles for EMD012.

Atom	Atom	Atom	Angle/˚	Atom	Atom	Atom	Angle/˚
C56	C57	C59	117.3(2)	N33	C34	C28	116.9(4)
C58	C57	C59	122.4(3)	C35	C31	C32	118.3(3)
C58	C57	C56	120.4(2)	C30	C31	C35	119.4(4)
C56	C60	C62	121.0(3)	C30	C31	C32	122.2(4)
C61	C60	C56	120.9(3)	C28[3]	C29	C30	122.8(4)
C61	C60	C62	118.0(3)	C41	C42	C43	118.1(4)
C61[2]	C58	C55	118.1(3)	C42	C43	C38	122.1(3)
C57	C58	C61[2]	117.1(3)	C67	C66	C65	116.8(3)
C57	C58	C55	124.7(2)	C37	C36	C30	124.8(4)
N6	C7	C1	118.5(2)	C65	C70	C71	114.4(3)
O18	C7	N6	118.0(3)	C31	C30	C36	123.3(4)
O18	C7	C1	123.5(3)	C29	C30	C31	117.9(4)
C12	C11	C10	119.6(3)	C29	C30	C36	118.7(4)
C12	C11	C16	117.7(3)	C66	C67	C68	115.0(4)
C16	C11	C10	122.7(3)	C69	C68	C67	113.5(4)
C10	C9	C2	126.5(3)	O46	C32	N33	117.9(5)
C54	C55	C58	124.4(3)	O46	C32	C31	124.1(4)
C4	C3	C2[1]	123.4(3)	N33	C32	C31	118.0(4)
O19	C5	N6	121.4(3)	C22	C23	C24	118.0(4)
O19	C5	C4	121.7(3)	C20	C21	C22	108.0(3)
N6	C5	C4	116.9(3)	C20	C21	C26	108.4(5)
C9	C10	C11	126.3(3)	C26	C21	C22	114.5(5)
O72	C62	N63	121.5(3)	C26A	C21	C20	130.0(7)
O72	C62	C60	122.5(3)	C26A	C21	C22	113.8(7)
N63	C62	C60	116.0(3)	N33	C47	C6	114.4(5)

Table S6 Bond Angles for EMD012.

Atom	Atom	Atom	Angle/°	Atom	Atom	Atom	Angle/°
F17	C14	C15	118.0(3)	C25	C24	C23	119.2(5)
F17	C14	C13	119.1(3)	C23	C22	C21	117.6(4)
C13	C14	C15	122.9(3)	C21	C26	C27	121.7(7)
C55	C54	C48	126.8(3)	C19	C6	C47	112.0(7)
C13	C12	C11	121.6(3)	C17	C6	C47	119.4(7)
C53	C48	C54	119.6(3)	C17	C6	C19	111.6(8)
C49	C48	C54	123.0(3)	C17A	C6	C47	121.4(10)
C49	C48	C53	117.3(3)	C17A	C6	C19	126.6(10)
C51	C52	C53	118.1(3)	C6	C19	C33	115.1(10)
C14	C15	C16	118.2(3)	C6	C19	C33A	102.1(8)
C14	C13	C12	118.2(3)	C6	C17	C18	115.7(9)
C15	C16	C11	121.3(3)	C21	C26A	C27A	99.9(9)
C52	C53	C48	121.8(3)	C6	C17A	C18A	128(2)
C40	C41	F44	119.3(3)	C33	C44	C45	118.2(14)
C40	C41	C42	122.8(3)	C44	C33	C19	148.0(14)
C42	C41	F44	117.9(3)	C44A	C33A	C19	111.5(14)
C34	N33	C32	124.9(4)	C45A	C44A	C33A	115(2)
C34	N33	C47	116.7(4)				

[1]2-X,1-Y,-Z; [2]1-X,1-Y,1-Z; [3]1-X,2-Y,-Z

Table S7 Hydrogen Atom Coordinates (Å×10^4) and Isotropic Displacement Parameters (Å^2×10^3) for EMD012.

Atom	*x*	*y*	*z*	U(eq)
H61	-41.65	4934.02	4033.25	45
H9	5802.37	5337.12	1639.09	49
H55	9980.01	6593.3	6094.14	47
H3	15121.04	5832.25	-275.68	47
H10	2564.02	4369.88	970.8	50
H54	13086.9	5698.83	6212.81	47
H12	-763.64	3997.39	1456.23	51
H52	19312.19	6688.07	7627.47	56
H15	688.01	5651.83	3162.32	56
H13	-3440.12	4111.44	2242.93	53
H16	3385.04	5534.14	2377.76	54
H53	16635.81	5977.16	6922.12	50
H20A	14447.71	6905.34	1724.16	53
H20B	12606.94	6623.75	2093.43	53
H64A	4376.98	7317.33	5174.79	56
H64B	2048.75	7038.19	4700.46	56
H49	12434.86	7311.09	6714.76	62
H50	15051.62	8017.07	7438.27	71
H40	16841.52	6848.64	-1371.01	70
H37	10624.11	8303.66	-947.3	72
H65	7482.58	7189.04	4457.22	70

Table S7 Hydrogen Atom Coordinates (Å×10⁴) and Isotropic Displacement Parameters (Å²×10³) for EMD012.

Atom	*x*	*y*	*z*	U(eq)
H39	13651.8	7527.87	-1407.37	70
H29	7163.89	8709.45	-1001.32	83
H42	16298.33	7374.97	360.42	78
H43	13162.33	8054.3	327.09	86
H71A	6907.24	6483.24	2925.67	97
H71B	8929.53	6863.49	3439.98	97
H71C	6611.74	7192.81	3235.44	97
H66A	5525.53	7871.27	4012.14	79
H66B	2893.61	7801.76	4329.34	79
H36	10516.72	8689.24	254.84	81
H70A	3464.65	6678.36	3590.81	71
H70B	5691.76	6300.4	3756.4	71
H67A	7728.62	8343.56	4888.86	88
H67B	5263.63	8223.05	5232.51	88
H68A	5149	9001.94	4565.13	96
H68B	2810.79	8905.07	4953.67	96
H23A	8804.16	7993.61	2904.2	98
H23B	9780.47	8459.17	2603.65	98
H21	9130.18	7192.39	1977.16	106
H21A	9396.18	7157.27	2064.3	106
H69A	7804.68	9481.87	5398.09	159
H69B	5569.23	9358.05	5797.33	159
H69C	5084.23	9813.2	5454.74	159
H47A	10080.4	10939.08	2067.47	109
H47B	11588.06	10324.78	1834.22	109

Table S7 Hydrogen Atom Coordinates (Å×10[4]) and Isotropic Displacement Parameters (Å[2]×10[3]) for EMD012.

Atom	*x*	*y*	*z*	U(eq)
H24A	11989.36	8393.09	3636.28	114
H24B	12966.99	8857.94	3335.94	114
H22A	12796.4	7561.85	2736.96	146
H22B	13615.34	8008.73	2403.36	146
H26A	10150.76	7211.38	1062.76	103
H26B	12775.31	7608.94	1313.7	103
H27A	9565.79	8170.11	1047.44	117
H27B	10356.77	8469.09	1723.09	117
H27C	7653.7	8076.66	1521.67	117
H25A	9267.64	9384.96	3582.29	203
H25B	10507.09	9297.76	4150.7	203
H25C	7992.07	8889.25	3818.71	203
H6A	6821.78	10490.08	2399.97	173
H6B	7405.77	10640.67	2447.52	173
H19A	11512.98	10287.08	2892.86	195
H19B	8760.71	10440.12	3198.72	195
H19C	11767.72	10635.43	2874.49	195
H19D	9848.71	10215.91	3106.88	195
H18A	4126.81	9659.81	2593.79	196
H18B	6600.61	9666.25	3009.22	196
H18C	5641.65	9054.42	2503.59	196
H17A	9348.58	9423.57	2153.21	178
H17B	6861.02	9518.38	1792.75	178
H26C	8240.19	7849.58	1740.57	67
H26D	8898.39	7275.19	1180.59	67

Table S7 Hydrogen Atom Coordinates (Å×10[4]) and Isotropic Displacement Parameters (Å[2]×10[3]) for EMD012.

Atom	*x*	*y*	*z*	U(eq)
H27D	11478.73	8140.97	1127.24	122
H27E	13473.47	7689.88	1254.71	122
H27F	12655.54	8276.82	1771.14	122
H17C	5308.48	10005.13	1883.51	108
H17D	5155.86	10029.84	2533.29	108
H18D	7427.76	9023.02	1694.16	375
H18E	4322.86	9038.8	1785.45	375
H18F	6296.46	9050.2	2305.24	375
H45A	8119.36	12165.76	4010.17	237
H45B	10093.77	11863.7	4333.79	237
H45C	7909.07	11453.33	3876.06	237
H44A	10527.86	12042.11	3369.65	232
H44B	12610.5	11801.6	3721.45	232
H33A	12609.65	11182.33	3024.95	328
H33B	9870.23	11318.51	2812.12	328
H33C	8317.33	11315.81	2985.35	116
H33D	6558.72	10910.91	3257.07	116
H44C	11406.72	11363.53	3721.53	120
H44D	8924.72	11777.96	3866.32	120
H45D	7311.64	10859.23	4019.41	463
H45E	8418.75	11472.45	4503.67	463
H45F	10329.88	10929.29	4238.11	463

Table S8 Atomic Occupancy for EMD012.

Atom	*Occupancy*	Atom	*Occupancy*	Atom	*Occupancy*
H21	0.661(10)	H21A	0.339(10)	C26	0.661(10)
H26A	0.661(10)	H26B	0.661(10)	C27	0.661(10)
H27A	0.661(10)	H27B	0.661(10)	H27C	0.661(10)
H6A	0.724(12)	H6B	0.276(12)	H19A	0.690(9)
H19B	0.690(9)	H19C	0.310(9)	H19D	0.310(9)
C18	0.724(12)	H18A	0.724(12)	H18B	0.724(12)
H18C	0.724(12)	C17	0.724(12)	H17A	0.724(12)
H17B	0.724(12)	C26A	0.339(10)	H26C	0.339(10)
H26D	0.339(10)	C27A	0.339(10)	H27D	0.339(10)
H27E	0.339(10)	H27F	0.339(10)	C17A	0.276(12)
H17C	0.276(12)	H17D	0.276(12)	C18A	0.276(12)
H18D	0.276(12)	H18E	0.276(12)	H18F	0.276(12)
C45	0.690(9)	H45A	0.690(9)	H45B	0.690(9)
H45C	0.690(9)	C44	0.690(9)	H44A	0.690(9)
H44B	0.690(9)	C33	0.690(9)	H33A	0.690(9)
H33B	0.690(9)	C33A	0.310(9)	H33C	0.310(9)
H33D	0.310(9)	C44A	0.310(9)	H44C	0.310(9)
H44D	0.310(9)	C45A	0.310(9)	H45D	0.310(9)
H45E	0.310(9)	H45F	0.310(9)		

Experimental

Single crystals of $C_{46}H_{48}F_2N_2O_4$ (**4d**) were grown by slow diffusion of hexane into a solution of 4d in dichloromethane. A suitable crystal was selected and analysed on a XtaLAB AFC11 (RCD3): quarter-chi single diffractometer. The crystal was kept at 100.00(10) K during data collection. Using Olex2 [1], the structure was solved with the SHELXT [2] structure solution program using Intrinsic Phasing and refined with the SHELXL [3] refinement package using Least Squares minimisation.

1. Dolomanov, O.V., Bourhis, L.J., Gildea, R.J, Howard, J.A.K. & Puschmann, H. (2009), J. Appl. Cryst. 42, 339-341.
2. Sheldrick, G.M. (2015). Acta Cryst. A71, 3-8.
3. Sheldrick, G.M. (2015). Acta Cryst. C71, 3-8.

Crystal structure determination of 4d

Crystal Data for $C_{46}H_{48}F_2N_2O_4$ (M=730.86 g/mol): triclinic, space group P-1 (no. 2), a = 5.08854(19) Å, b = 23.6441(5) Å, c = 24.5654(6) Å, α = 110.121(2)°, β = 91.185(3)°, γ = 92.079(2)°, V = 2771.62(14) Å^3, Z = 3, T = 100.00(10) K, μ(Cu Kα) = 0.728 mm^{-1}, $Dcalc$ = 1.314 g/cm^3, 74733 reflections measured (3.834° ≤ 2Θ ≤ 134.65°), 9768 unique (R_{int} = 0.0966, R_{sigma} = 0.0508) which were used in all calculations. The final R_1 was 0.0711 (I > 2σ(I)) and wR_2 was 0.2181 (all data).

Refinement model description

Number of restraints - 706, number of constraints - unknown.

Details:

```
1. Fixed Uiso
 At 1.2 times of:
  All C(H) groups, All C(H,H) groups, All C(H,H,H,H) groups
 At 1.5 times of:
  All C(H,H,H) groups
2. Restrained distances
 C21-C26 ≈ C21-C26A
 with sigma of 0.02
 C21-C27 ≈ C21-C27A
 with sigma of 0.04
 C26-C27 ≈ C26A-C27A
 with sigma of 0.02
 C6-C17 ≈ C6-C17A
 with sigma of 0.02
 C18-C17 ≈ C18A-C17A
 with sigma of 0.02
 C6-C18 ≈ C6-C18A
 with sigma of 0.04
 C19-C44 ≈ C19-C44A
 with sigma of 0.04
 C19-C33 ≈ C19-C33A
 with sigma of 0.02
 C44-C33 ≈ C44A-C33A
 with sigma of 0.02
 C45-C33 ≈ C45A-C33A
 with sigma of 0.04
 C45-C44 ≈ C45A-C44A
 with sigma of 0.02
3. Rigid bond restraints
 C45A, C44A, C33A, C19
 with sigma for 1-2 distances of 0.01 and sigma for 1-3 distances of 0.01
 C45, C44, C33, C19
 with sigma for 1-2 distances of 0.01 and sigma for 1-3 distances of 0.01
4. Uiso/Uaniso restraints and constraints
H21A ≈ C26A ≈ H26C ≈ H27F ≈ H26D ≈ C27A ≈ H27D ≈
H27E ≈ C45A ≈ C44A ≈ C33A ≈ C19: within 1.7A with sigma of 0.04
and sigma for terminal atoms of 0.08 within 1.7A
C19 ≈ C33 ≈ C44 ≈ C45: within 1.7A with sigma of 0.04 and sigma for
terminal atoms of 0.08 within 1.7A
Uanis(C18A) ≈ Ueq: with sigma of 0.02 and sigma for terminal atoms of 0.05
Uanis(C45A) ≈ Ueq: with sigma of 0.02 and sigma for terminal atoms of 0.05
5. Rigid body (RIGU) restrains
 C66, O72, C62, N63, C60, C59, C64, C56, C61, O73, C57, H64A, H64B, C65, H61,
 C58, H65, C70, C55, H66A, H66B, C67, C71, H70A, H70B, H55, C54, H67A, H67B,
 C68, H71A, H71B, H71C, H54, C48, H68A, H68B, C69, C53, C49, H69A, H69B, H69C,
 H53, C52, H49, C50, H52, C51, H50, F74
 with sigma for 1-2 distances of 0.004 and sigma for 1-3 distances of 0.004
 C7, F17, C14, C15, C13, H15, C16, C12, H13, C11, H16, H12, C10, C9, H10, H9,
 C2, C1, C8, C4, N6, O18, C3, C5, C20, H3, O19, H20A, H20B, C21, H21, H21A, C22,
  C26, C26A, C23, H22A, H22B, H26A, H26B, C27, H26C, H26D, C27A, H23A, H23B,
 C24, H27A, H27B, H27C, H27D, H27E, H27F, H24A, H24B, C25, H25A, H25B, H25C
 with sigma for 1-2 distances of 0.004 and sigma for 1-3 distances of 0.004
 O45, F44, C41, C40, C42, H40, C39, H42, C43, C38, H39, H43, C37, H37, C36,
 H36, C30, C31, C29, C35, C32, H29, C28, N33, O46, C34, C47, C6, C19, C17, C17A,
  C18, C18A
```

```
 with sigma for 1-2 distances of 0.004 and sigma for 1-3 distances of 0.004
6. Others
 Sof(H21A)=Sof(C26A)=Sof(H26C)=Sof(H26D)=Sof(C27A)=Sof(H27D)=Sof(H27E)=
 Sof(H27F)=1-FVAR(1)
 Sof(H21)=Sof(C26)=Sof(H26A)=Sof(H26B)=Sof(C27)=Sof(H27A)=Sof(H27B)=Sof(H27C)=
 FVAR(1)
 Sof(H6B)=Sof(C17A)=Sof(H17C)=Sof(H17D)=Sof(C18A)=Sof(H18D)=Sof(H18E)=
 Sof(H18F)=1-FVAR(2)
 Sof(H6A)=Sof(C18)=Sof(H18A)=Sof(H18B)=Sof(H18C)=Sof(C17)=Sof(H17A)=Sof(H17B)=
 FVAR(2)
 Sof(H19C)=Sof(H19D)=Sof(C33A)=Sof(H33C)=Sof(H33D)=Sof(C44A)=Sof(H44C)=
 Sof(H44D)=Sof(C45A)=Sof(H45D)=Sof(H45E)=Sof(H45F)=1-FVAR(3)
 Sof(H19A)=Sof(H19B)=Sof(C45)=Sof(H45A)=Sof(H45B)=Sof(H45C)=Sof(C44)=Sof(H44A)=
 Sof(H44B)=Sof(C33)=Sof(H33A)=Sof(H33B)=FVAR(3)
7.a Ternary CH refined with riding coordinates:
 C65(H65), C21(H21), C21(H21A), C6(H6A), C6(H6B)
7.b Secondary CH2 refined with riding coordinates:
 C20(H20A,H20B), C64(H64A,H64B), C66(H66A,H66B), C70(H70A,H70B), C67(H67A,
 H67B), C68(H68A,H68B), C23(H23A,H23B), C47(H47A,H47B), C24(H24A,H24B),
 C22(H22A,H22B), C26(H26A,H26B), C19(H19A,H19B), C19(H19C,H19D), C17(H17A,H17B),
  C26A(H26C,H26D), C17A(H17C,H17D), C44(H44A,H44B), C33(H33A,H33B), C33A(H33C,
 H33D), C44A(H44C,H44D)
7.c Aromatic/amide H refined with riding coordinates:
 C61(H61), C9(H9), C55(H55), C3(H3), C10(H10), C54(H54), C12(H12), C52(H52),
 C15(H15), C13(H13), C16(H16), C53(H53), C49(H49), C50(H50), C40(H40), C37(H37),
  C39(H39), C29(H29), C42(H42), C43(H43), C36(H36)
7.d Idealised Me refined as rotating group:
 C71(H71A,H71B,H71C), C69(H69A,H69B,H69C), C27(H27A,H27B,H27C), C25(H25A,H25B,
 H25C), C18(H18A,H18B,H18C), C27A(H27D,H27E,H27F), C18A(H18D,H18E,H18F),
 C45(H45A,H45B,H45C), C45A(H45D,H45E,H45F)
```

This report has been created with Olex2, compiled on 2022.04.12 svn.rca3783a0 for Rigaku Oxford Diffraction. Please let us know if there are any errors or if you would like to have additional features.

S4.0 References

[1] M. Sasikumar, Y. V. Suseela, T. Govindaraju, *Asian J. Org. Chem.* **2013**, *2*, 779–785.
